# Supplementary material for: XPF activates break-induced telomere synthesis
Source: Nat Commun. 2022 Oct 2;13:5781. doi: 10.1038/s41467-022-33428-0 (PMC9527253; doi:10.1038/s41467-022-33428-0)
Supplement: Supplementary file 1 — Supplementary Information [file 41467_2022_33428_MOESM1_ESM.pdf]

Supplementary Information

XPF activates break-induced telomere synthesis

Chia-Yu Guh[1,#], Hong-Jhih Shen[1,#], Liv WeiChien Chen[1,#], Pei-Chen Chiu[1,#], I-Hsin Liao[1], Chen-Chia Lo[1], Yunfei Chen[1], Yu-Hung Hsieh[1], Ting-Chia Chang[1], Chien-Ping Yen[1], Yi-Yun Chen[2], Tom Wei-Wu Chen[3], Liuh-Yow Chen[4], Ching-Shyi Wu[5], Jean-Marc Egly[6,7] and Hsueh-Ping Catherine Chu[1,*]

[1] Institute of Molecular and Cellular Biology, National Taiwan University, No. 1 Sec. 4 Roosevelt Road, Taipei, Taiwan.

[2] Institute of Biological Chemistry, Academia Sinica, Taipei, Taiwan.

[3]Department of Oncology, National Taiwan University Hospital and Graduate Institute of Oncology, National Taiwan University College of Medicine, Taipei, Taiwan.

[4]Institute of Molecular Biology, Academia Sinica, Taipei, Taiwan.

[5]Department of Pharmacology, National Taiwan University, Taipei, Taiwan.

[6]Department of Functional Genomics and Cancer, IGBMC, CNRS/INSERM/University of Strasbourg, Strasbourg, France.

[7]College of Medicine, National Taiwan University, Taipei, Taiwan.

[#]These authors contributed equally.

* To whom correspondence should be addressed. Email: cchu2017@ntu.edu.tw

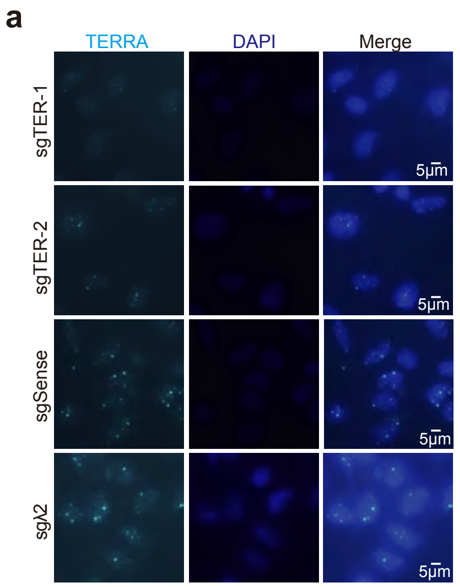

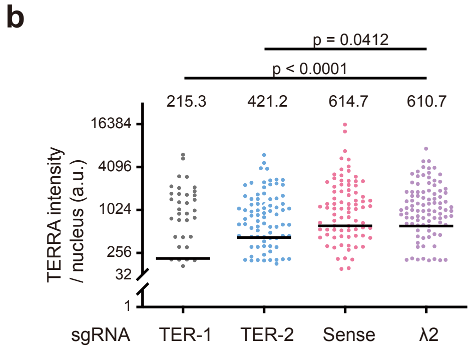

sgRNA	TERRA-1	TERRA-2	Sense	λ2
Total cell number	224	219	233	227
Cells with TERRA <100 a.u	187	133	143	126
% of cells with low TERRA intensity	83%	61%	61%	56%

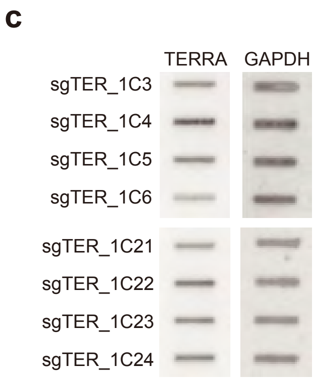

Supplementary Fig. 1. TERRA depletion by the RCas9 system. (Related to Fig. 1) a.TERRA RNA FISH in U2OS cells after transient transfection of RCas9-sgRNAs. Cells were transfected with RCas9-sgTERRA-1, RCas9-sgTERRA-2, RCas9-sgSense or RCas9-sgλ2 for 48 hr. **b.** Quantification of RNA FISH after transient transfection of RCas9-sgRNA to knockdown TERRA. The intensity of TERRA foci above 100 a.u. was selected and summed in each nucleus (upper panel). The intensity of TERRA foci below 100 a.u. was considered a low TERRA intensity (bottom table). One independent experiment. Bars, mean. P values by two-sided Mann-Whitney test. Mean values of each group shown on the top. **c.** RNA slot blotting shows the levels of TERRA RNA in RCas9-sgTERRA1 stable cell lines.

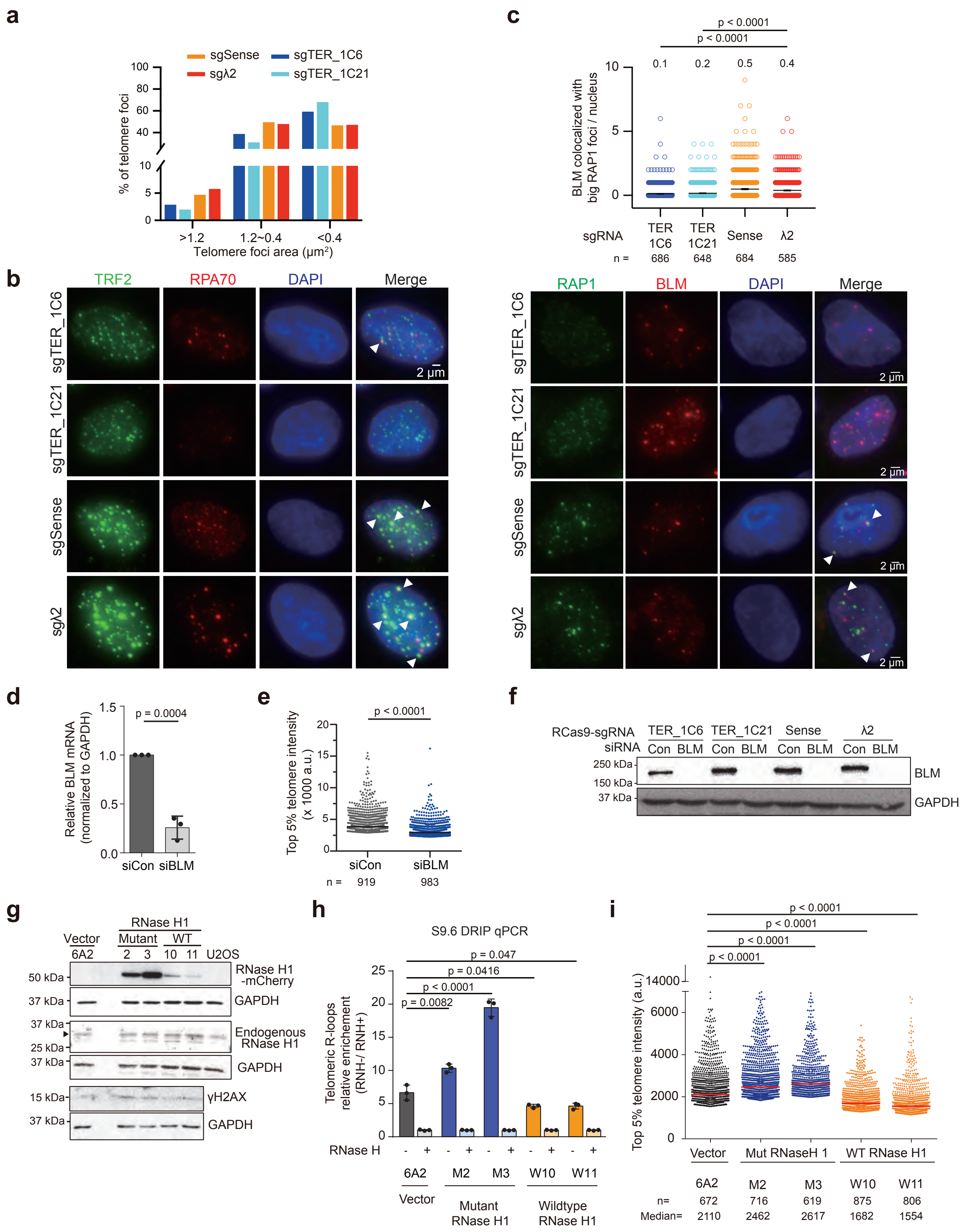

Supplementary Fig. 2. TERRA R-loops promote ALT features. (Related to Fig. 2) a. Histogram of the area of the telomere foci in stable cell lines expressing RCas9-sgRNAs. Representative of three independent experiments. **b.** Immunostaining of RPA70 and TRF2 in cells expressing RCas9-sgRNAs. The colocalization events of RPA foci with large TRF2 foci decrease in TERRA knockdown cells. Arrowheads indicate RPA70 with large TRF2 foci. **c.** Quantification of the colocalization events of BLM and big RAP1 (top 5%) foci in cells expressing RCas9-sgRNAs (top). Bars, mean± SEM. P values by two-sided Mann-Whitney test. n, cell number. Mean values of each group shown on the top. Data from three independent experiments. Representative images of immunostaining (bottom). **d.** Relative BLM RNA levels were determined by qRT-PCR after siRNA transfection for 3 days in U2OS cells. Data of three independent experiments. Bars, mean ± SEM. P values by two-tailed Student's t-test. **e.** Quantification of the top 5% telomere intensity in BLM knockdown U2OS cells. Bars, medians of top 5% telomere intensity. P values by two-sided Mann-Whitney test. Representative of three independent experiments. n, number of telomere foci. **f.** Western blot analysis shows BLM protein levels in RCas9-sgRNA cells transfected with BLM or control siRNAs for 3 days. Two independent experiments show similar results provided in the Source Data. **g.** Western blot analysis shows the expression of RNase H1-mCherry, endogenous RNase H1, and γH2AX in cells stably overexpressing wildtype (WT clones #10, #11), mutant (clones #2, #3) RNase H1-mCherry or vector alone (clone #6A2). **h.** DRIP-qPCR for telomeric R-loops in cells overexpressing wildtype or mutant RNase H1-mCherry. P values by two-tailed Student's t-test. Representative of three independent experiments. Other independent experiments show similar trends and are provided in the Source Data. **i.** Telomere clustering determined by top 5% telomere intensity decreases in cells overexpressing wildtype RNase H1 compared to cells expressing catalytic-dead mutant RNase H1. Bars, medians. P values by two-sided Mann-Whitney test. Representative of three independent experiments. Other independent experiments show similar trends and are provided in the Source Data. n, number of telomere foci.

a

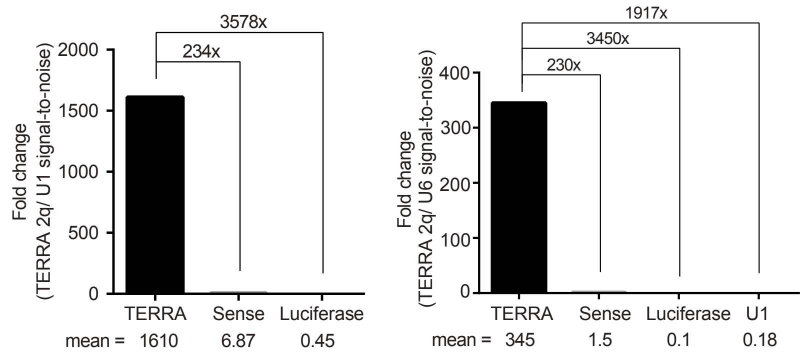

b

U1_iDRiP-MS: human ALT vs mES

U1-snRNP	Fold enrichment Human	Fold enrichment mES	Ranking Human	Ranking mES
snRNP70	24.7	30.5	2	5
U1 snRNP A	32.0	32.2	1	3
snRNP-B	23.4	18.1	4	11
U1 snRNP C	15.4	22.6	12	9
snRNP-E	16.7	29.0	10	7
snRNP-G	23.5	29.9	3	6
snRNP-F	2.87	4.4	233	33
Sm-D1	7.23	11.2	50	15
Sm-D2	15.1	1.7	13	262
Sm-D3	10.2	22.3	23	10

c

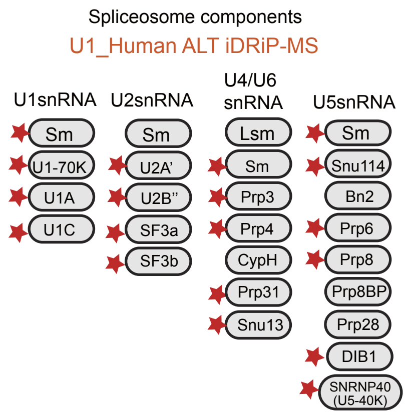

d

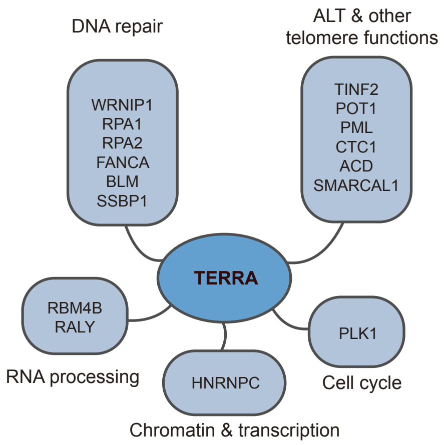

e

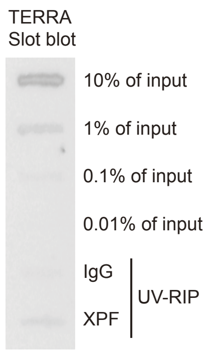

Supplementary Fig. 3. Comparison of TERRA iDRiP in mES cells and ALT cells. (Related to Fig. 3) a. Quantification of TERRA enrichment after iDRiP. qRT-PCR for TERRA transcripts derived from chromosome 2q, normalized with U1 RNA (left panel), or normalized with U6 RNA (right panel). Representative of four indepedent experiments. Other replicates show similar trends and are provided in the Source Data. **b.** Ranking of U1 interacting proteins revealed by U1-iDRiP in mES cells and human ALT cells (U2OS). **c.** Spliceosome components revealed by U1-iDRiP-MS in human ALT cells. Red stars mark proteins enriched in U1-iDRiP-MS in human ALT cells. **d.** Common TERRA interacting proteins revealed by TERRA-iDRiP-MS in mES cells and ALT cancer cells. **e.**TERRA RNA slot blotting shows the interaction of TERRA and XPF in U2OS cells after UV-RIP using XPF or IgG antibodies. Cell lysates were treated with DNase I prior to UV-RIP.

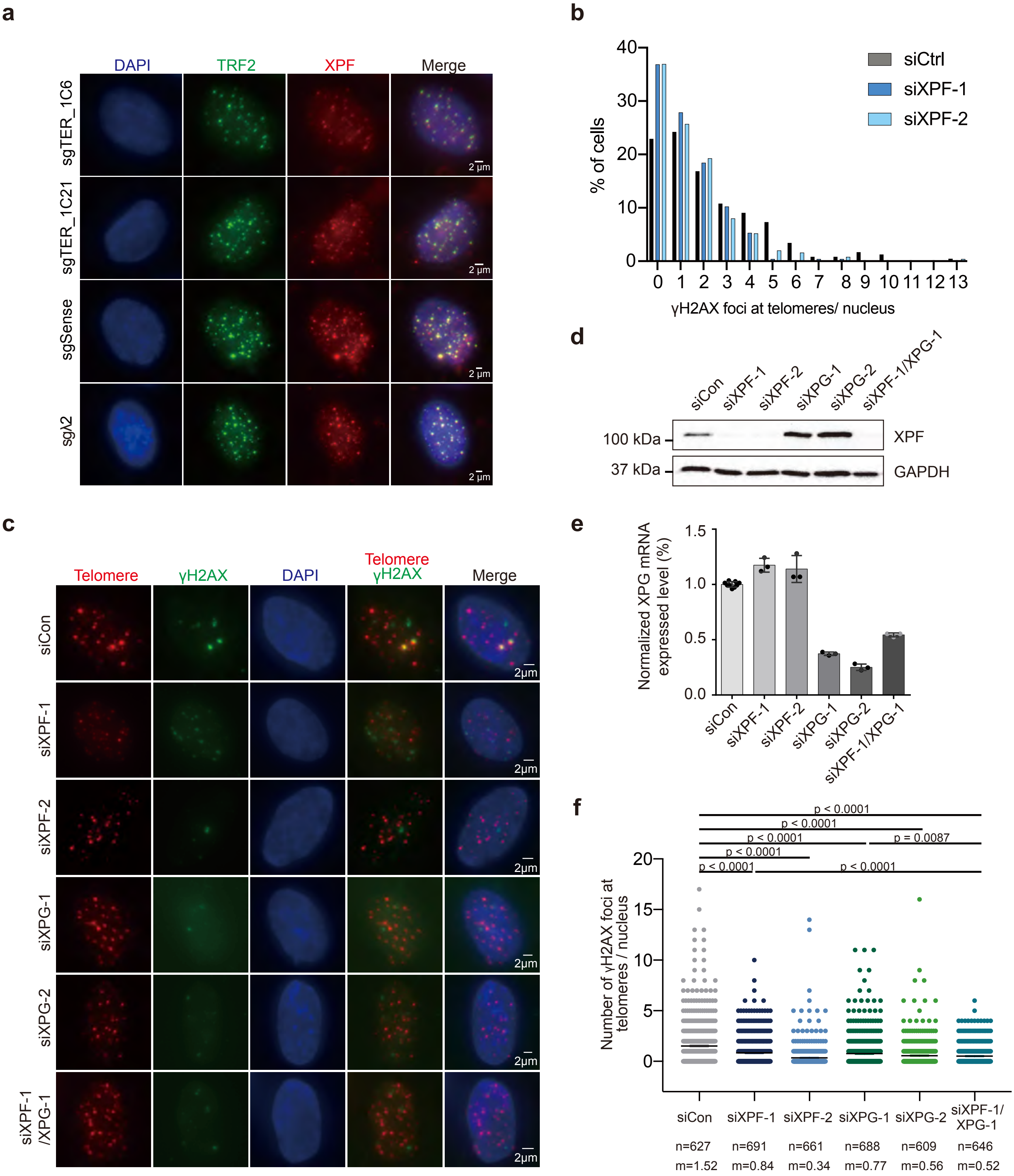

Supplementary Fig. 4. XPF and XPG are required for DNA damage response at ALT telomeres. (Related to Fig. 4) a. Representative images of XPF and TRF2 staining in TERRA knockdown cells (sgTER_1C6, sgTER_1C21) or control cells (sgSense, sgλ2). Three independent experiments show similar results. **b.** Histogram of U2OS cells containing γH2AX foci at telomeres after XPF knockdown. **c.** Representative images of immuno-DNA FISH to detect the colocalization of γH2AX and telomeres in U2OS cells after transfection with control, XPF, or XPG siRNAs. Cells were harvested at 72 hr after transfection. Three independent experiments show similar results. **d.** Western blot analysis for XPF after transfection with control, XPF, or XPG siRNAs. Two independent experiments show similar results. **e.** qRT-PCR analysis for XPG mRNA expression after knockdown of XPF and XPG in U2OS cells. Bars, mean ± SD. **f.** Quantification of γH2AX foci at telomeres per nucleus. Bars, mean ± SEM. P values by two-sided Mann-Whitney test. n, number of cells. m, mean value. Data from three independent experiments.

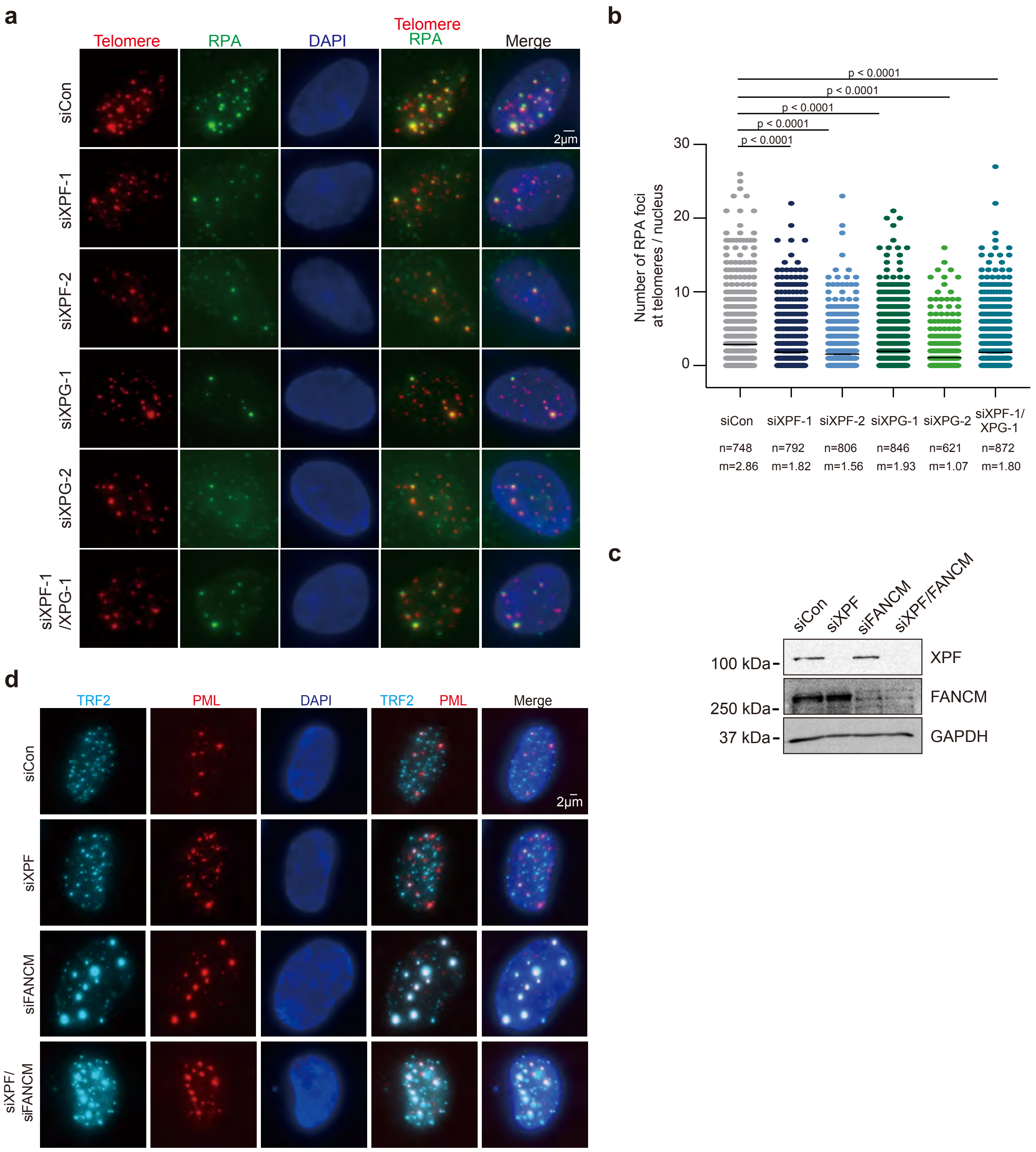

Supplementary Fig. 5. XPF is required for FANCM-deficiency induced DDR at ALT telomeres. (Related to Fig. 4 and Fig. 5) a. Representative images of immuno-DNA FISH to detect the colocalization of RPA70 and telomeres in U2OS cells after transfection with control, XPF, or XPG siRNAs. Cells were harvested at 72 hr after transfection. **b.** Quantification of RPA70 foci at telomeres per nucleus in (a). Bars, mean ± SEM. Data from three independent experiments. P values by two-sided Mann-Whitney test. n, number of cells. m, mean value. Data from three independent experiments. **c.** Western blot analysis to detect XPF and FANCM in U2OS cells after siRNA transfection. Three independent experiments show similar results and are provided in the Source Data. **d.** Representative images of immunostaining to detect the colocalization of PML and TRF2 for APBs. Cells were harvested at 72 hr after transfection with control, XPF, or FANCM siRNAs. Three independent experiments show similar results and are provided in the Source Data.

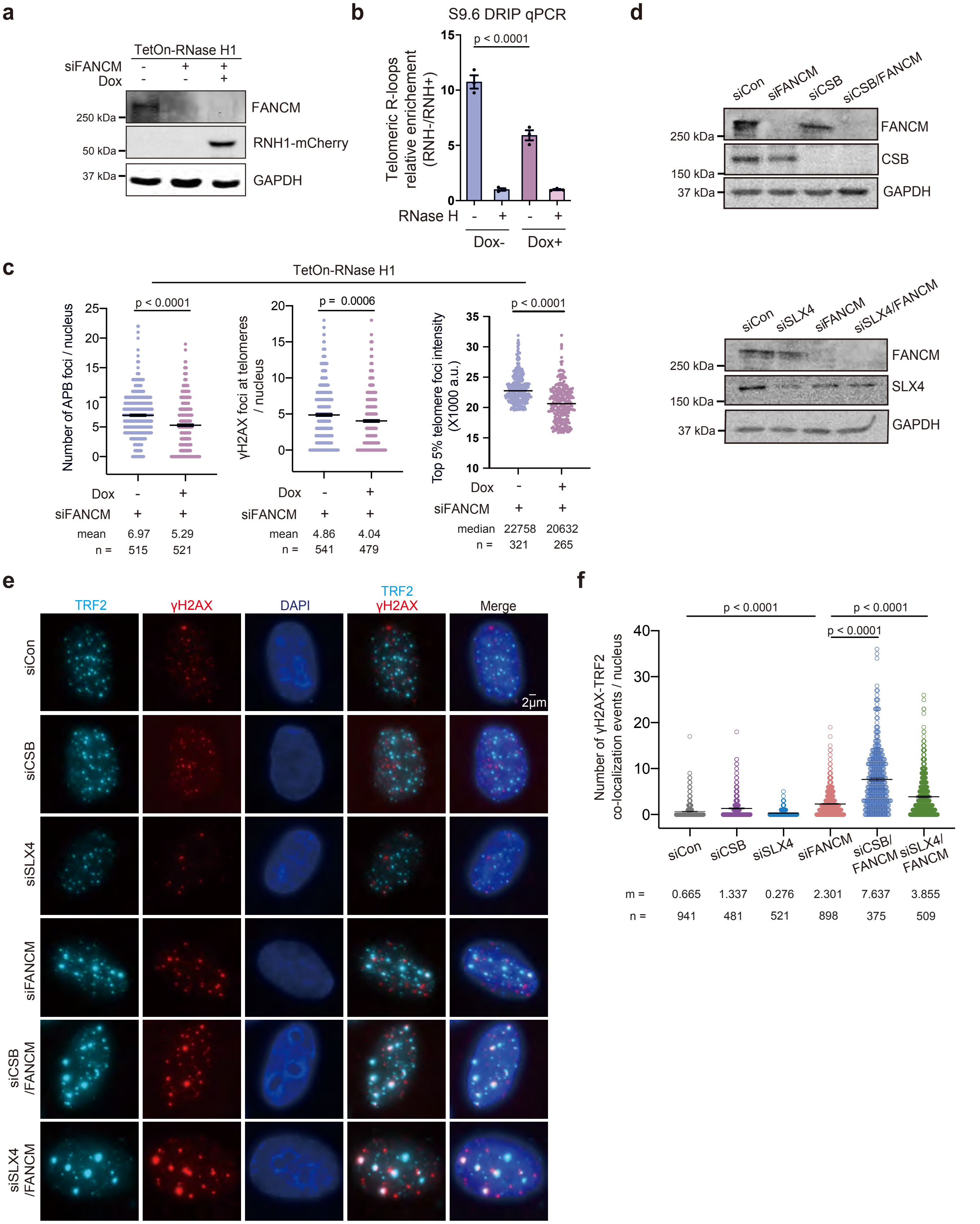

Supplementary Fig. 6. DDR at ALT telomeres induced by FANCM deficiency is mediated by XPF but not CSB or SLX4. (Related to Fig. 5) a. Western blot analysis to detect RNase H1-mCherry after doxycycline induction in FANCM deficient U2OS cells. **b.** DRIP-qPCR for telomeric R-loops in FANCM-deficient cells ectopically expressing RNase H1-mCherry. Relative R-loop levels were normalized to the pretreated RNase H controls. P values by two-tailed Student's t-test. Representative of three independent experiments. Dox, doxycycline. **c.** Quantification of the numbers of APB foci, γH2AX at telomeres, and top 5% telomere intensity in FANCM-deficient cells ectopically expressing RNase H1-mCherry. Bars, mean ± SEM for APB foci, γH2AX foci. Bars, medians for top 5% telomere intensity. P values by two-sided Mann-Whitney test. n, cell number. Data from three independent experiments. **d.** Western blot analysis for CSB, SLX4, and FANCM in U2OS cells after siRNA transfection. GAPDH as a loading control. Two independent experiments show similar results. **e.** Representative images of immunostaining to detect the colocalization of γH2AX and TRF2 after transfection with control, CSB, SLX4, or FANCM siRNAs. Cells were harvested at 72 hr after transfection. Three independent experiments show similar results. **f.** Quantification of the colocalization events of γH2AX and TRF2 in CSB, SLX4, FANCM single or double knockdown cells. Bars, mean ± SEM. P values by two-sided Mann-Whitney test. n, cell number. Data from three independent experiments.

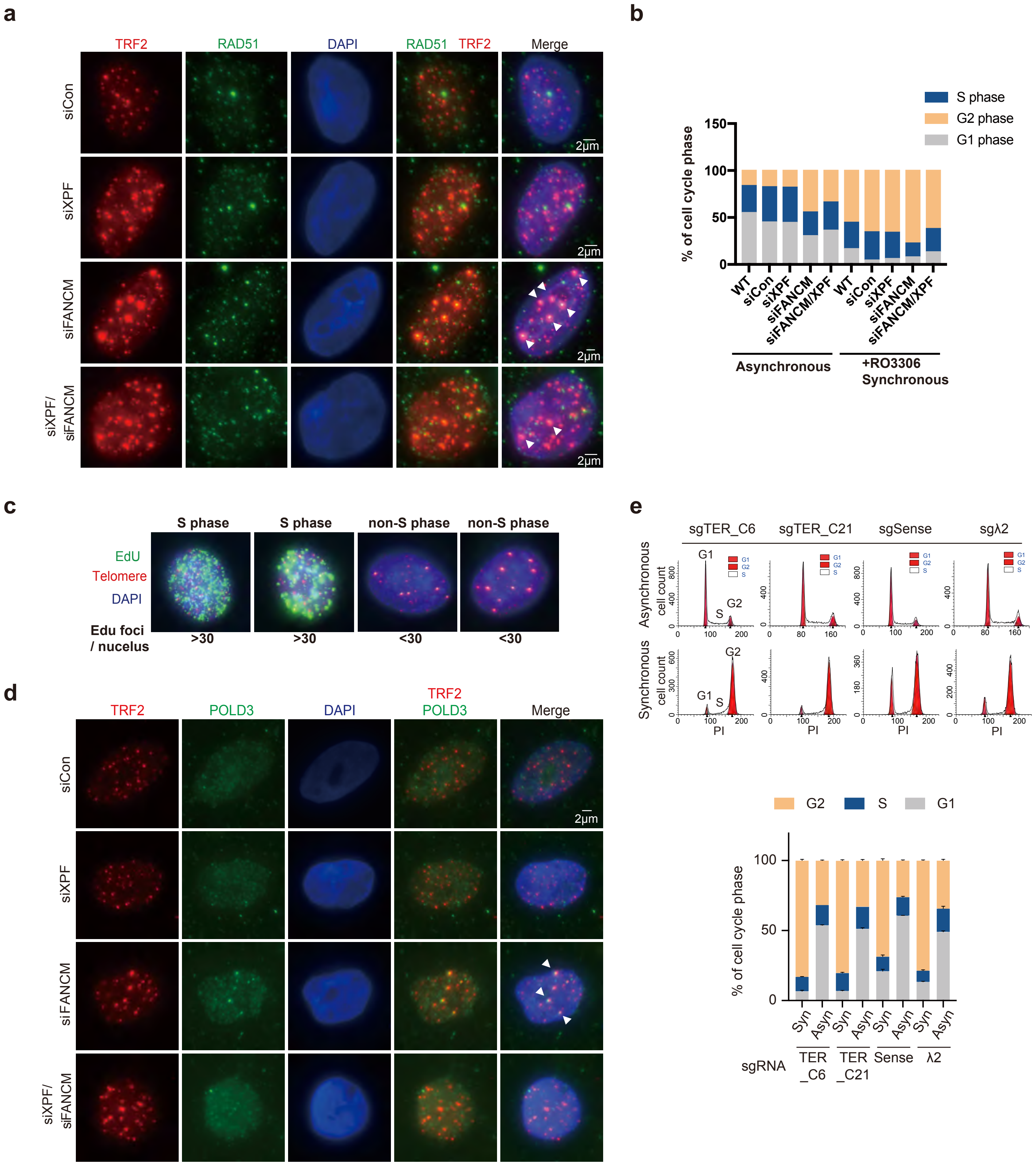

Supplementary Fig. 7. XPF promotes HR and break-induced telomere synthesis. (Related to Fig. 6) a. Representative images of immunostaining to detect the colocalization of RAD51 and TRF2 after transfection with control, XPF, or FANCM siRNAs. Cells were harvested at 72 hr after transfection. Arrowheads indicate colocalization events. Three independent experiments show similar results. **b.** Cell cycle profiles of XPF or XPF-FANCM knockdown cells after synchronization with RO-3306. Cells were stained with Propidium Iodide (PI) and analyzed by flow cytometry. The flow cytometry gating information was provided in Supplementary Data 2. Three independent experiments were averaged. **c.** Representative images of EdU-DNA-FISH to detect telomere synthesis in U2OS cells after the treatment of RO-3306. Non-S phase cells were determined by EdU foci < 30 per nucleus. Three independent experiments show similar results. **d.** Representative images of immunostaining to detect the colocalization of POLD3 and TRF2 after transfection with control, XPF, or FANCM siRNAs. Cells were harvested at 72 hr after transfection. Arrowheads indicate colocalization events. Three independent experiments show similar results. **e.** Cell cycle profiles of RCas9-sgRNA cells after synchronization with thymidine and RO-3306. Flow cytometry analyses of PI staining to detect DNA content (top). The flow cytometry gating information was provided in Supplementary Data 2. Bar graphs indicate the percentage of cells in G1, S, and G2 phases (bottom). Bars, mean ± SD. Three independent experiments were averaged.

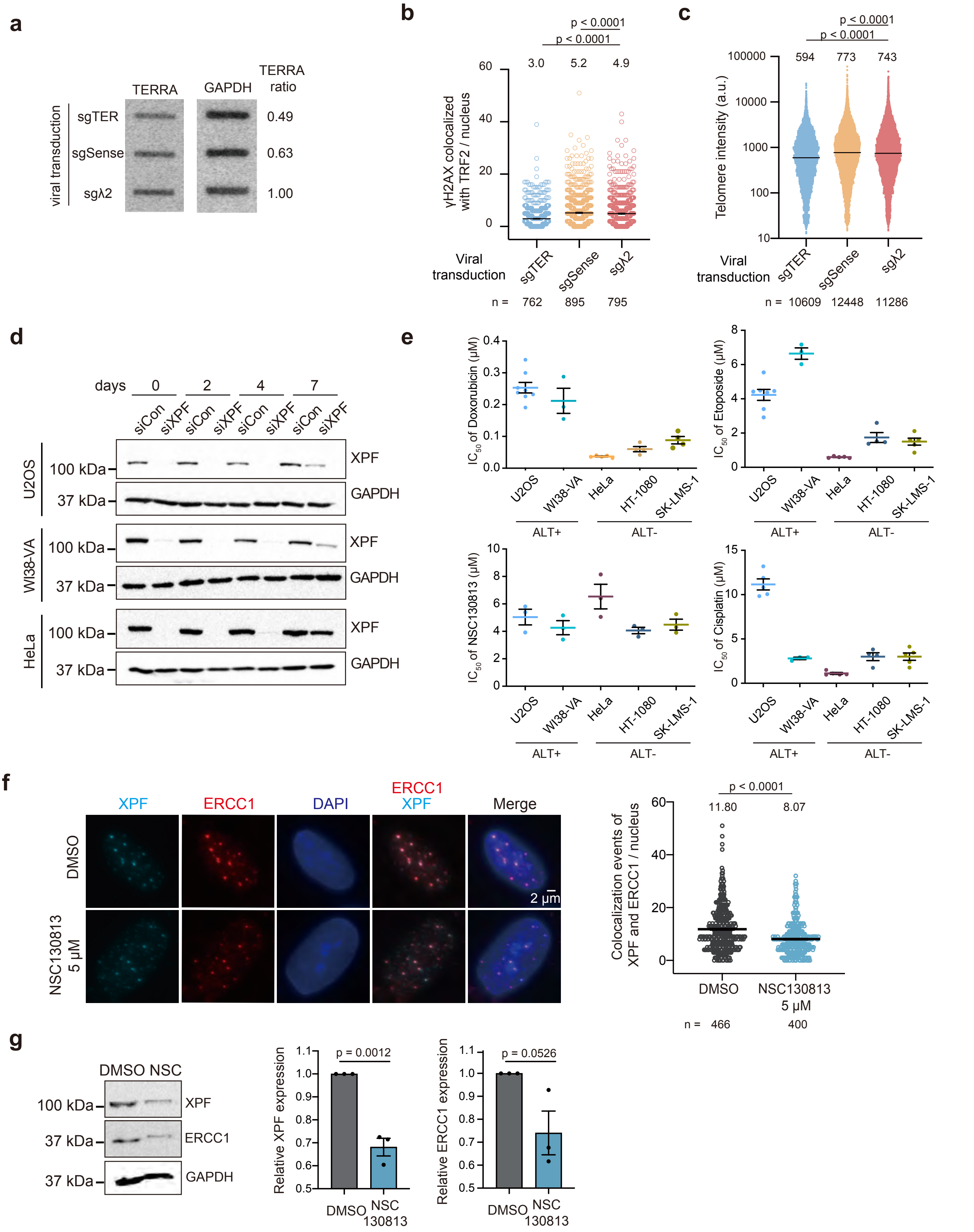

Supplementary Fig. 8. Inhibition of XPF suppresses cell proliferation in ALT cells. (Related to Fig. 7) a. RNA slot blotting shows the levels of TERRA RNA after viral transduction with RCas9-sgRNA vectors in U2OS cells. GAPDH as a loading control. TERRA levels were normalized to GAPDH. Three independent experiments show similar results. **b.** The colocalization events of γH2AX and TRF2 after viral transduction with RCas9-sgRNA vectors. n, cell number. Bars, mean ± SEM. P values by two-sided Mann-Whitney test. Data from three independent experiments. **c.** Telomere intensity after viral transduction with RCas9-sgRNA vectors. Bars, median. P values by two-sided Mann-Whitney test. Representative of three independent experiments. Other independent experiments show similar trends and are provided in the Source Data. n, number of telomere foci. **d.** Western blot analysis for XPF at post-transfection days after 3-day treatment of siRNA. siRNA was removed (day 0) and cells were plated for another 7 days for the cell proliferation assay. Two independent experiments show similar results and are provided in the Source Data. **e.** IC50 of indicated drugs (doxorubicin, etoposide, cisplatin, NSC130813) in ALT cells (U2OS and WI38-VA), and non-ALT cells (HeLa, HT-1080, and SK-LMS-1). Bars, mean ± SEM. Three to six independent experiments were averaged. **f.** Immunostaining of ERCC1 and XPF in U2OS cells treated with 5 μM NSC130813 or DMSO as a control (left). Quantification of the colocalization events of ERCC1-XPF per nucleus (right). n, cell number. Bars, mean ± SEM. P values by two-sided Mann-Whitney test. Data of three independent experiments. **g.** Western blotting to detect ERCC1 and XPF in U2OS cells treated with NSC130813 (left). Quantification of XPF and ERCC1 protein levels in U2OS cells treated with 5 μM NSC130813 (right). Three independent experiments were averaged. P value, by two-tailed Student's t-test.
