# Supplementary material for: XPF activates break-induced telomere synthesis
Source: Nat Commun. 2022 Oct 2;13:5781. doi: 10.1038/s41467-022-33428-0 (PMC9527253; doi:10.1038/s41467-022-33428-0)
Supplement: Supplementary file 3 — Description of additional Supplementary File [file 41467_2022_33428_MOESM3_ESM.pdf]

Descriptions of Additional Supplementary Data Files

Supplementary Data 1. Table of the information for antibodies, oligo sequences, siRNAs, cell lines, commercial assays and software used in the study.

Supplementary Data 2. Gating strategy for flow cytometry used in the cell cycle analysis.
