# Supplementary material for: XPF activates break-induced telomere synthesis
Source: Nat Commun. 2022 Oct 2;13:5781. doi: 10.1038/s41467-022-33428-0 (PMC9527253; doi:10.1038/s41467-022-33428-0)

Supplementary data 2[1]

1. Flow cytometry gating strategy

Cells without propidium iodide (PI) staining served as a negative control. Gating was based on comparison between side scatter (SSC) vs forward scatter (FSC) density plot by following order SSC-A vs FSC-A , FSC-W vs FSC-A, SSC-W vs SSC-A to remove debris. No antibodies are used in flow cytometry. The tube groups are described as below.

Tube_1: U2OS_WT_Asynchronized_without_PI
Tube_2: U2OS_WT_Synchronized_without_PI
Tube_3: U2OS_sgSense_ Synchronized_Rep1
Tube_4: U2OS_sgSense_ Synchronized_Rep2
Tube_5: U2OS_sgSense_ Synchronized_Rep3
Tube_6: U2OS_sgSense_ Asynchronized_Rep1
Tube_7: U2OS_sgSense_ Asynchronized_Rep2
Tube_8: U2OS_sgλ2_ Synchronized_Rep1
Tube_9: U2OS_sgλ2_ Synchronized_Rep2
Tube_10: U2OS_sgλ2_ Synchronized_Rep3
Tube_11: U2OS_sgλ2_ Asynchronized_Rep1
Tube_12: U2OS_sgλ2_ Asynchronized_Rep2
Tube_13: U2OS_sgTER_1C6_ Synchronized_Rep1
Tube_14: U2OS_sgTER_1C6_ Synchronized_Rep2
Tube_15: U2OS_sgTER_1C6_ Synchronized_Rep3
Tube_16: U2OS_sgTER_1C6_ Asynchronized_Rep1
Tube_17: U2OS_sgTER_1C6_ Asynchronized_Rep2
Tube_18: U2OS_sgTER_1C21_ Synchronized_Rep1
Tube_19: U2OS_sgTER_1C21_ Synchronized_Rep2
Tube_20: U2OS_sgTER_1C21_ Synchronized_Rep3
Tube_21: U2OS_sgTER_1C21_ Asynchronized_Rep1
Tube_22: U2OS_sgTER_1C21_ Asynchronized_Rep2
Tube_23: U2OS_sineg_ Synchronized_Rep1
Tube_24: U2OS_sineg_ Synchronized_Rep2
Tube_25: U2OS_sineg_ Asynchronized_Rep1
Tube_26: U2OS_sineg_ Asynchronized_Rep2
Tube_27: U2OS_siXPF_ Synchronized_Rep1
Tube_28: U2OS_siXPF_ Synchronized_Rep2
Tube_29: U2OS_siXPF_ Asynchronized_Rep1
Tube_30: U2OS_siXPF_ Asynchronized_Rep2

Tube_31: U2OS_siFANCM_ Synchronized_Rep1
Tube_32: U2OS_siFANCM_ Synchronized_Rep1
Tube_33: U2OS_siFANCM_ Asynchronized_Rep1
Tube_34: U2OS_siFANCM_ Asynchronized_Rep1
Tube_35: U2OS_siFANCM/XPF_ Synchronized_Rep1
Tube_36: U2OS_siFANCM/XPF_ Synchronized_Rep2
Tube_37: U2OS_siFANCM/XPF_ Asynchronized_Rep1
Tube_38: U2OS_siFANCM/XPF_ Asynchronized_Rep2
Tube_39: U2OS_WT_Synchronized_Rep1
Tube_40: U2OS_WT_Synchronized_Rep2
Tube_41: U2OS_WT_Asynchronized_Rep1
Tube_42: U2OS_WT_Synchronized_Rep2

BD FACSDiva 8.0.1

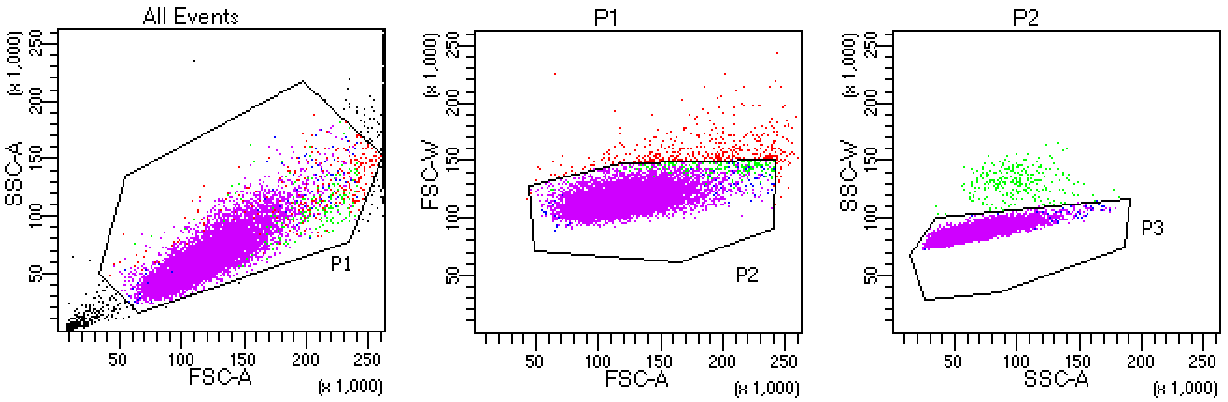

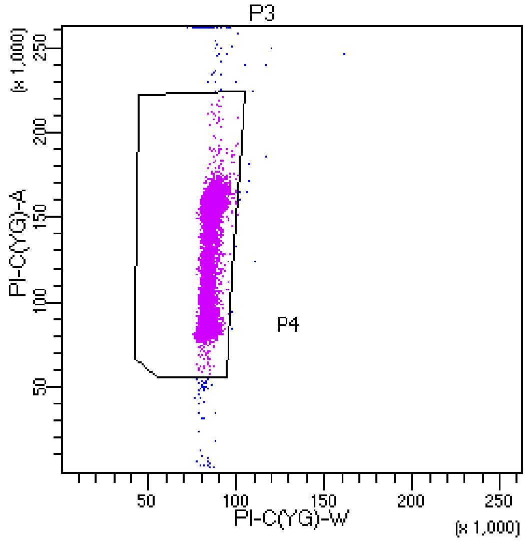

Tube: Tube_001

Population	#Events	%Parent	%Total
All Events	11,850	####	100.0
P1	11,053	93.3	93.3
P2	10,605	95.9	89.5
P3	10,327	97.4	87.1
P4	10,203	98.8	86.1
P5	0	0.0	0.0
P6	0	0.0	0.0

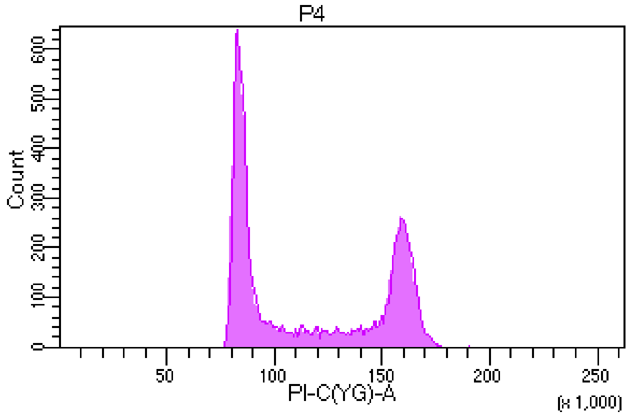

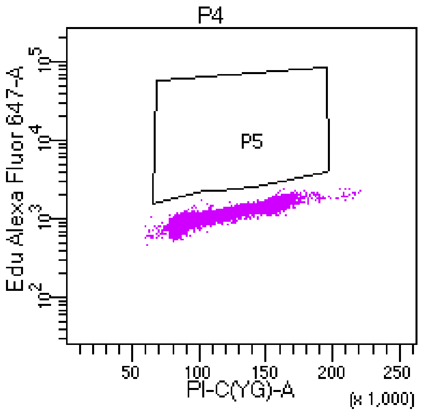

BD FACSDiva 8.0.1

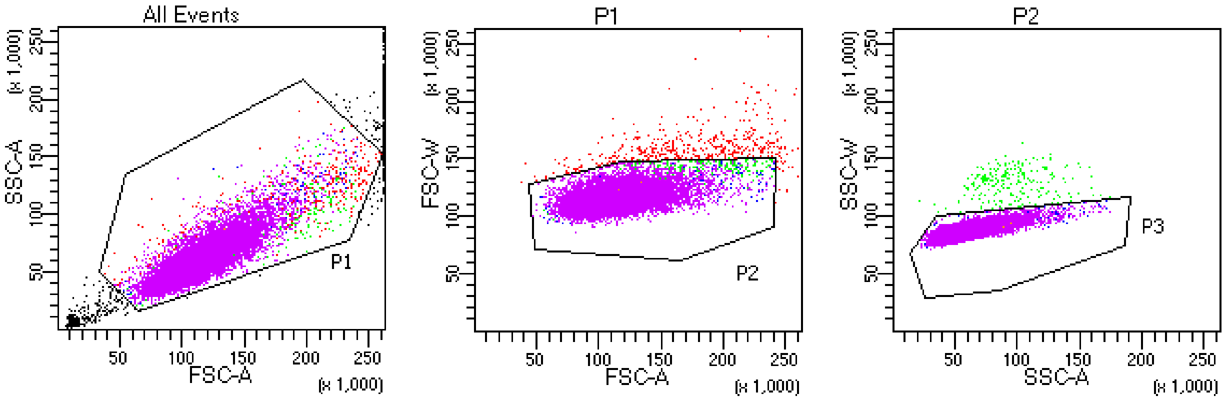

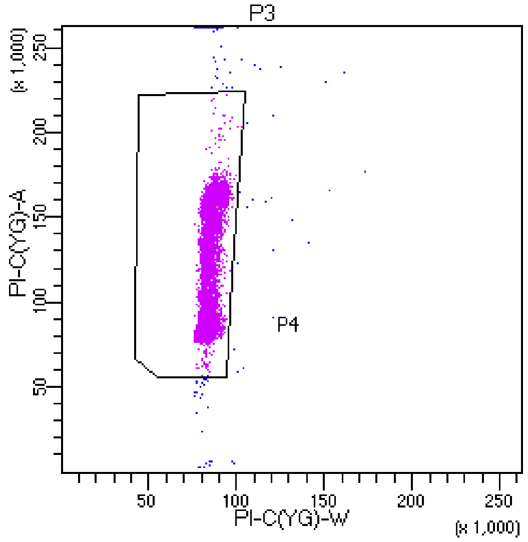

Tube: Tube_002

Population	#Events	%Parent	%Total
All Events	11,668	####	100.0
P1	11,006	94.3	94.3
P2	10,541	95.8	90.3
P3	10,304	97.8	88.3
P4	10,188	98.9	87.3
P5	2	0.0	0.0
P6	0	0.0	0.0

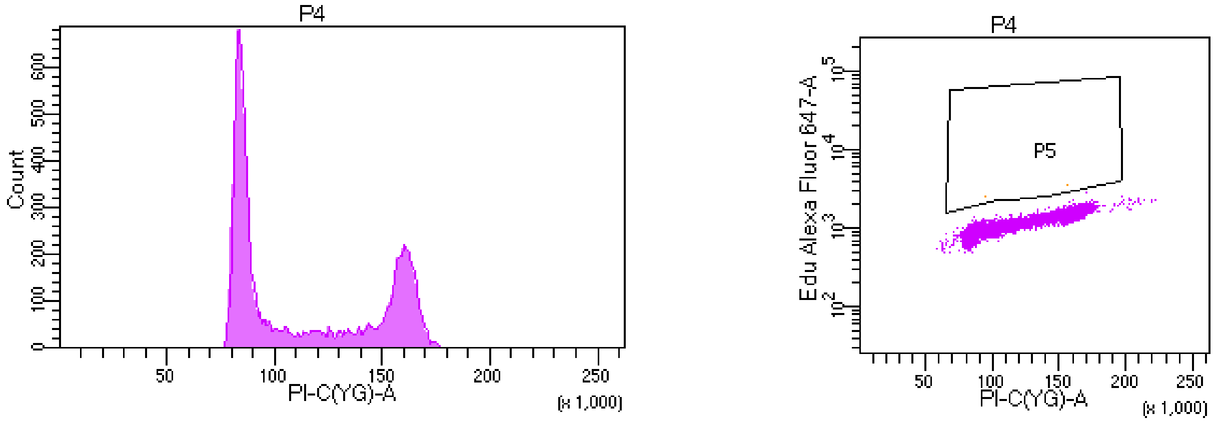

BD FACSDiva 8.0.1

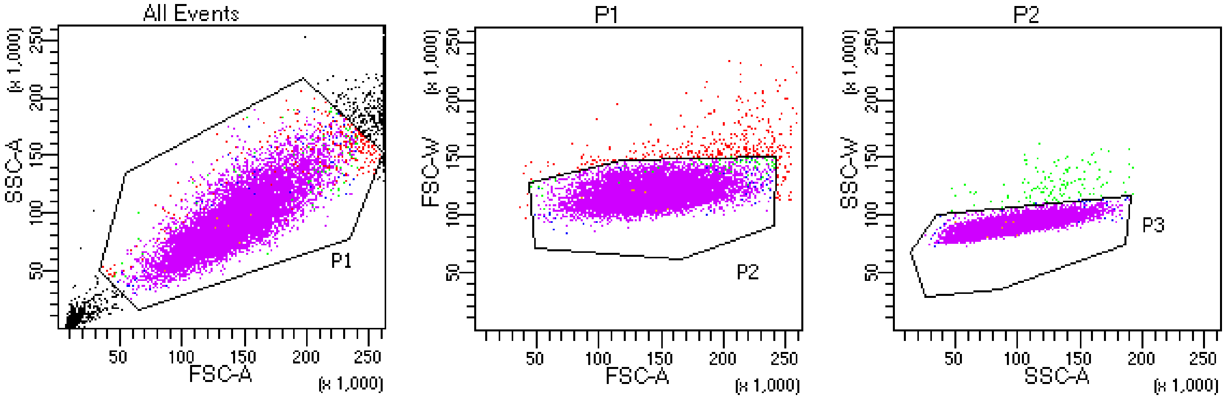

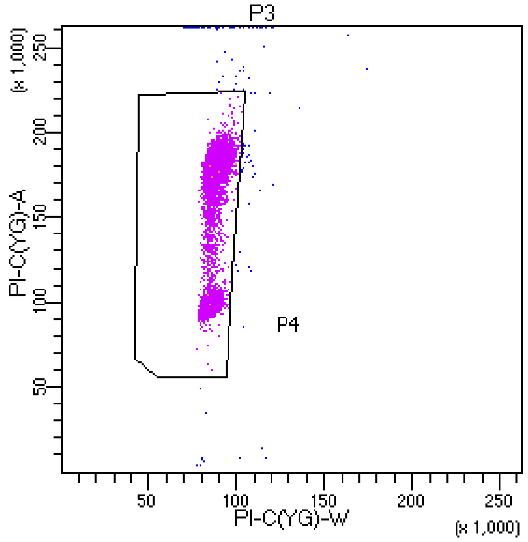

Tube: Tube_003

Population	#Events	%Parent	%Total
All Events	12,880	####	100.0
P1	10,951	85.0	85.0
P2	10,569	96.5	82.1
P3	10,438	98.8	81.0
P4	10,319	98.9	80.1
P5	6	0.1	0.0
P6	0	0.0	0.0

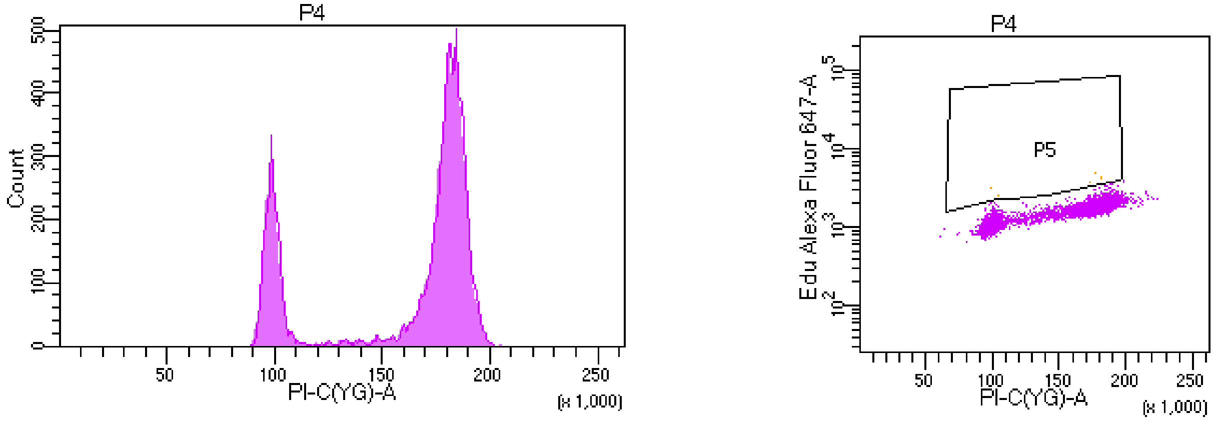

BD FACSDiva 8.0.1

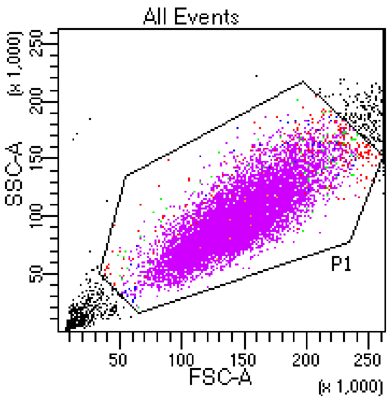

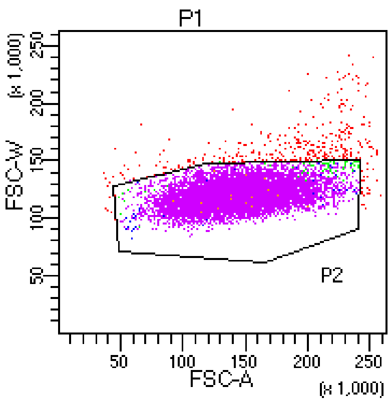

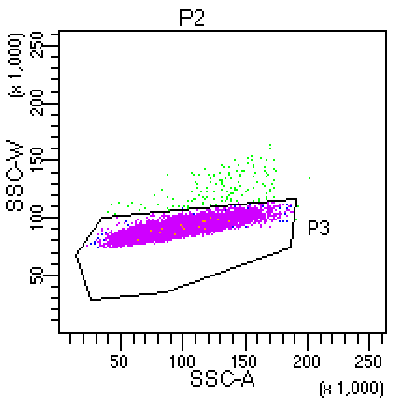

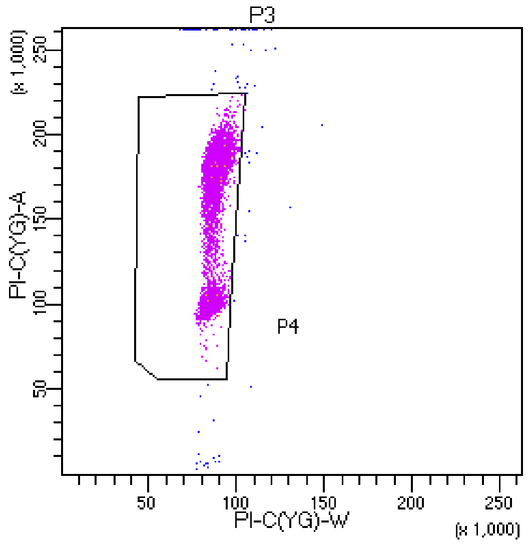

Tube: Tube_004

Population	#Events	%Parent	%Total
All Events	13,152	####	100.0
P1	10,967	83.4	83.4
P2	10,595	96.6	80.6
P3	10,455	98.7	79.5
P4	10,358	99.1	78.8
P5	15	0.1	0.1
P6	0	0.0	0.0

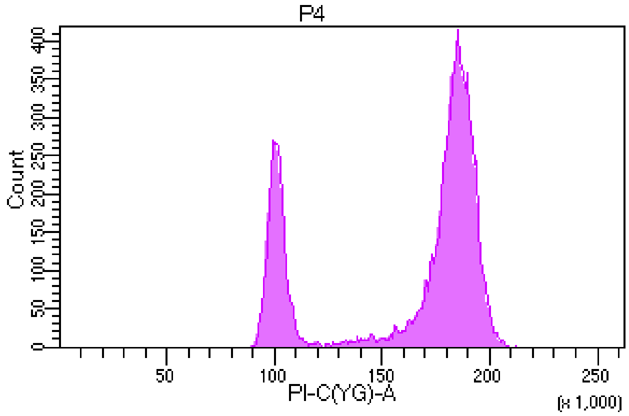

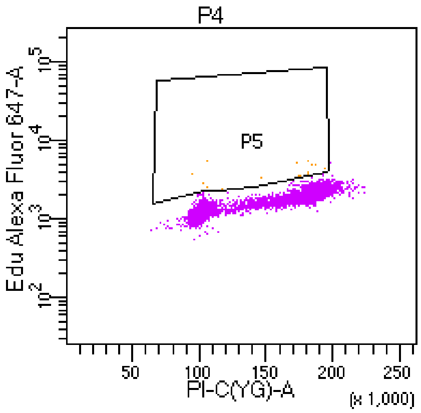

BD FACSDiva 8.0.1

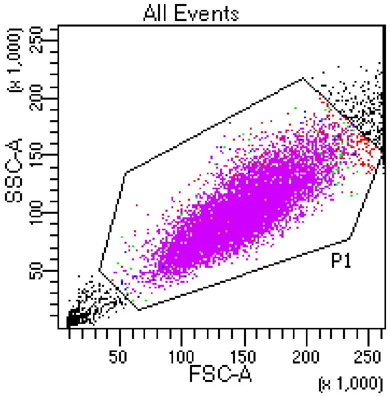

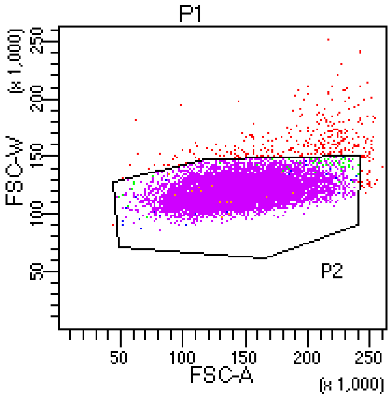

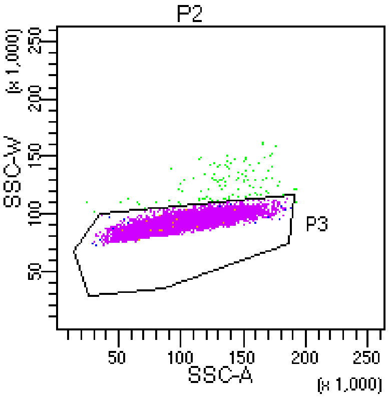

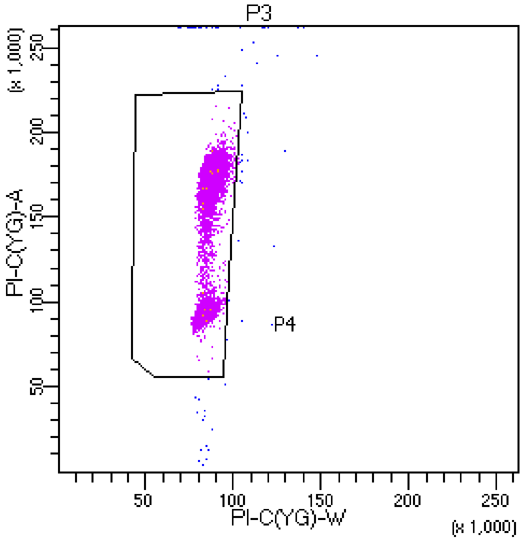

Tube: Tube_005

Population	#Events	%Parent	%Total
All Events	13,173	####	100.0
P1	10,899	82.7	82.7
P2	10,563	96.9	80.2
P3	10,453	99.0	79.4
P4	10,381	99.3	78.8
P5	14	0.1	0.1
P6	0	0.0	0.0

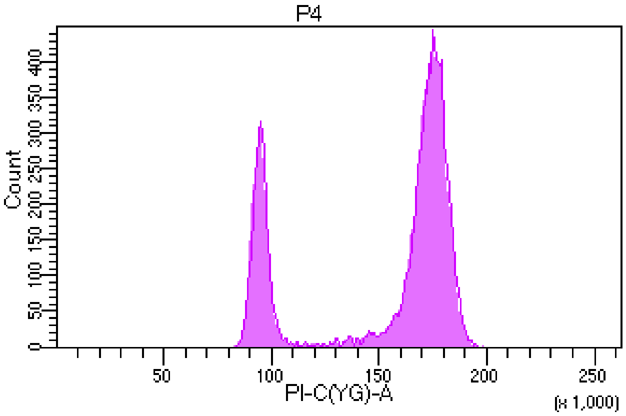

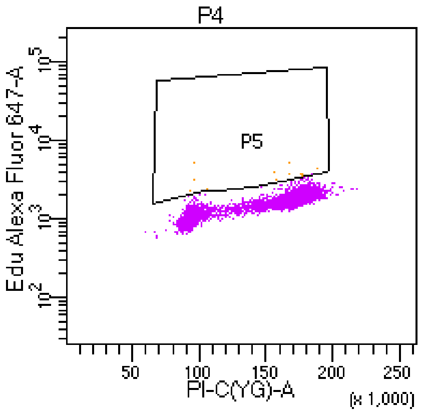

BD FACSDiva 8.0.1

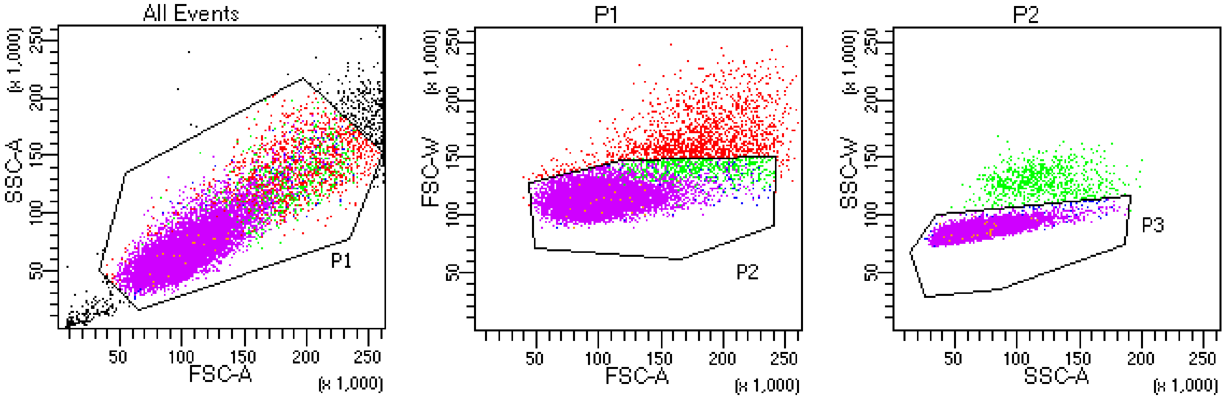

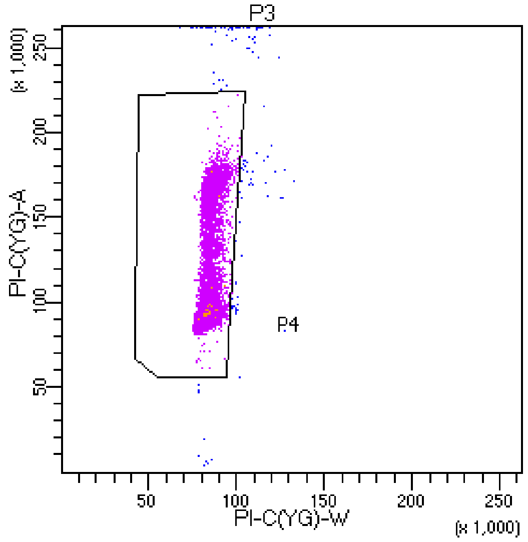

Tube: Tube_006

Population	#Events	%Parent	%Total
All Events	15,048	####	100.0
P1	12,758	84.8	84.8
P2	11,146	87.4	74.1
P3	10,372	93.1	68.9
P4	10,250	98.8	68.1
P5	22	0.2	0.1
P6	0	0.0	0.0

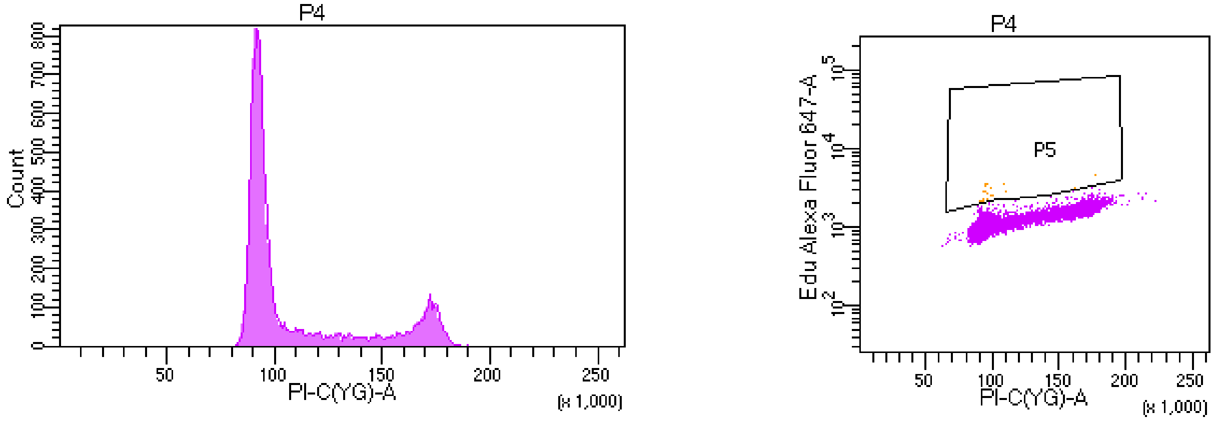

BD FACSDiva 8.0.1

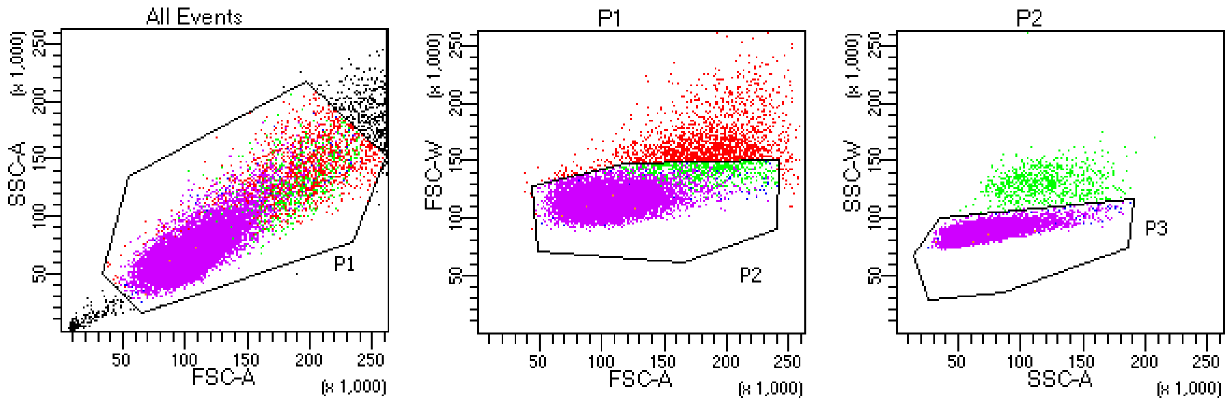

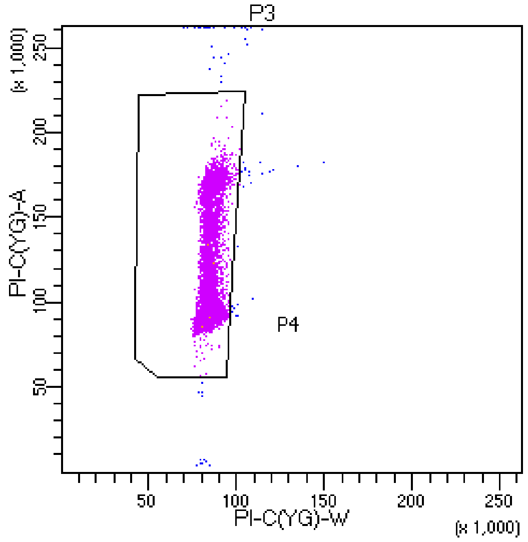

Tube: Tube_007

Population	#Events	%Parent	%Total
All Events	14,794	####	100.0
P1	12,745	86.1	86.1
P2	10,983	86.2	74.2
P3	10,239	93.2	69.2
P4	10,165	99.3	68.7
P5	4	0.0	0.0
P6	0	0.0	0.0

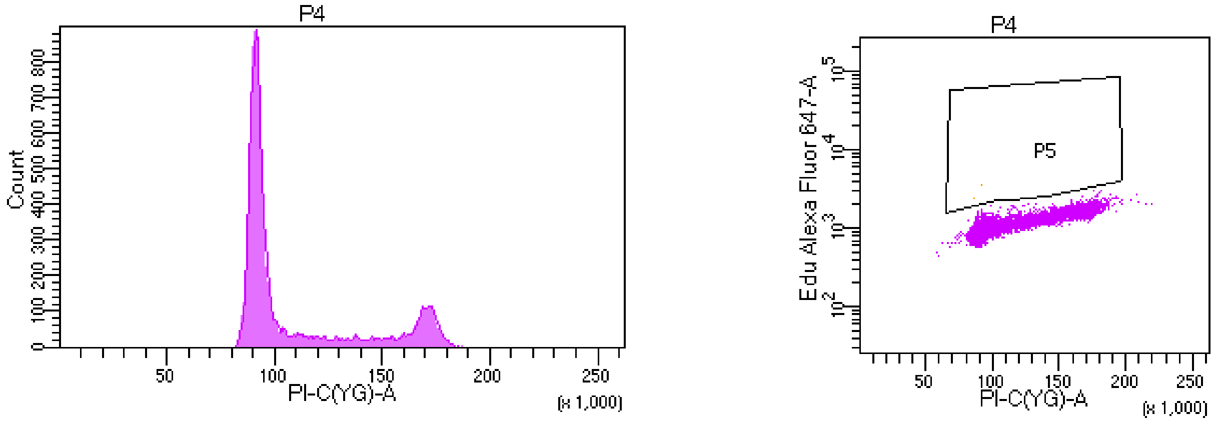

BD FACSDiva 8.0.1

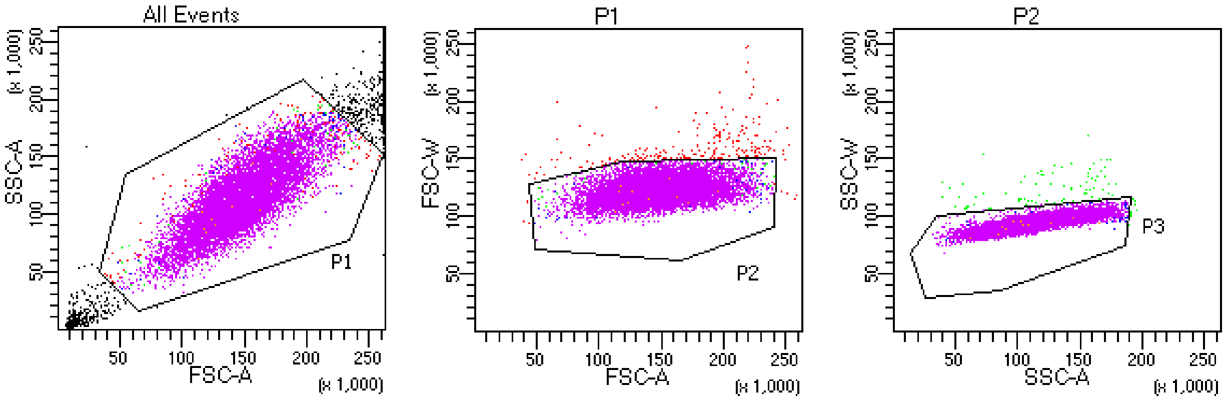

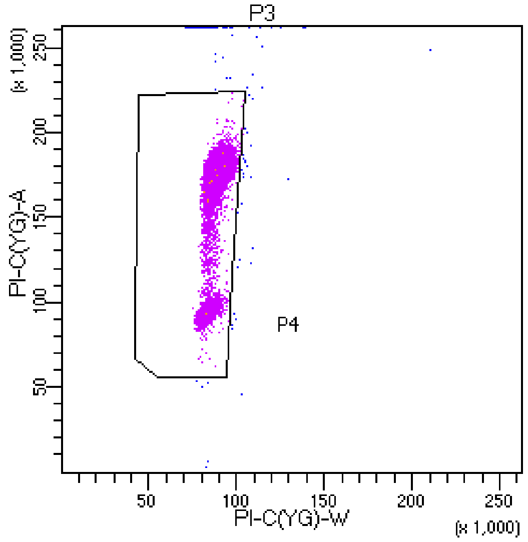

Tube: Tube_008

Population	#Events	%Parent	%Total
All Events	12,863	####	100.0
P1	10,982	85.4	85.4
P2	10,744	97.8	83.5
P3	10,662	99.2	82.9
P4	10,534	98.8	81.9
P5	11	0.1	0.1
P6	0	0.0	0.0

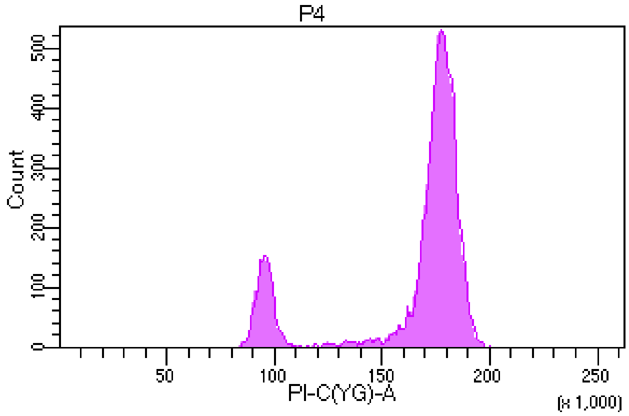

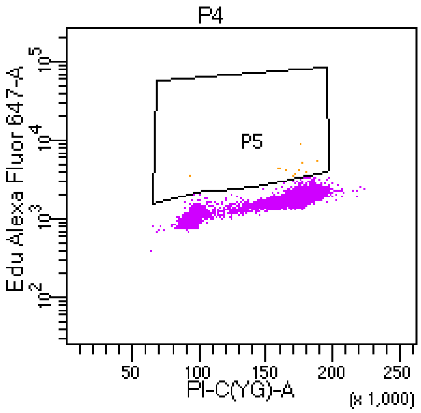

BD FACSDiva 8.0.1

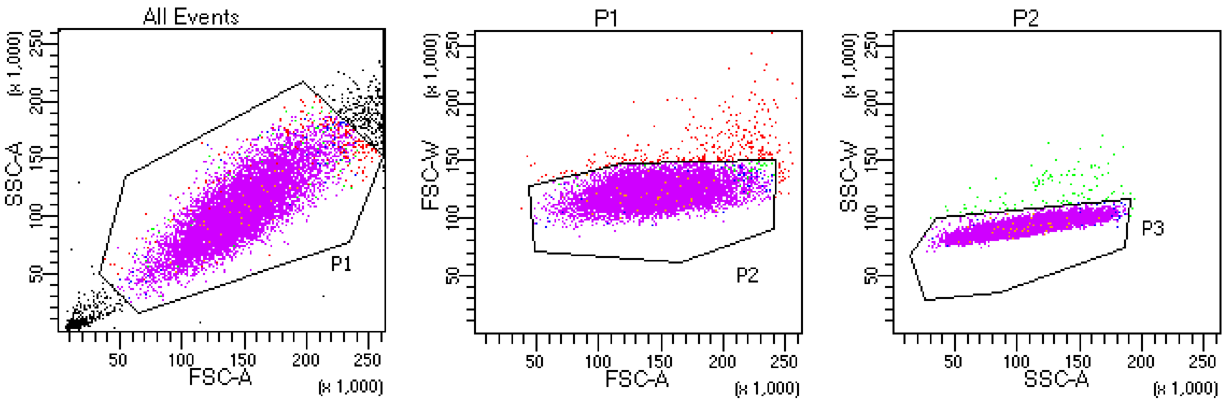

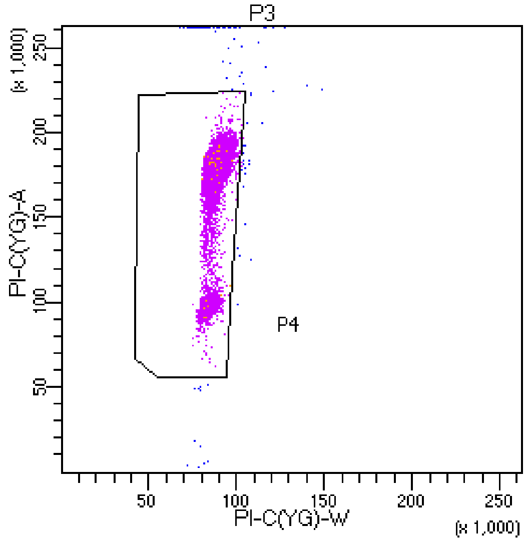

Tube: Tube_009

Population	#Events	%Parent	%Total
All Events	13,562	####	100.0
P1	10,920	80.5	80.5
P2	10,604	97.1	78.2
P3	10,498	99.0	77.4
P4	10,373	98.8	76.5
P5	36	0.3	0.3
P6	0	0.0	0.0

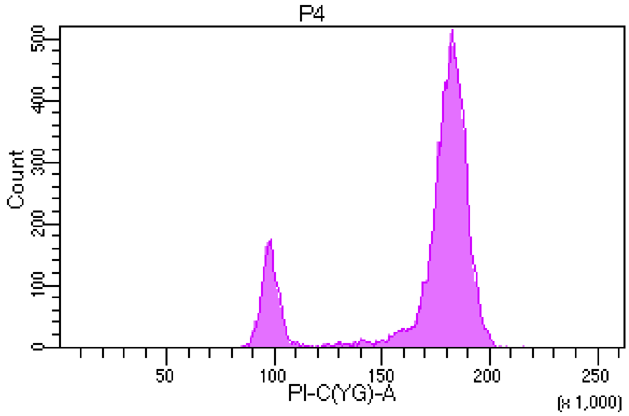

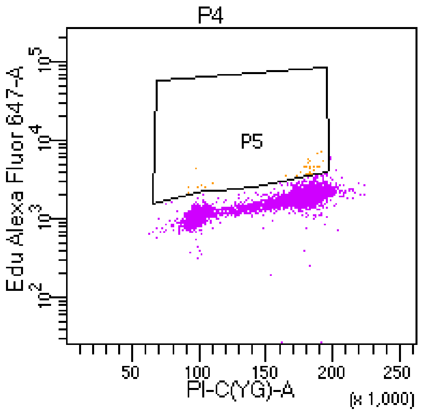

BD FACSDiva 8.0.1

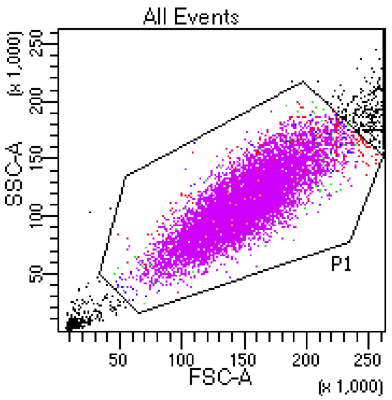

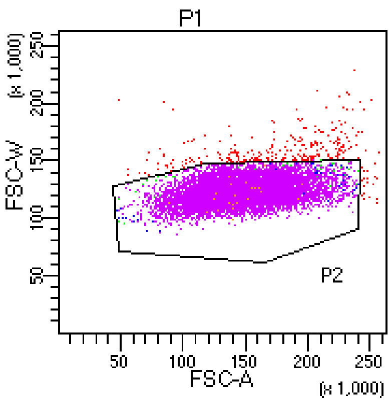

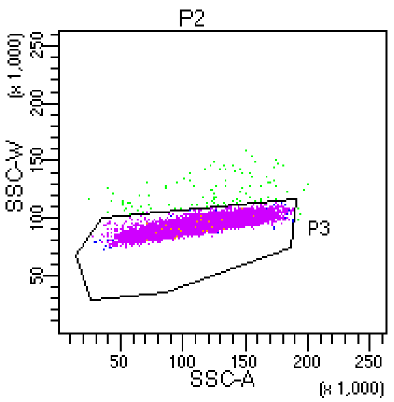

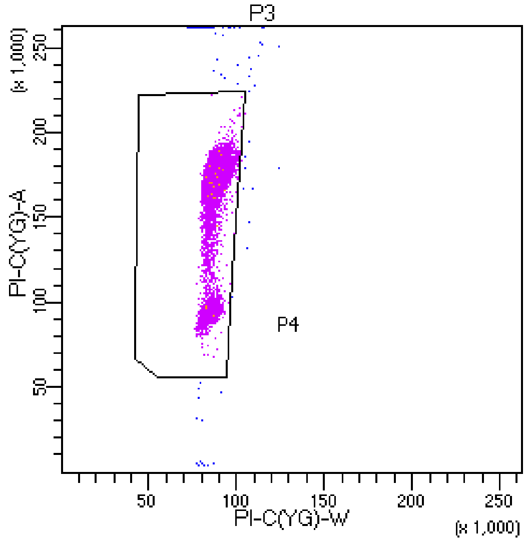

Tube: Tube_010

Population	#Events	%Parent	%Total
All Events	12,988	####	100.0
P1	10,919	84.1	84.1
P2	10,607	97.1	81.7
P3	10,501	99.0	80.9
P4	10,401	99.0	80.1
P5	22	0.2	0.2
P6	0	0.0	0.0

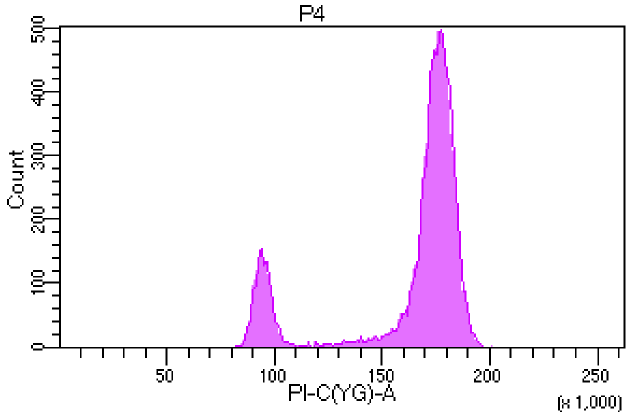

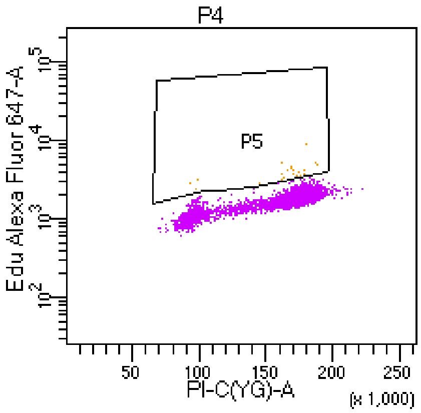

BD FACSDiva 8.0.1

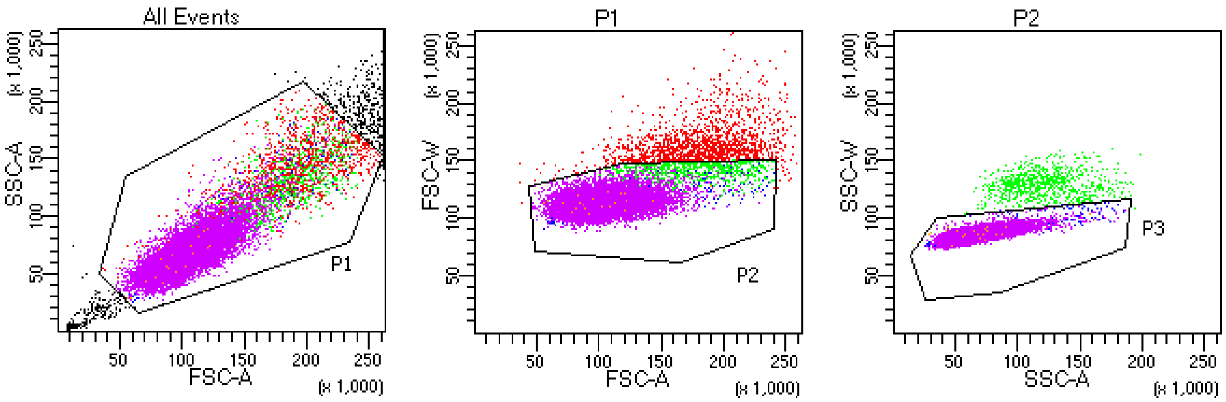

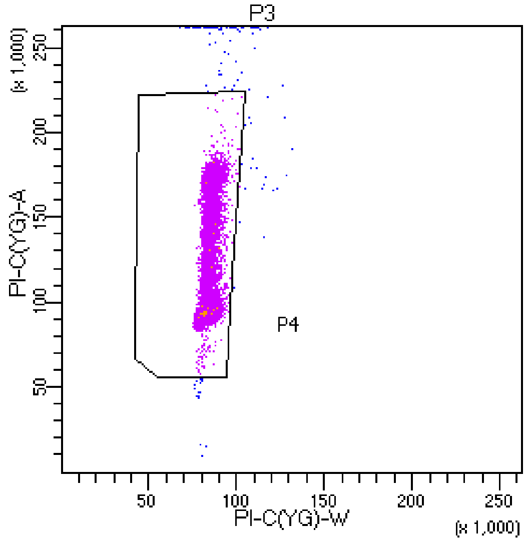

Tube: Tube_011

Population	#Events	%Parent	%Total
All Events	14,937	####	100.0
P1	12,620	84.5	84.5
P2	11,044	87.5	73.9
P3	10,221	92.5	68.4
P4	10,092	98.7	67.6
P5	21	0.2	0.1
P6	0	0.0	0.0

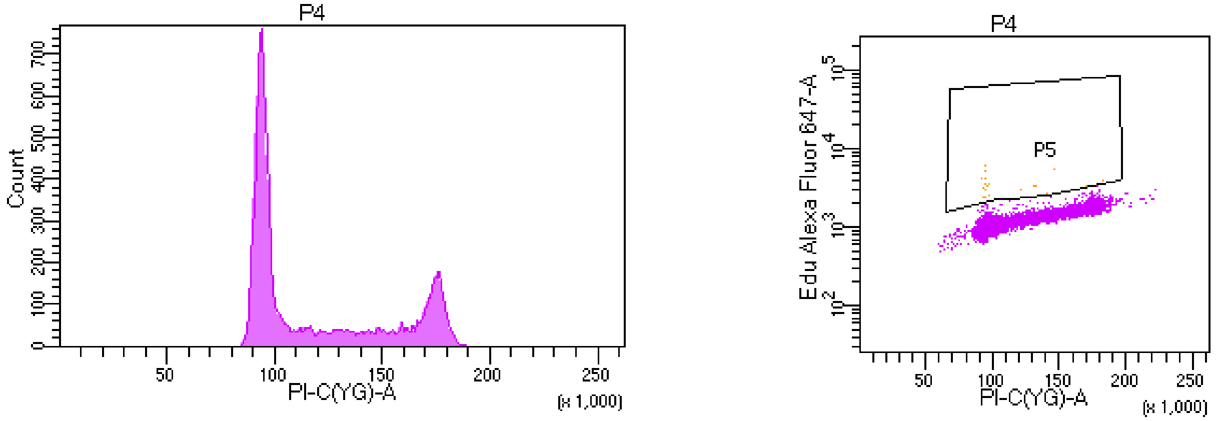

BD FACSDiva 8.0.1

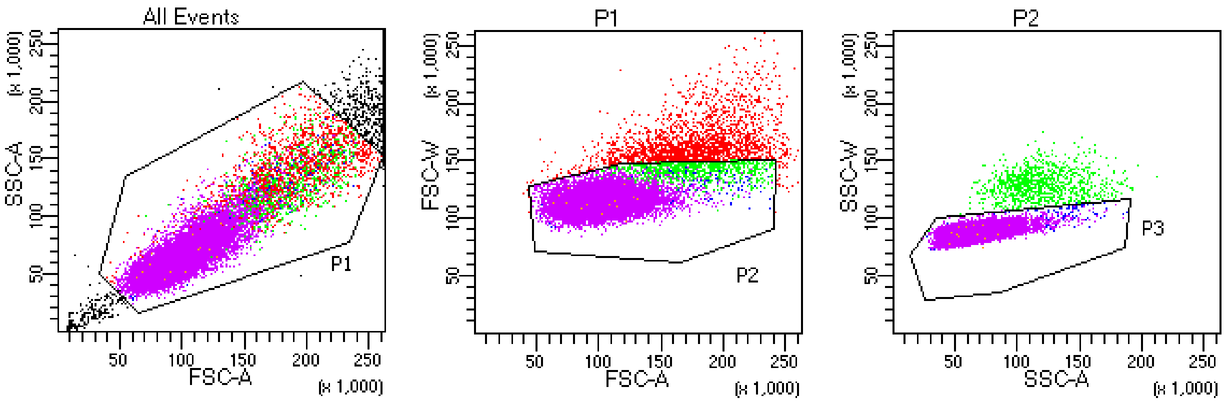

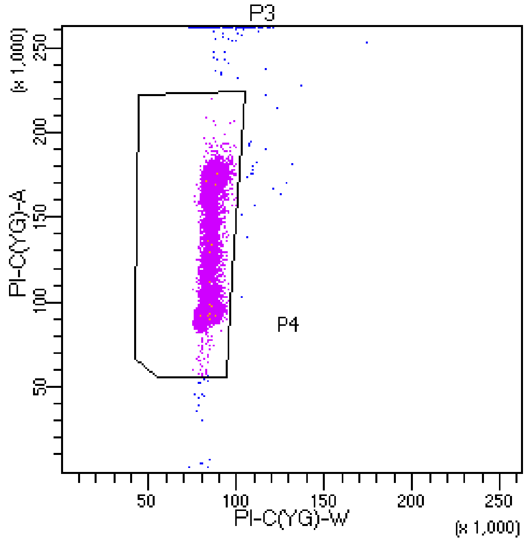

Tube: Tube_012

Population	#Events	%Parent	%Total
All Events	15,882	####	100.0
P1	13,437	84.6	84.6
P2	11,334	84.3	71.4
P3	10,308	90.9	64.9
P4	10,172	98.7	64.0
P5	14	0.1	0.1
P6	0	0.0	0.0

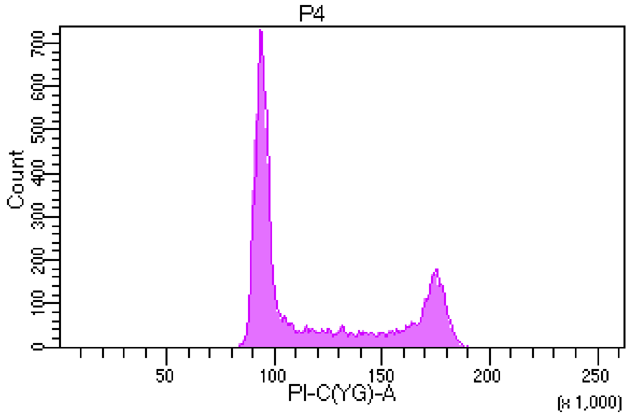

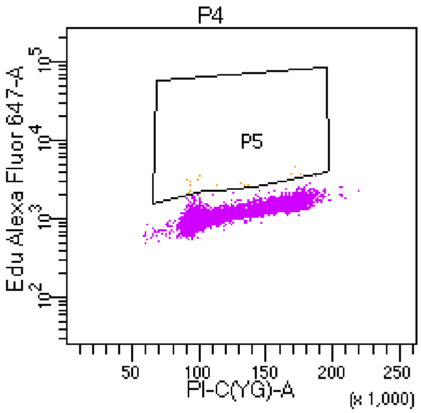

BD FACSDiva 8.0.1

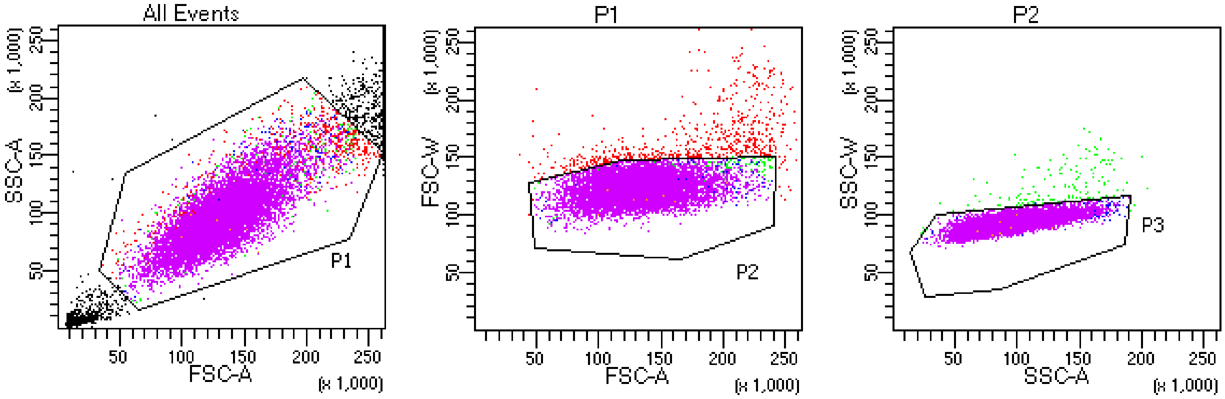

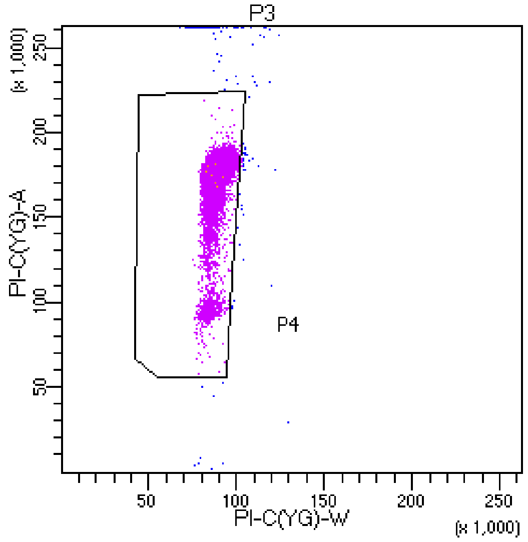

Tube: Tube_013

Population	#Events	%Parent	%Total
All Events	13,802	####	100.0
P1	11,209	81.2	81.2
P2	10,541	94.0	76.4
P3	10,391	98.6	75.3
P4	10,227	98.4	74.1
P5	7	0.1	0.1
P6	0	0.0	0.0

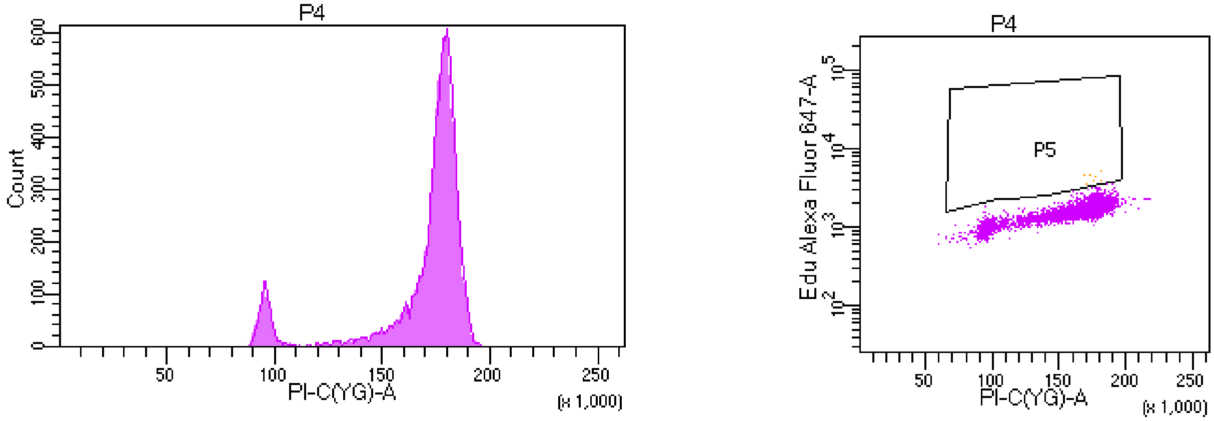

BD FACSDiva 8.0.1

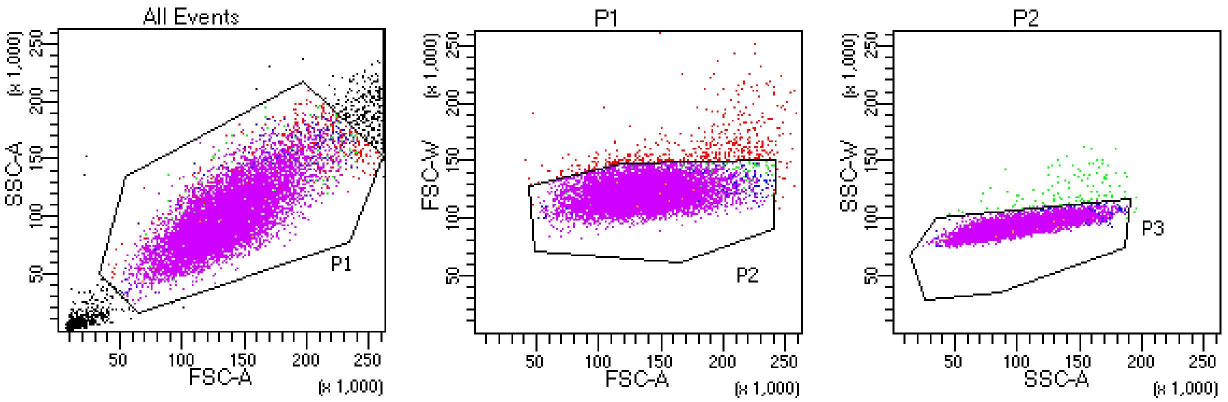

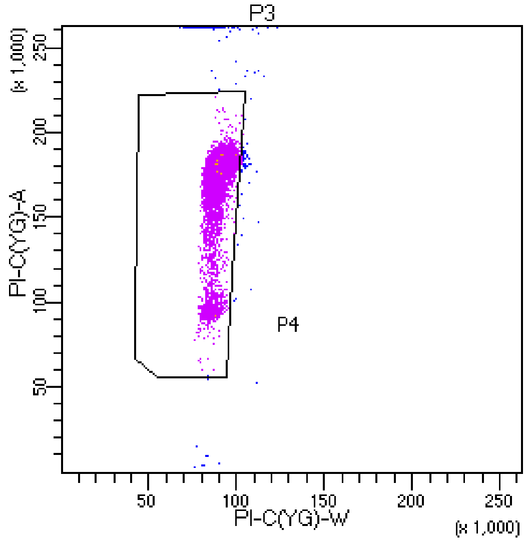

Tube: Tube_014

Population	#Events	%Parent	%Total
All Events	13,077	####	100.0
P1	11,077	84.7	84.7
P2	10,587	95.6	81.0
P3	10,476	99.0	80.1
P4	10,258	97.9	78.4
P5	9	0.1	0.1
P6	0	0.0	0.0

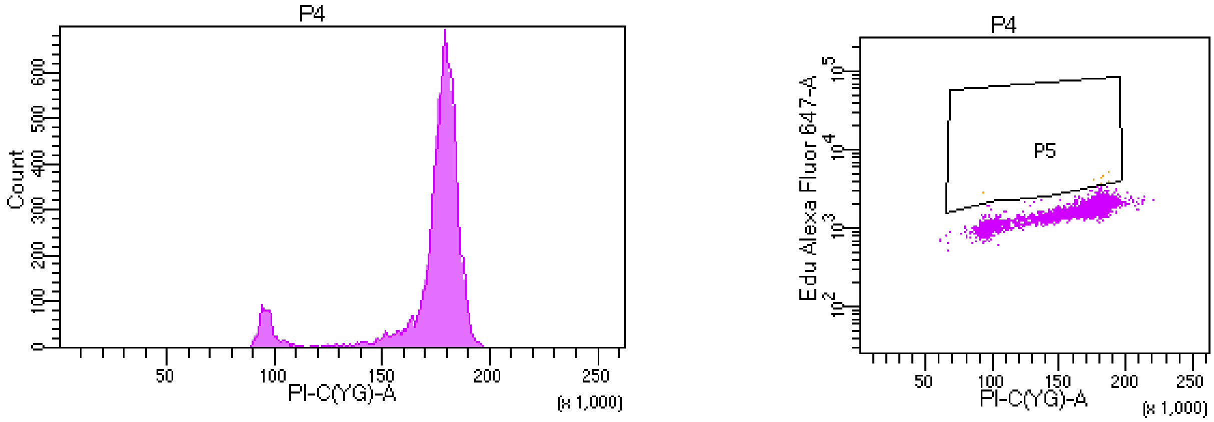

BD FACSDiva 8.0.1

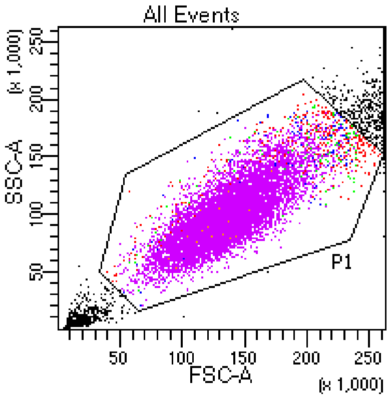

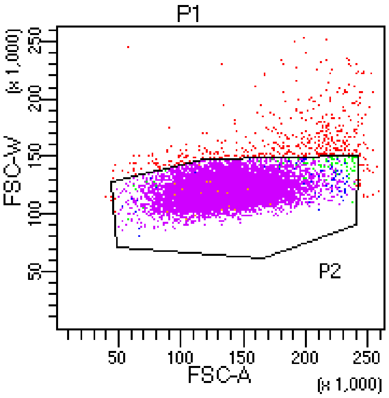

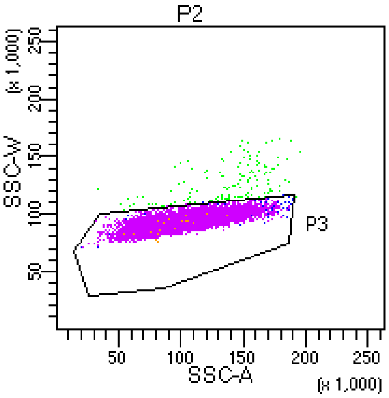

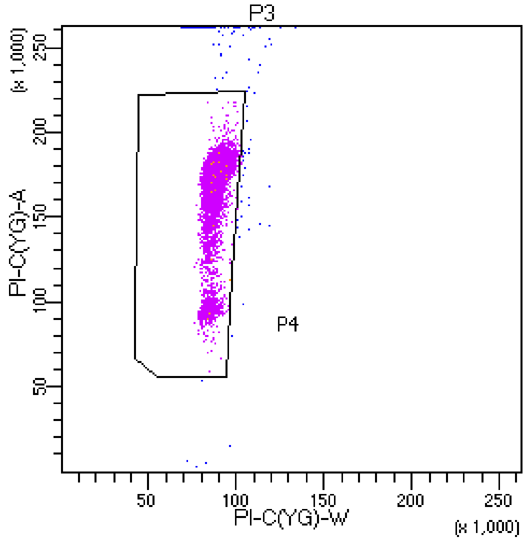

Tube: Tube_015

Population	#Events	%Parent	%Total
All Events	13,955	####	100.0
P1	11,017	78.9	78.9
P2	10,520	95.5	75.4
P3	10,374	98.6	74.3
P4	10,216	98.5	73.2
P5	18	0.2	0.1
P6	0	0.0	0.0

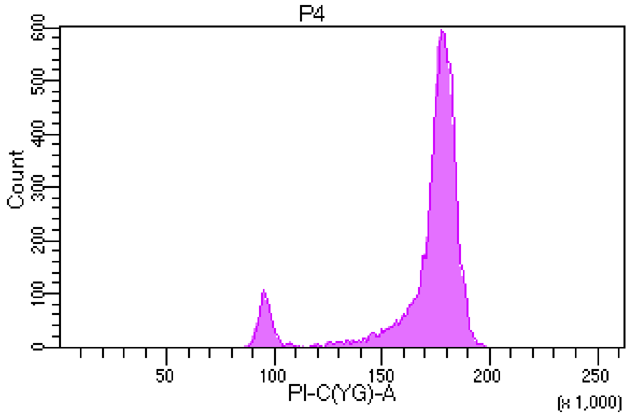

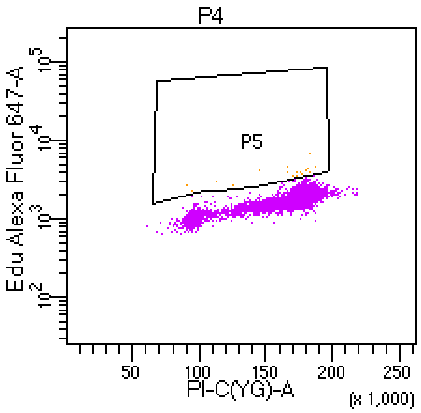

Global Sheet1

Printed on: Fri Dec 17, 2021 04:49:59 CST

BD FACSDiva 8.0.1

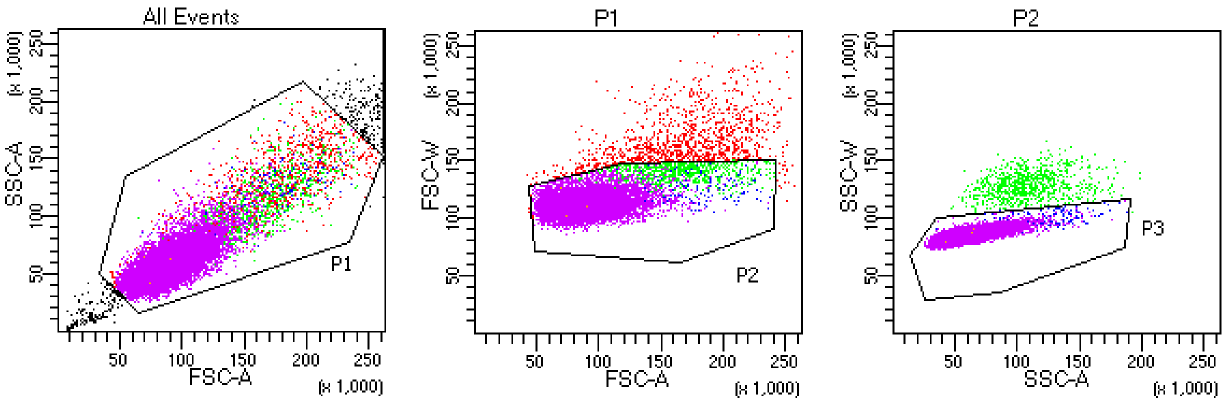

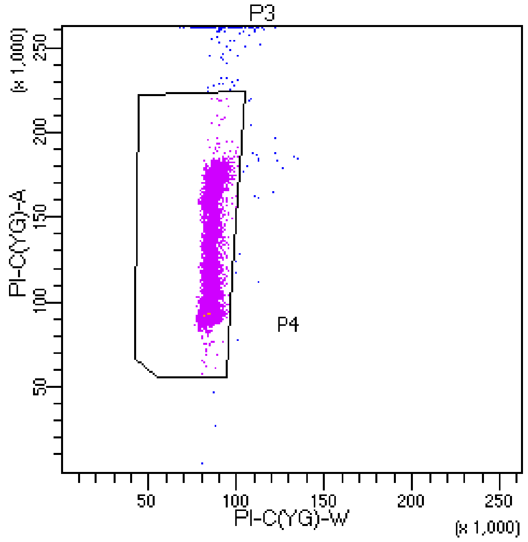

Tube: Tube_016

Population	#Events	%Parent	%Total
All Events	13,451	####	100.0
P1	12,345	91.8	91.8
P2	11,222	90.9	83.4
P3	10,382	92.5	77.2
P4	10,227	98.5	76.0
P5	3	0.0	0.0
P6	0	0.0	0.0

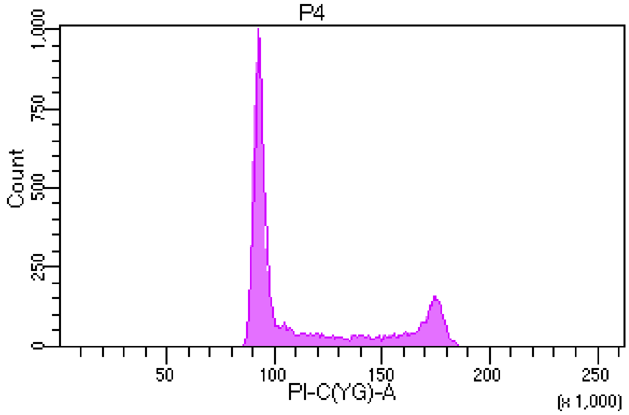

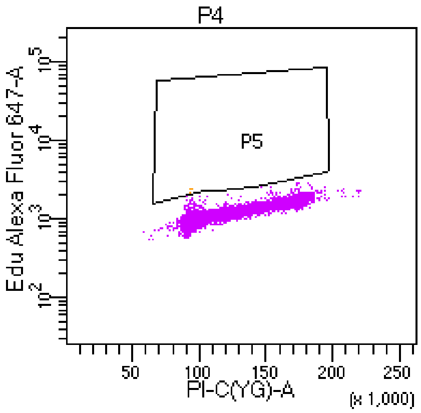

BD FACSDiva 8.0.1

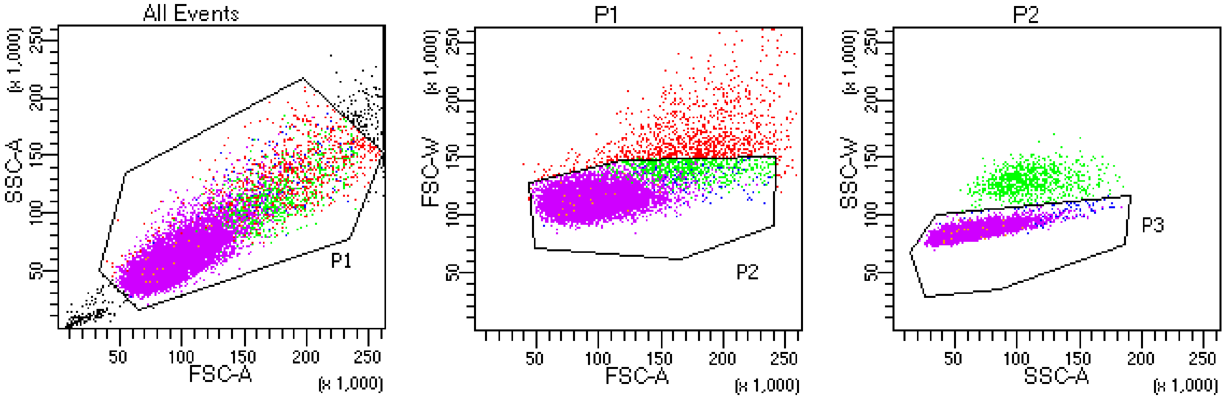

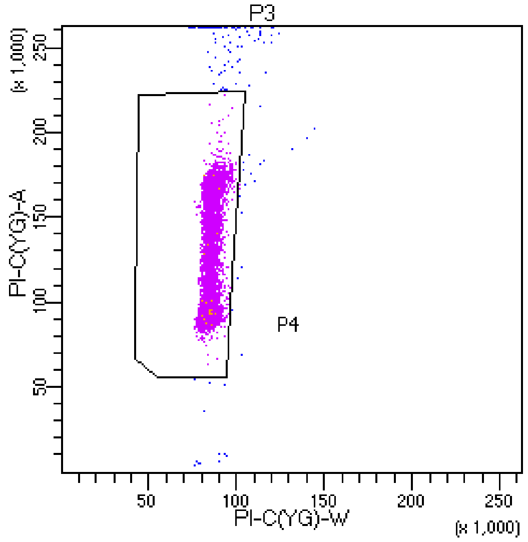

Tube: Tube_017

Population	#Events	%Parent	%Total
All Events	13,283	####	100.0
P1	12,096	91.1	91.1
P2	11,002	91.0	82.8
P3	10,148	92.2	76.4
P4	10,020	98.7	75.4
P5	19	0.2	0.1
P6	0	0.0	0.0

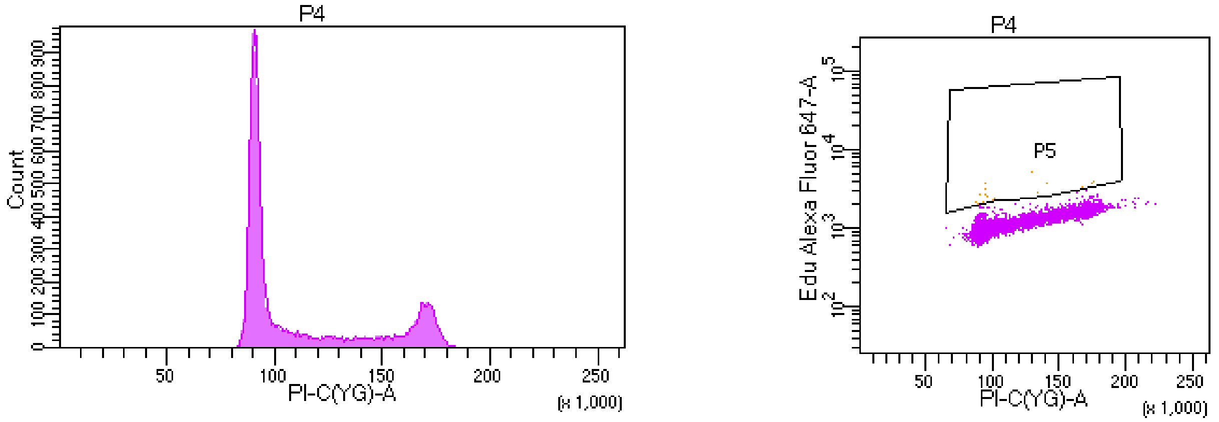

BD FACSDiva 8.0.1

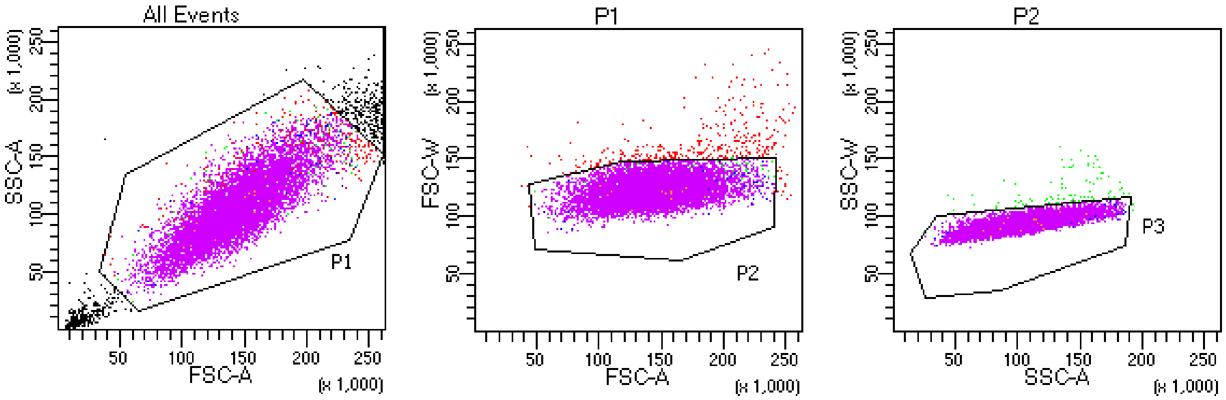

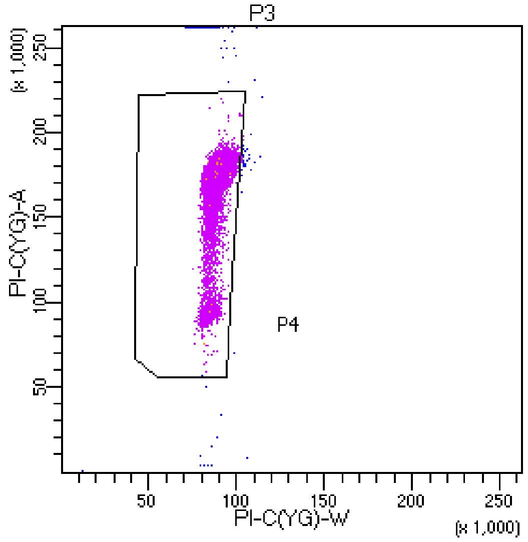

Tube: Tube_018

Population	#Events	%Parent	%Total
All Events	13,180	####	100.0
P1	10,864	82.4	82.4
P2	10,497	96.6	79.6
P3	10,410	99.2	79.0
P4	10,279	98.7	78.0
P5	14	0.1	0.1
P6	0	0.0	0.0

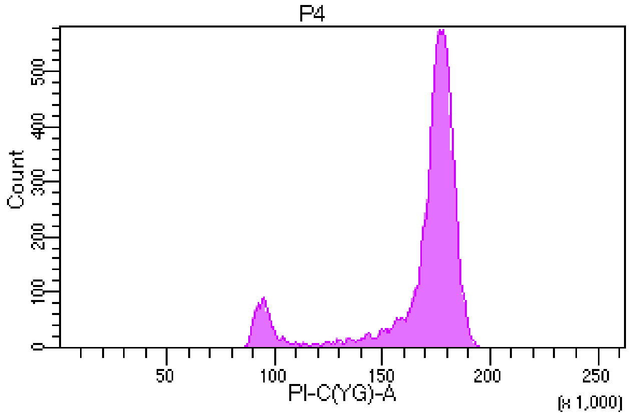

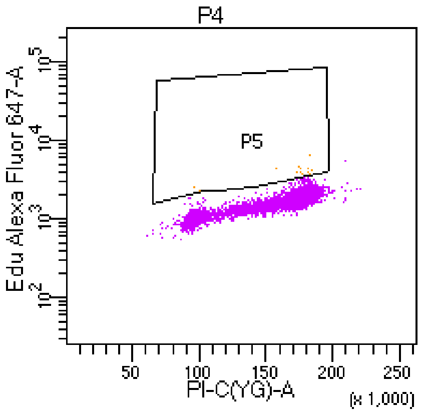

BD FACSDiva 8.0.1

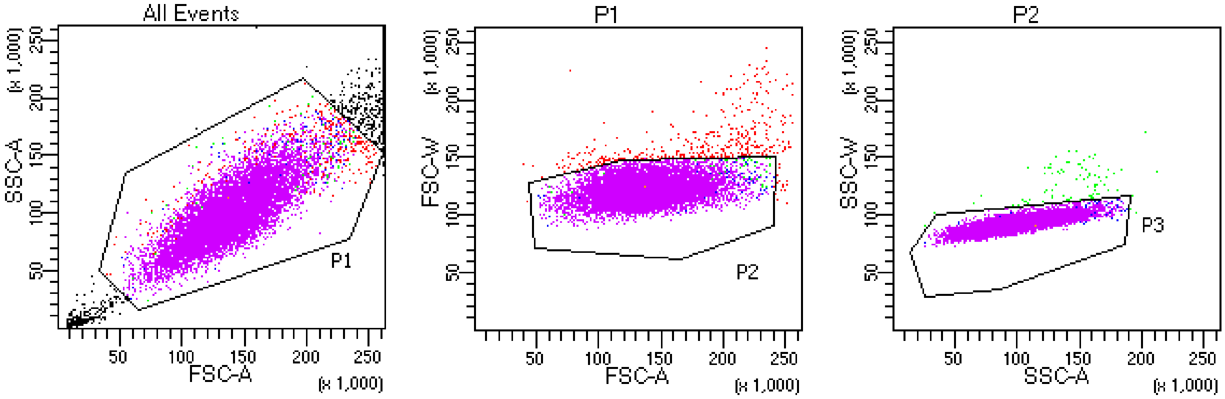

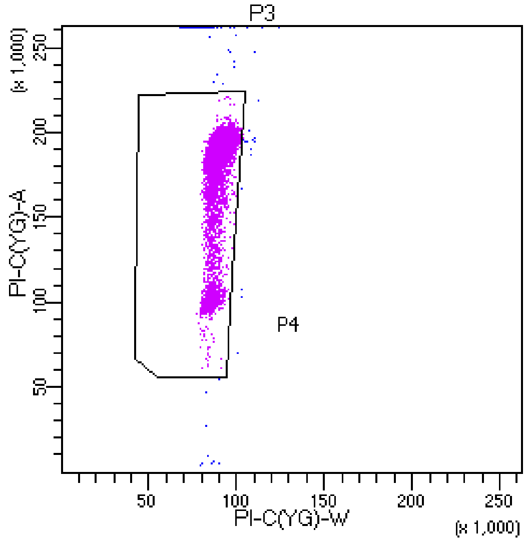

Tube: Tube_019

Population	#Events	%Parent	%Total
All Events	12,724	####	100.0
P1	10,810	85.0	85.0
P2	10,394	96.2	81.7
P3	10,307	99.2	81.0
P4	10,181	98.8	80.0
P5	1	0.0	0.0
P6	0	0.0	0.0

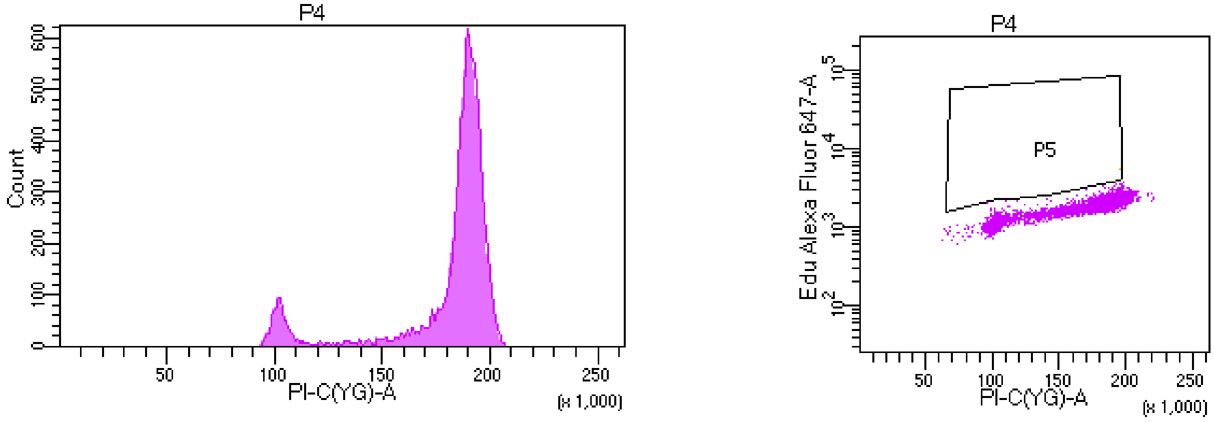

BD FACSDiva 8.0.1

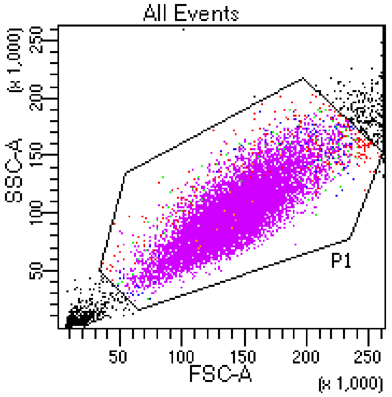

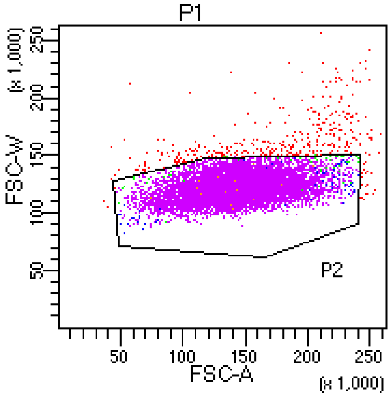

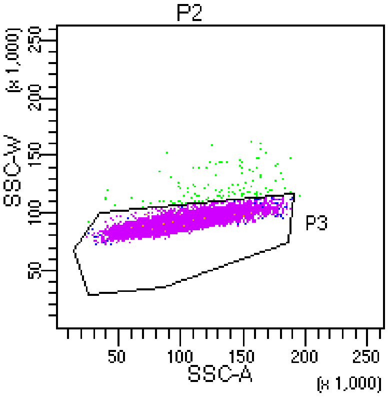

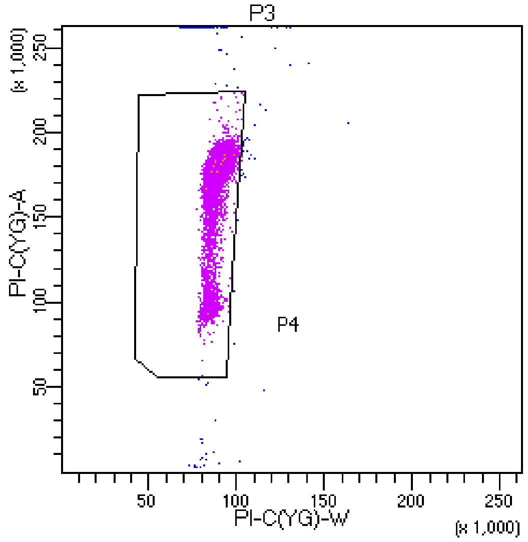

Tube: Tube_020

Population	#Events	%Parent	%Total
All Events	13,399	####	100.0
P1	10,927	81.6	81.6
P2	10,539	96.4	78.7
P3	10,413	98.8	77.7
P4	10,244	98.4	76.5
P5	14	0.1	0.1
P6	0	0.0	0.0

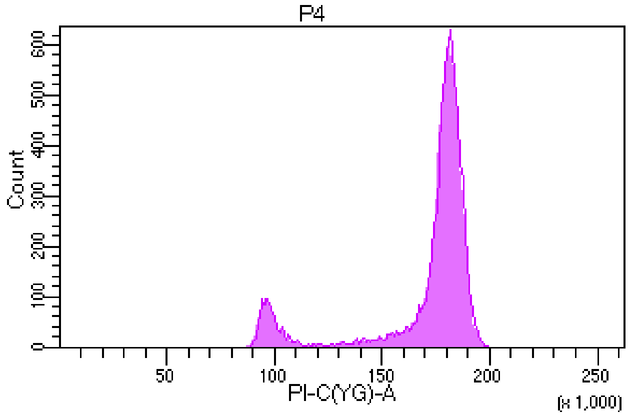

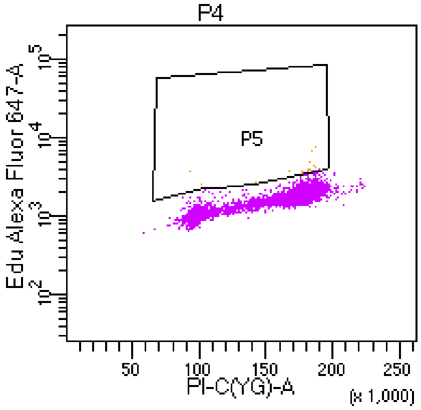

BD FACSDiva 8.0.1

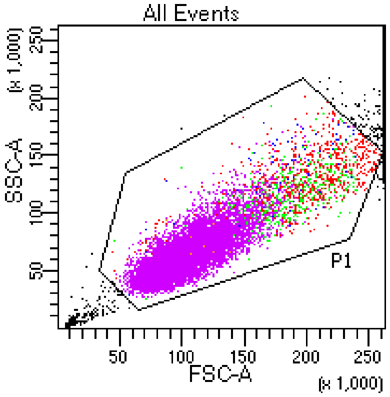

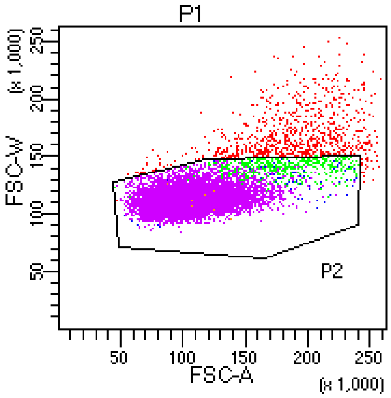

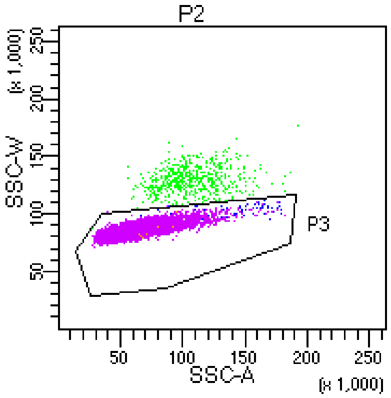

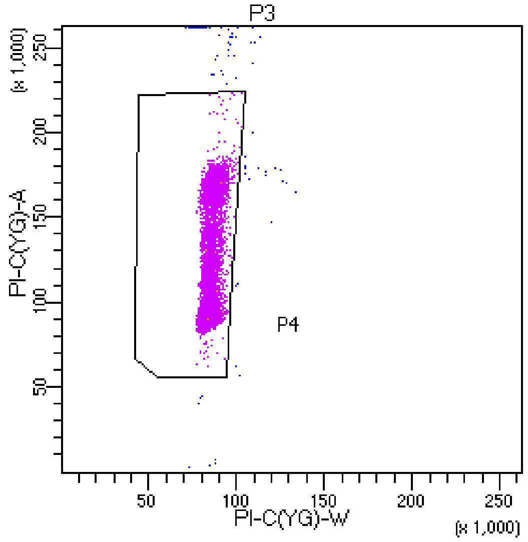

Tube: Tube_021

Population	#Events	%Parent	%Total
All Events	12,599	####	100.0
P1	11,512	91.4	91.4
P2	10,669	92.7	84.7
P3	10,109	94.8	80.2
P4	10,022	99.1	79.5
P5	7	0.1	0.1
P6	0	0.0	0.0

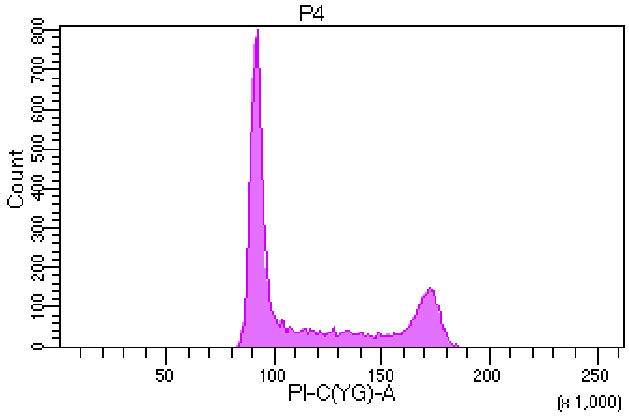

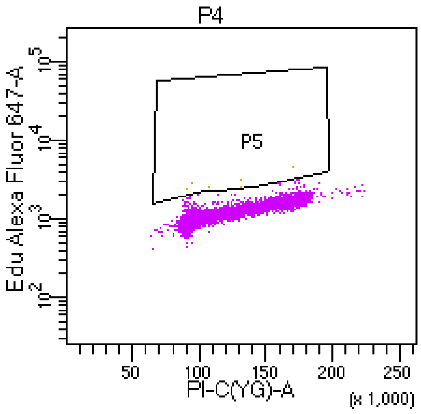

BD FACSDiva 8.0.1

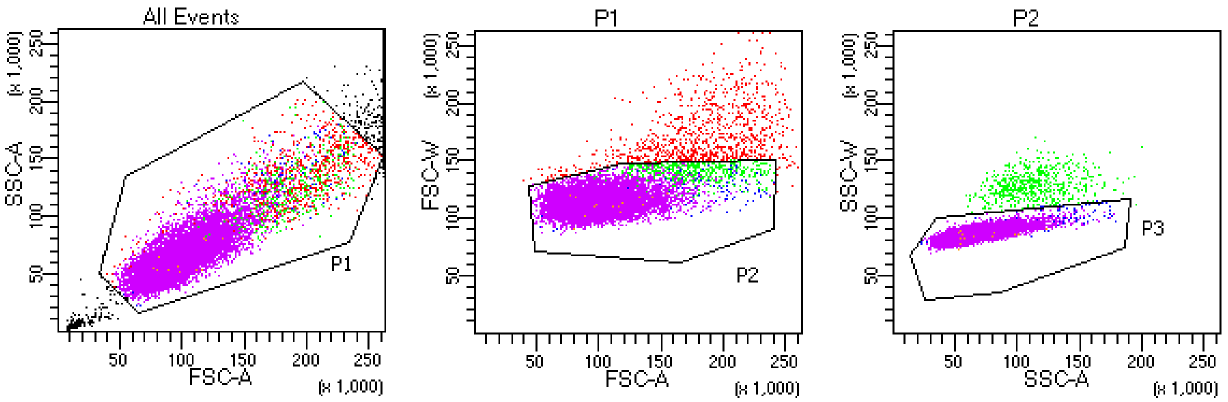

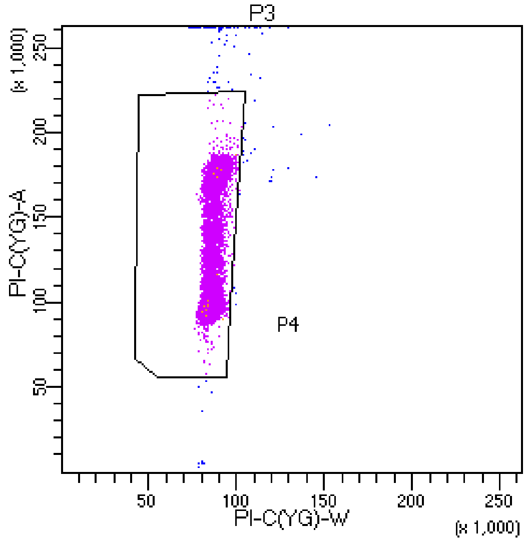

Tube: Tube_022

Population	#Events	%Parent	%Total
All Events	12,913	####	100.0
P1	11,730	90.8	90.8
P2	10,740	91.6	83.2
P3	10,137	94.4	78.5
P4	10,017	98.8	77.6
P5	14	0.1	0.1
P6	0	0.0	0.0

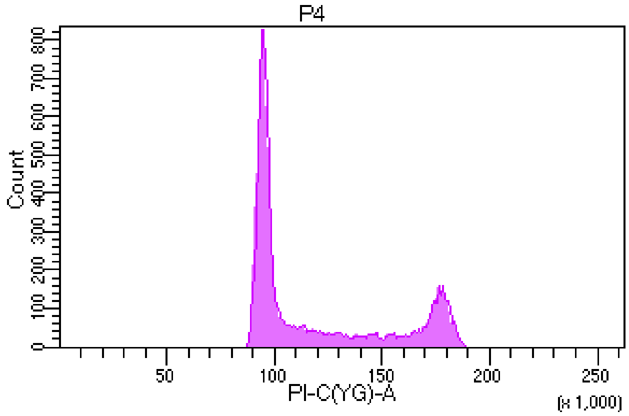

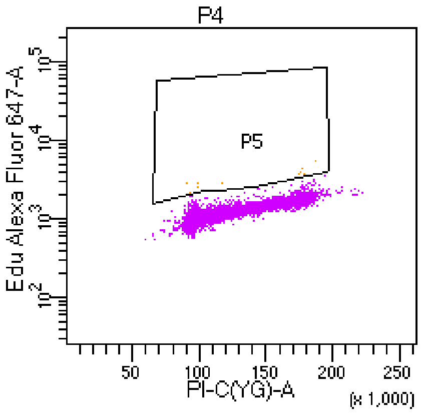

BD FACSDiva 8.0.1

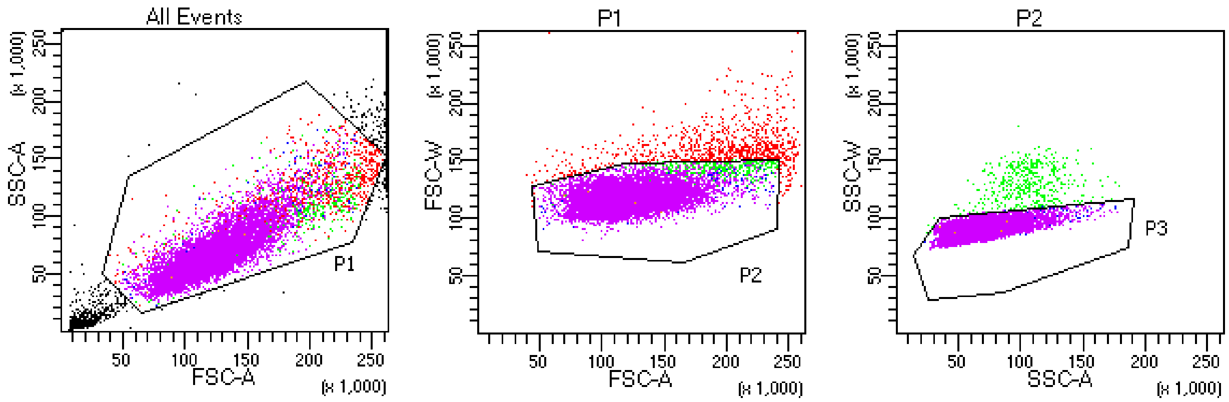

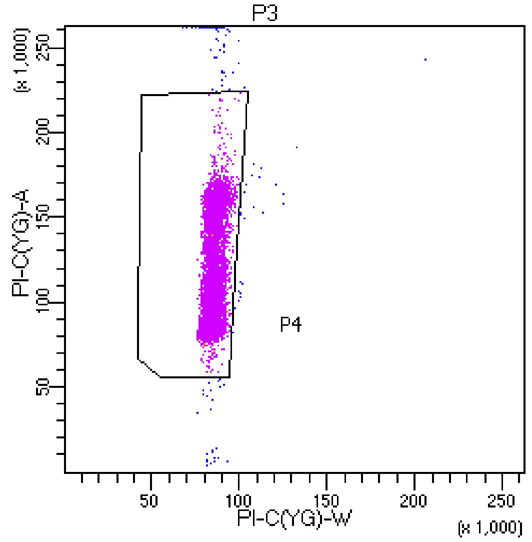

Tube: Tube_023

Population	#Events	%Parent	%Total
All Events	13,620	####	100.0
P1	11,543	84.8	84.8
P2	10,638	92.2	78.1
P3	10,195	95.8	74.9
P4	10,081	98.9	74.0
P5	4	0.0	0.0
P6	0	0.0	0.0

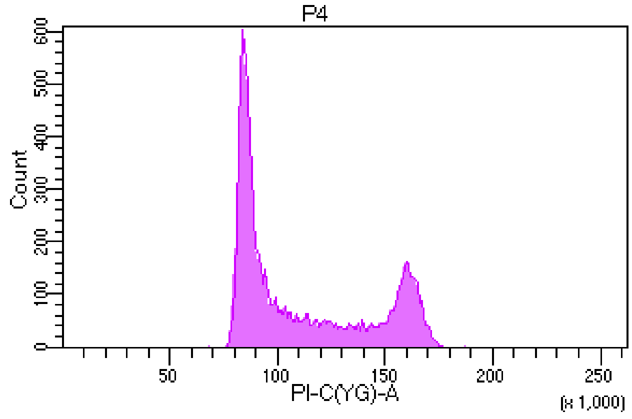

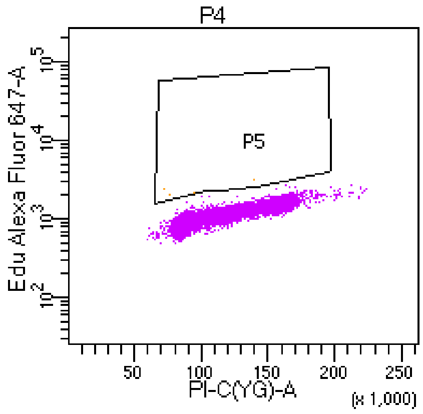

BD FACSDiva 8.0.1

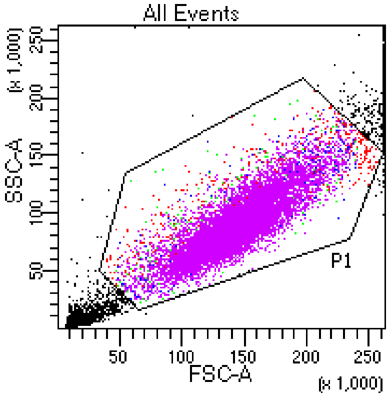

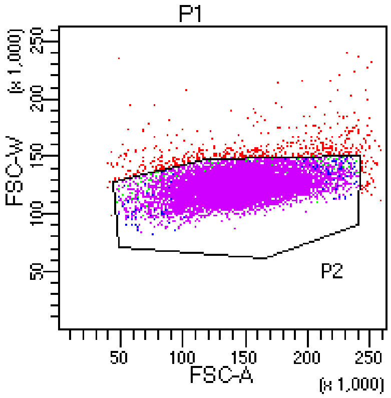

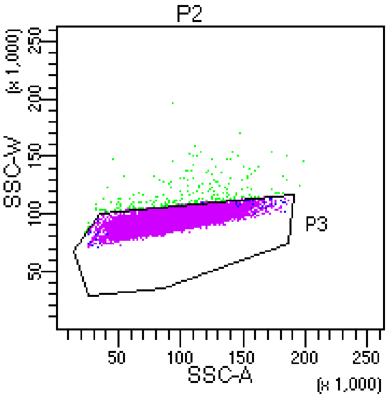

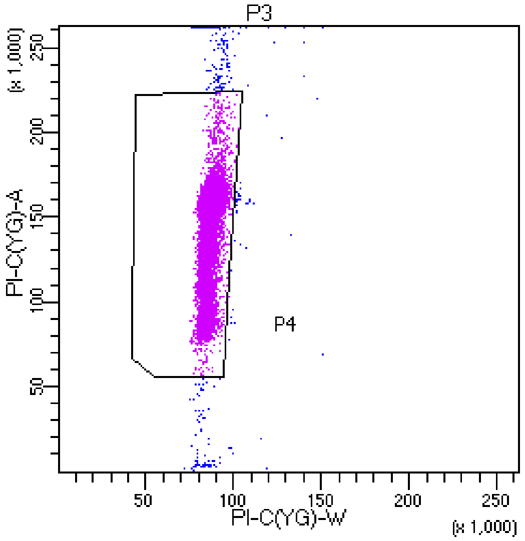

Tube: Tube_024

Population	#Events	%Parent	%Total
All Events	14,381	####	100.0
P1	11,461	79.7	79.7
P2	10,903	95.1	75.8
P3	10,675	97.9	74.2
P4	10,361	97.1	72.0
P5	0	0.0	0.0
P6	0	0.0	0.0

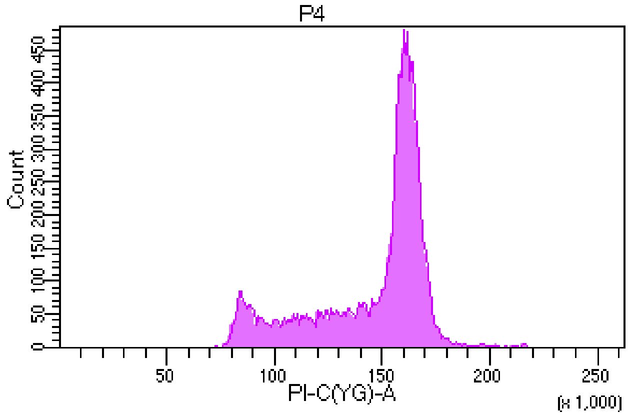

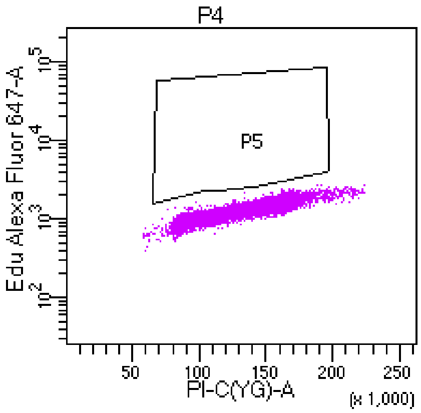

Printed on: Fri Dec 17, 2021 04:50:12 CST

BD FACSDiva 8.0.1

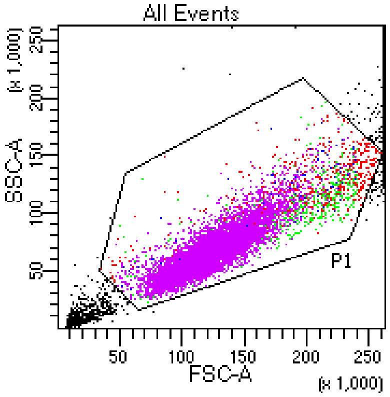

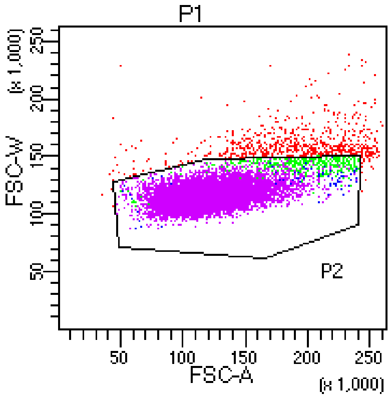

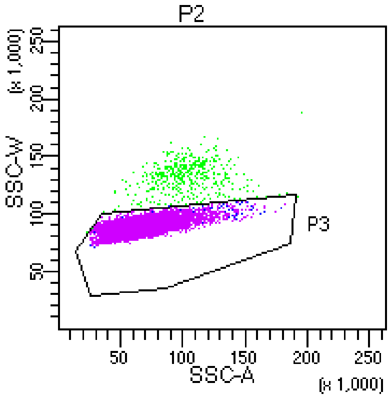

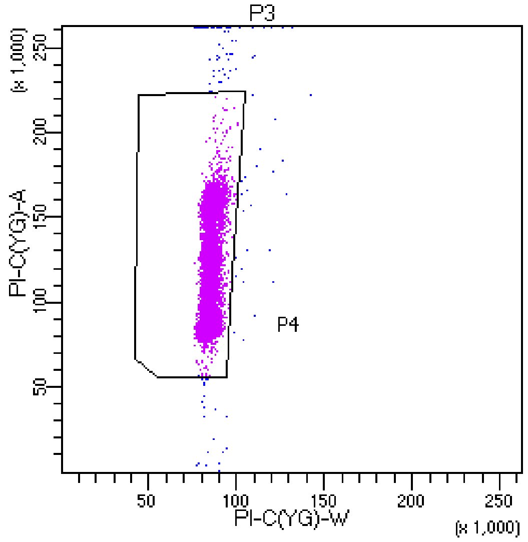

Tube: Tube_025

Population	#Events	%Parent	%Total
All Events	13,031	####	100.0
P1	11,320	86.9	86.9
P2	10,627	93.9	81.6
P3	10,160	95.6	78.0
P4	10,043	98.8	77.1
P5	0	0.0	0.0
P6	0	0.0	0.0

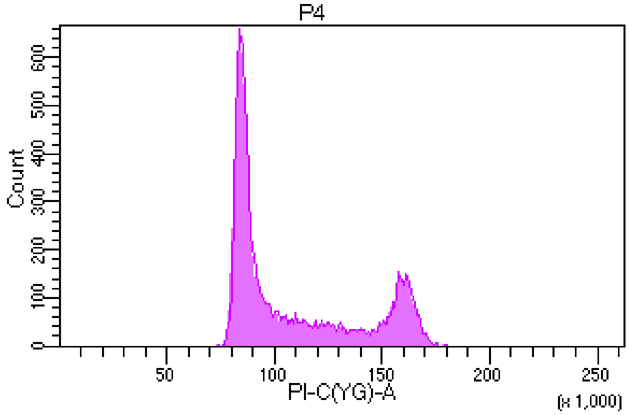

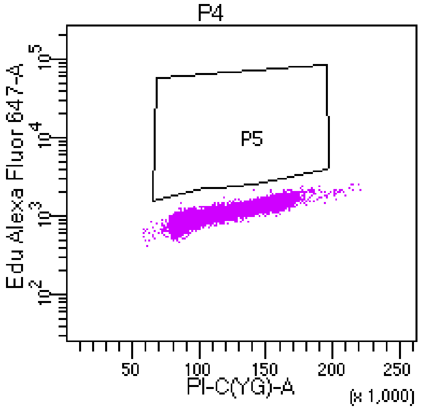

BD FACSDiva 8.0.1

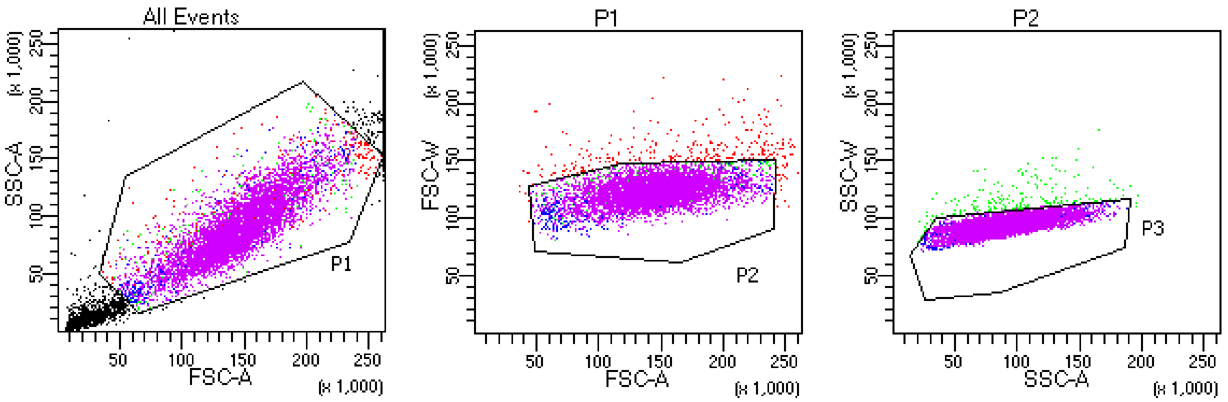

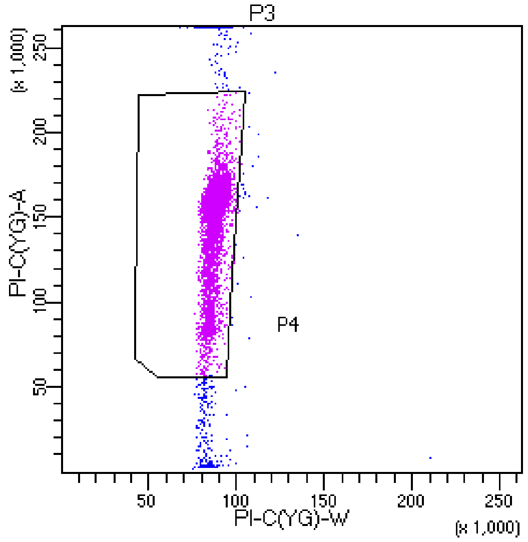

Tube: Tube_026

Population	#Events	%Parent	%Total
All Events	9,295	####	100.0
P1	6,894	74.2	74.2
P2	6,589	95.6	70.9
P3	6,385	96.9	68.7
P4	6,019	94.3	64.8
P5	0	0.0	0.0
P6	0	0.0	0.0

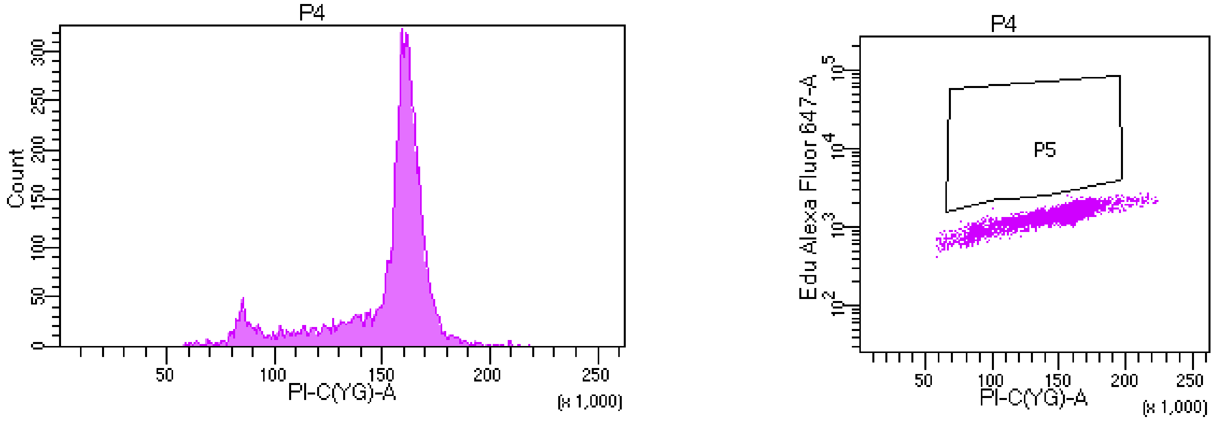

BD FACSDiva 8.0.1

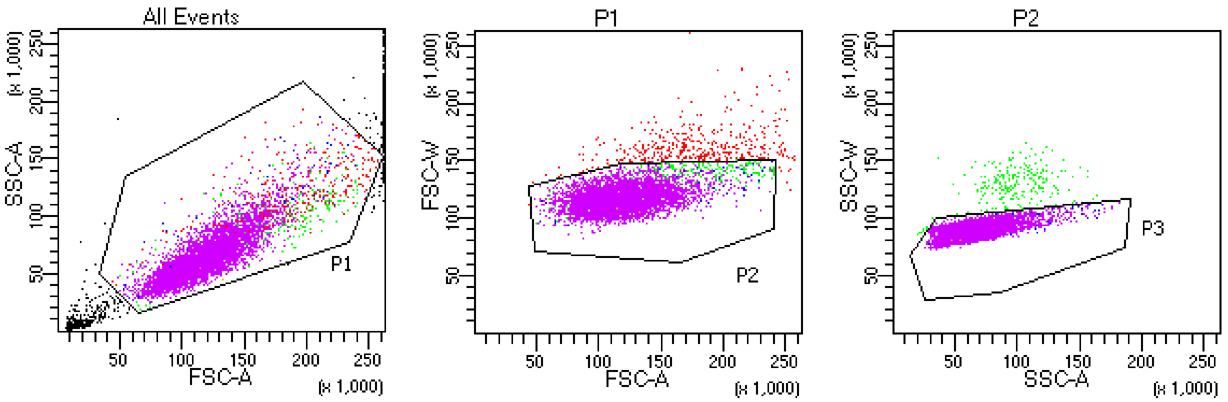

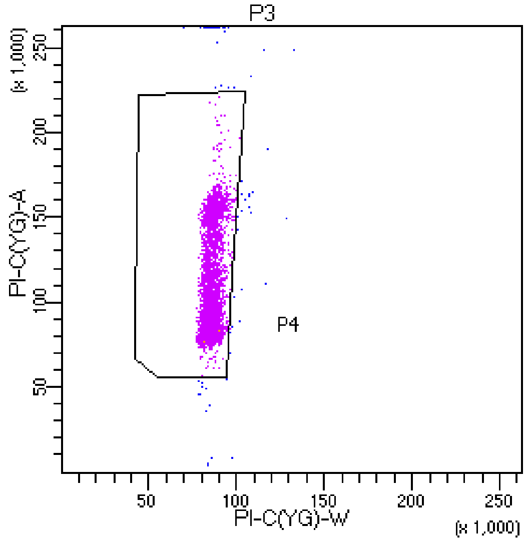

Tube: Tube_027

Population	#Events	%Parent	%Total
All Events	6,775	####	100.0
P1	6,094	89.9	89.9
P2	5,660	92.9	83.5
P3	5,421	95.8	80.0
P4	5,354	98.8	79.0
P5	2	0.0	0.0
P6	0	0.0	0.0

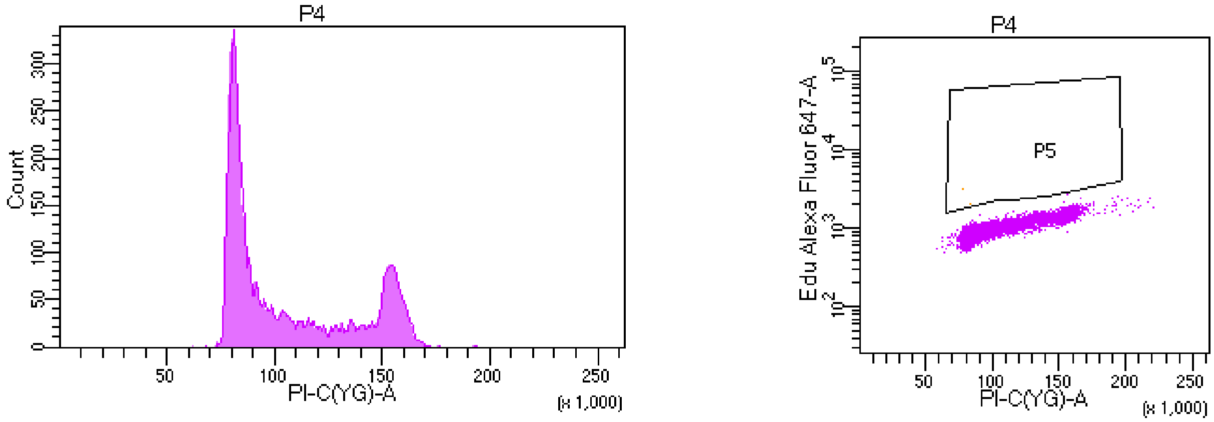

BD FACSDiva 8.0.1

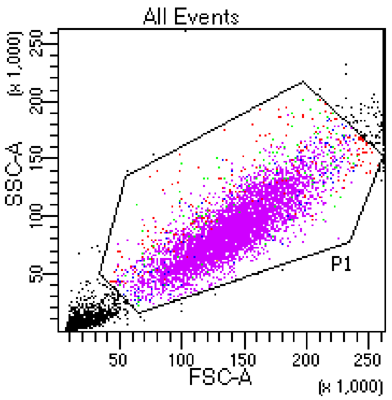

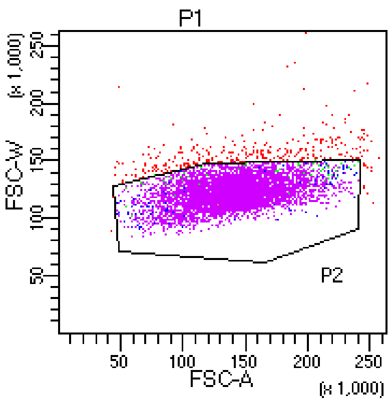

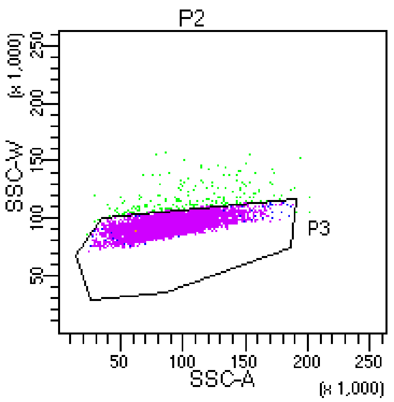

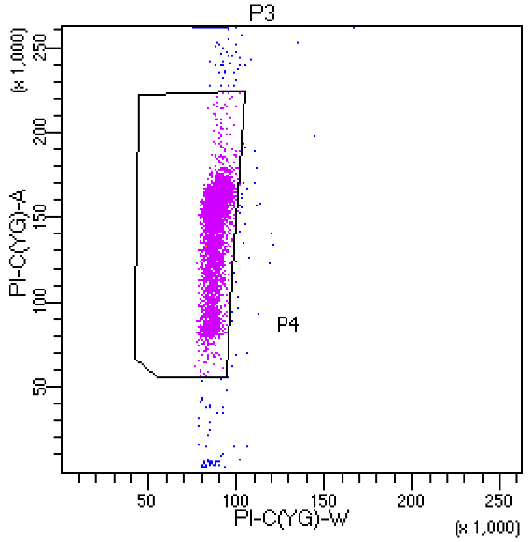

Tube: Tube_028

Population	#Events	%Parent	%Total
All Events	8,460	####	100.0
P1	5,999	70.9	70.9
P2	5,676	94.6	67.1
P3	5,474	96.4	64.7
P4	5,296	96.7	62.6
P5	1	0.0	0.0
P6	0	0.0	0.0

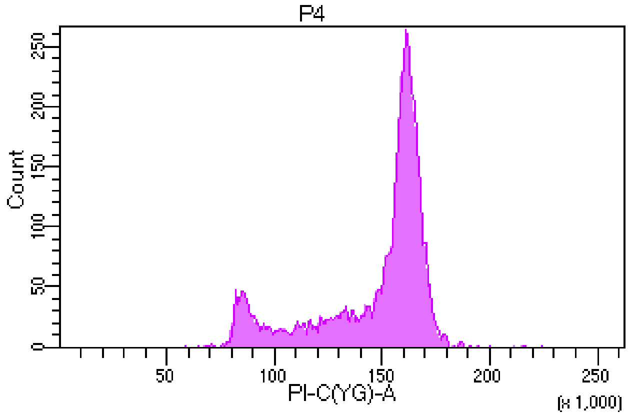

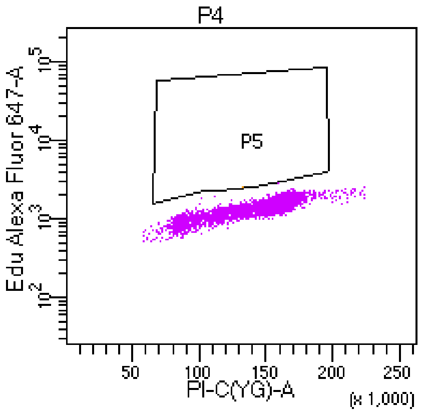

BD FACSDiva 8.0.1

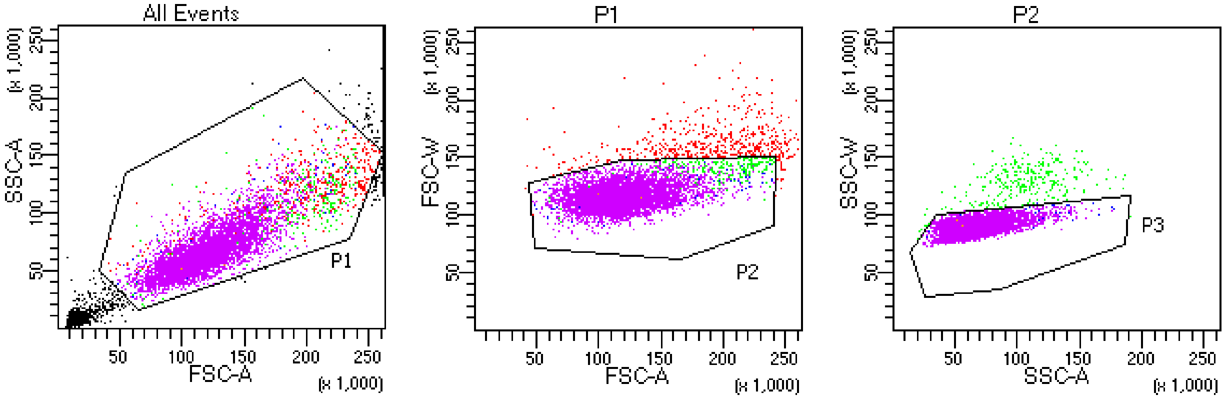

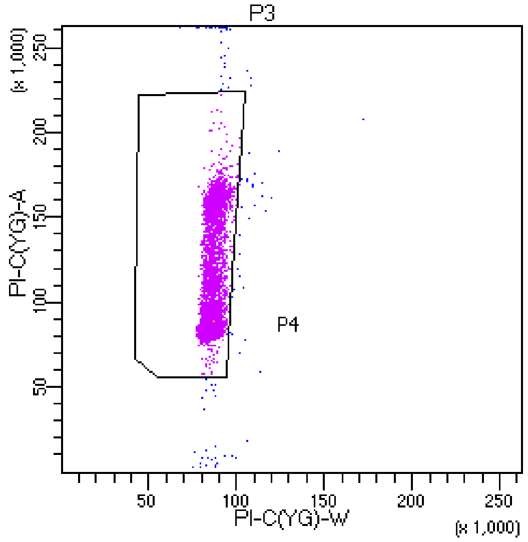

Tube: Tube_029

Population	#Events	%Parent	%Total
All Events	7,757	####	100.0
P1	6,077	78.3	78.3
P2	5,533	91.0	71.3
P3	5,235	94.6	67.5
P4	5,145	98.3	66.3
P5	2	0.0	0.0
P6	0	0.0	0.0

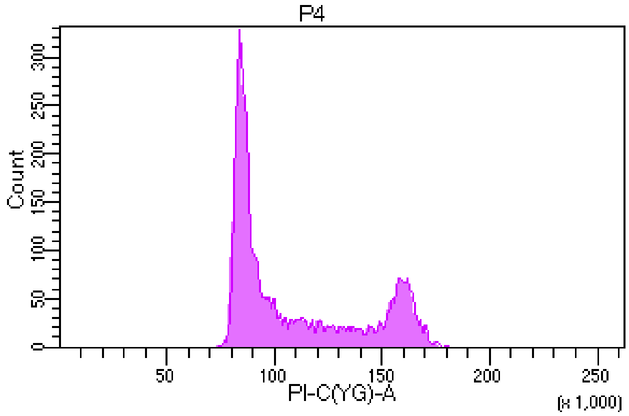

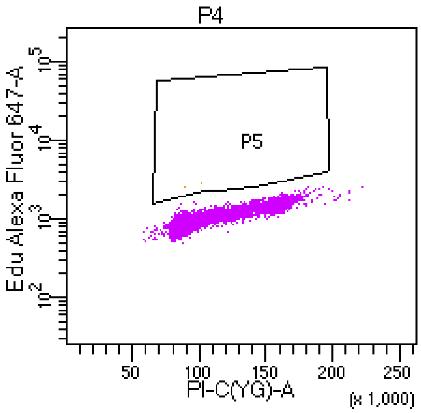

BD FACSDiva 8.0.1

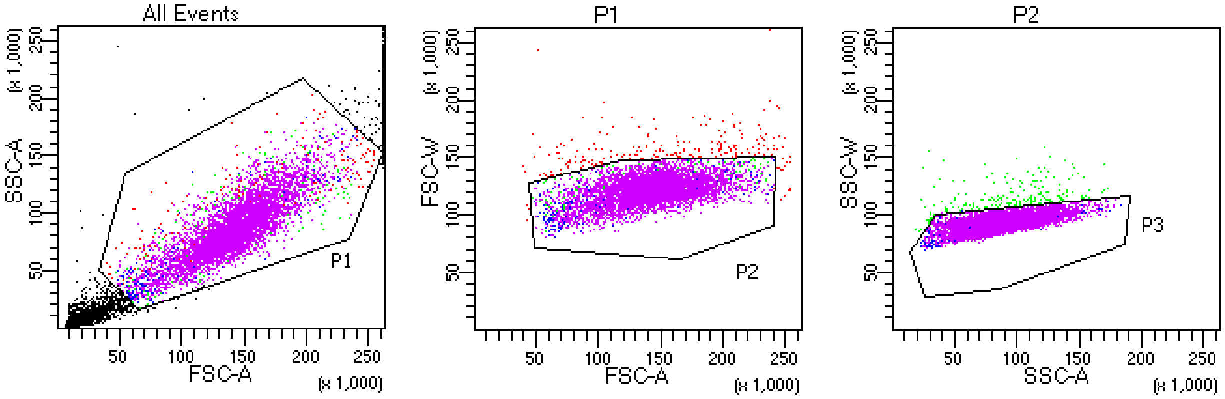

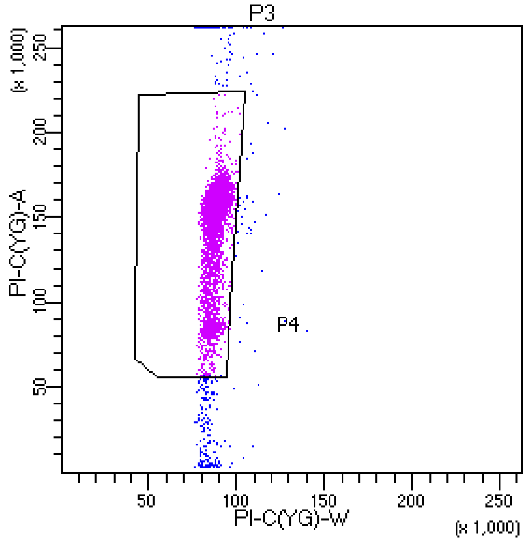

Tube: Tube_030

Population	#Events	%Parent	%Total
All Events	8,886	####	100.0
P1	5,851	65.8	65.8
P2	5,624	96.1	63.3
P3	5,413	96.2	60.9
P4	5,126	94.7	57.7
P5	0	0.0	0.0
P6	0	0.0	0.0

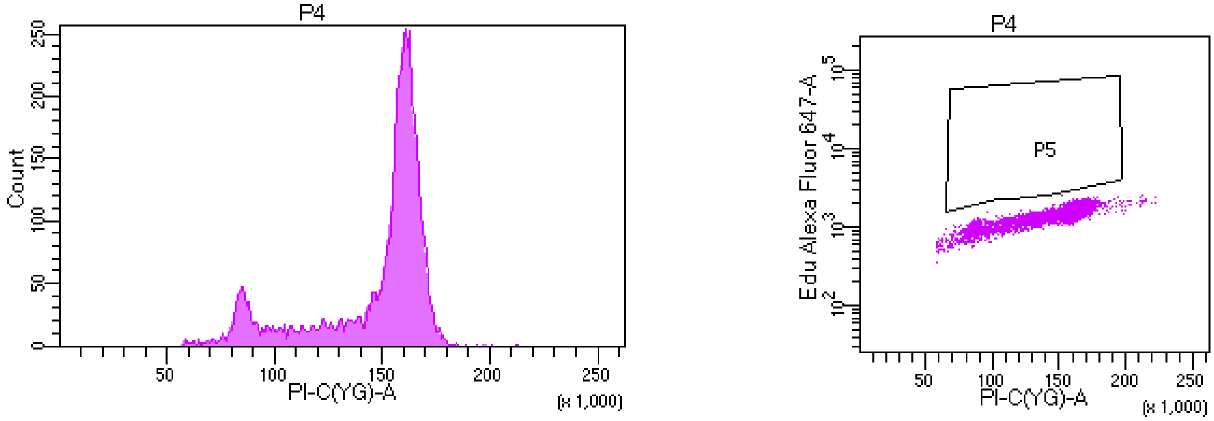

BD FACSDiva 8.0.1

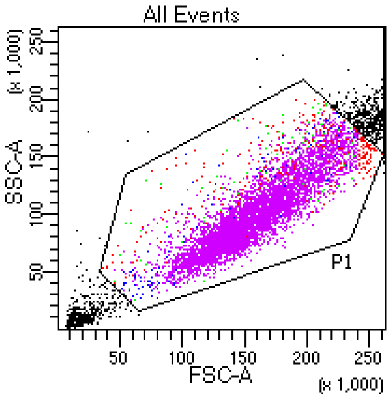

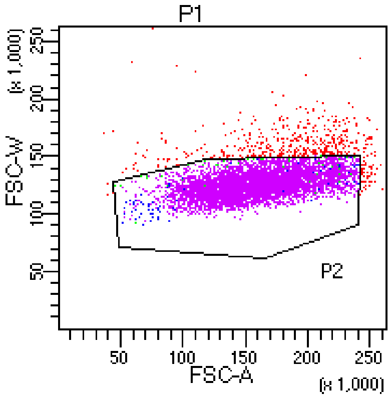

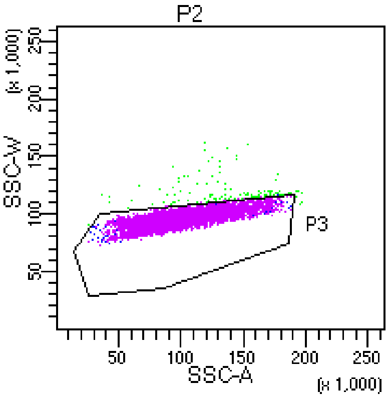

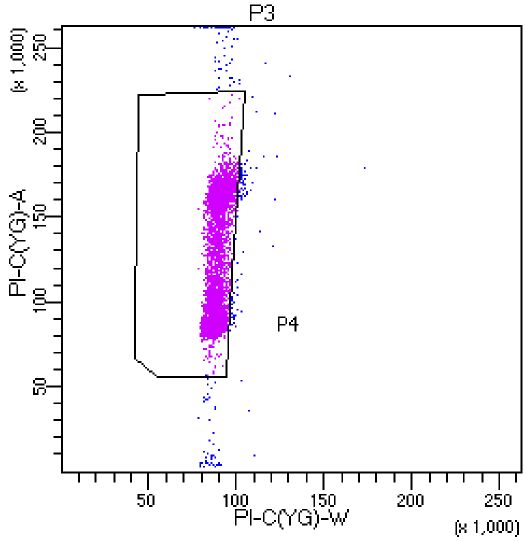

Tube: Tube_031

Population	#Events	%Parent	%Total
All Events	8,705	####	100.0
P1	6,303	72.4	72.4
P2	5,808	92.1	66.7
P3	5,687	97.9	65.3
P4	5,488	96.5	63.0
P5	0	0.0	0.0
P6	0	0.0	0.0

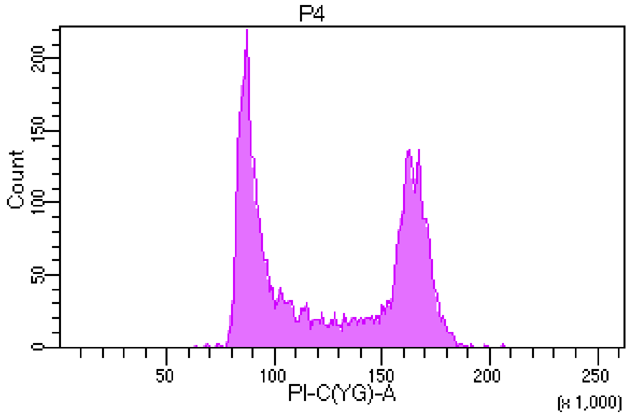

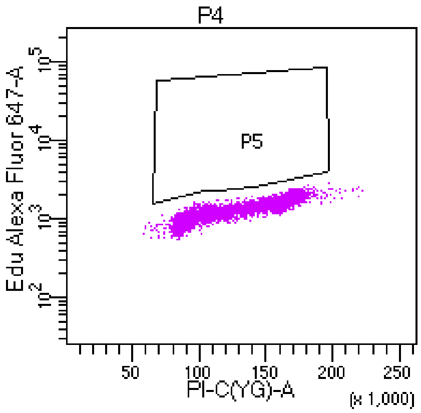

BD FACSDiva 8.0.1

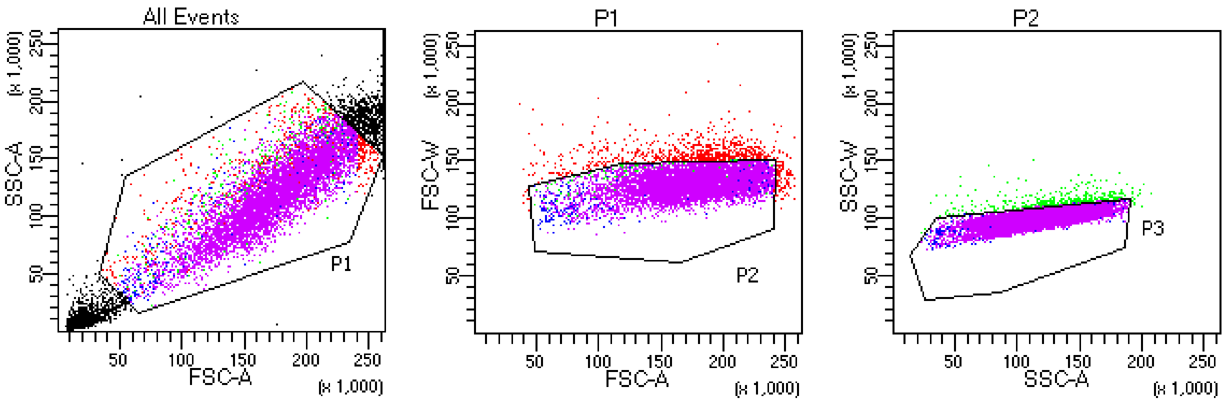

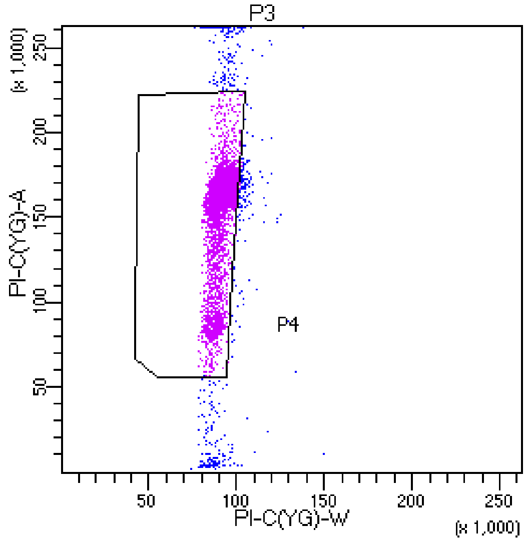

Tube: Tube_032

Population	#Events	%Parent	%Total
All Events	11,223	####	100.0
P1	7,344	65.4	65.4
P2	6,461	88.0	57.6
P3	6,062	93.8	54.0
P4	5,568	91.9	49.6
P5	0	0.0	0.0
P6	0	0.0	0.0

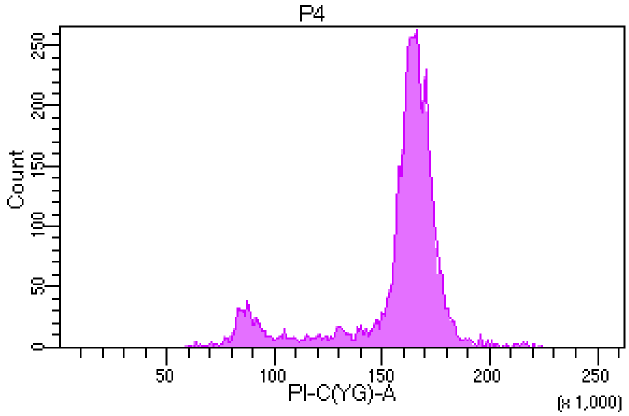

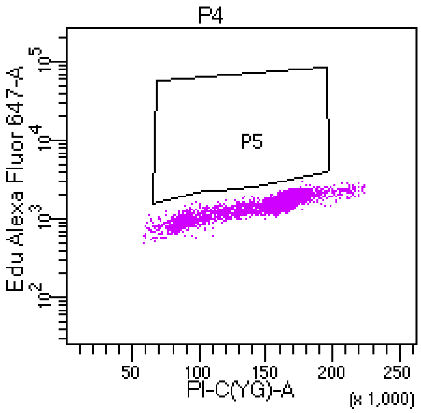

BD FACSDiva 8.0.1

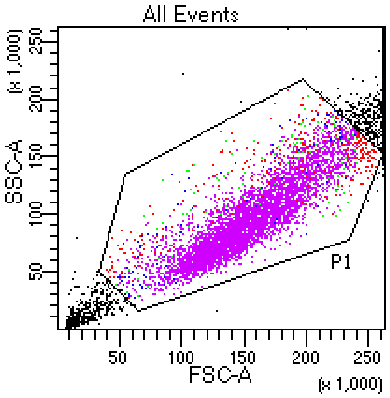

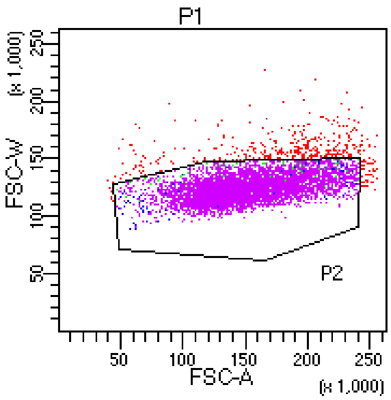

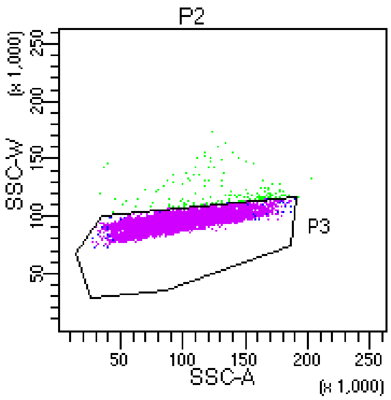

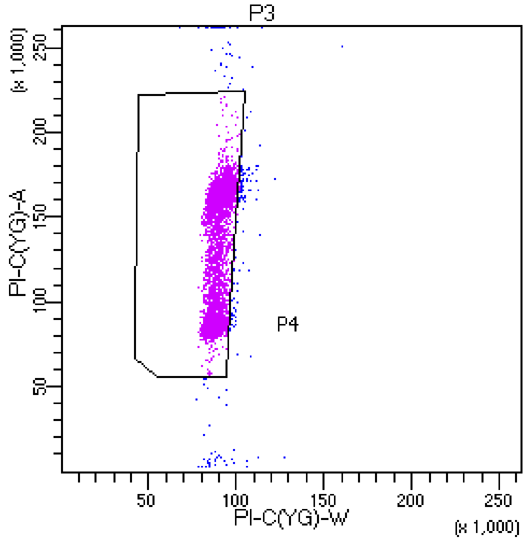

Tube: Tube_033

Population	#Events	%Parent	%Total
All Events	7,910	####	100.0
P1	5,801	73.3	73.3
P2	5,356	92.3	67.7
P3	5,205	97.2	65.8
P4	5,035	96.7	63.7
P5	0	0.0	0.0
P6	0	0.0	0.0

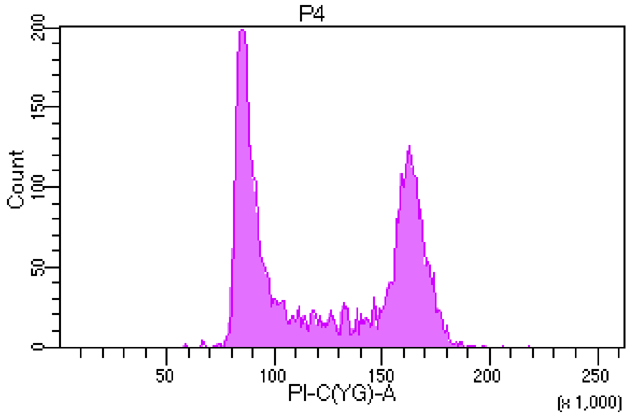

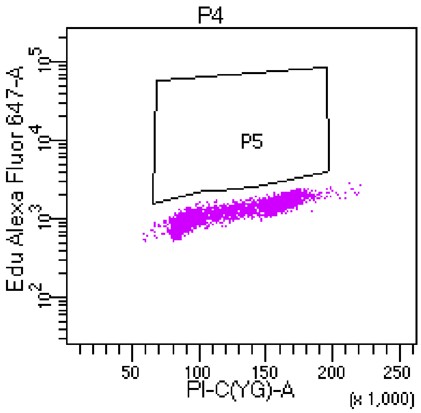

BD FACSDiva 8.0.1

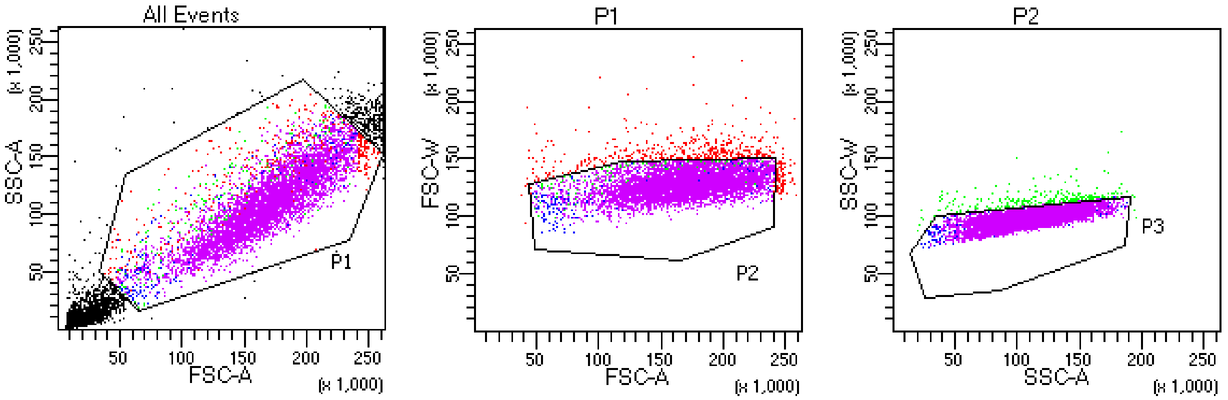

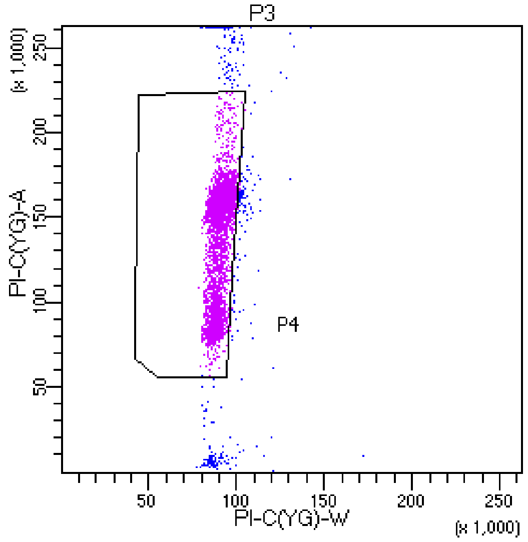

Tube: Tube_034

Population	#Events	%Parent	%Total
All Events	10,632	####	100.0
P1	6,177	58.1	58.1
P2	5,628	91.1	52.9
P3	5,346	95.0	50.3
P4	5,013	93.8	47.2
P5	0	0.0	0.0
P6	0	0.0	0.0

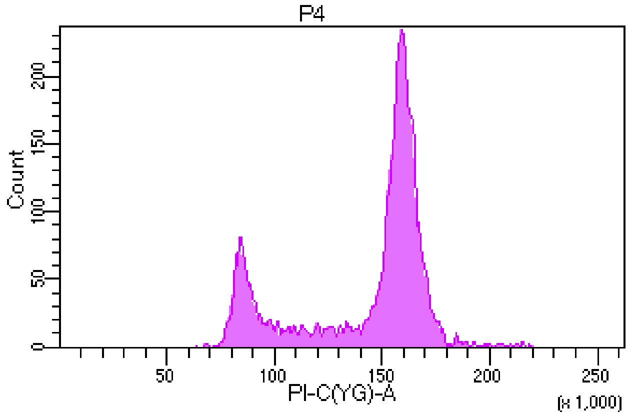

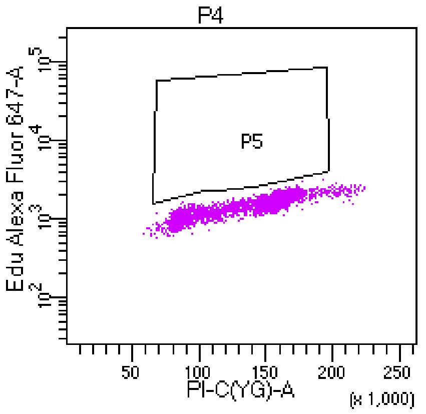

BD FACSDiva 8.0.1

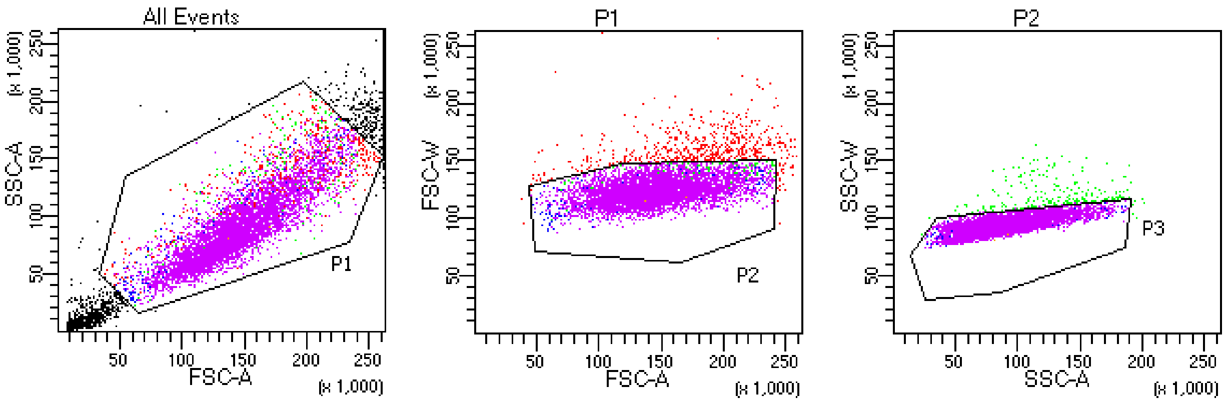

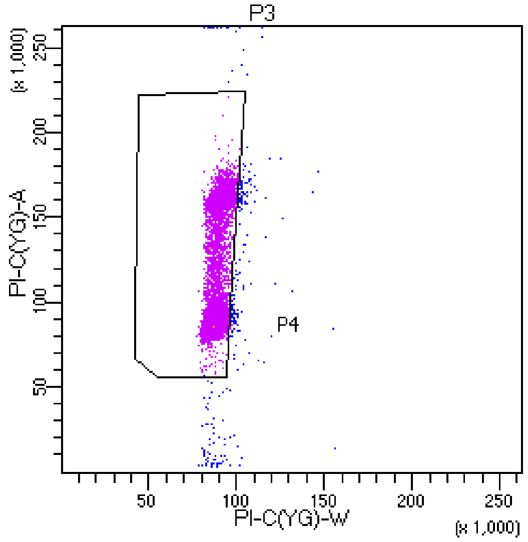

Tube: Tube_035

Population	#Events	%Parent	%Total
All Events	8,985	####	100.0
P1	6,096	67.8	67.8
P2	5,505	90.3	61.3
P3	5,289	96.1	58.9
P4	5,056	95.6	56.3
P5	2	0.0	0.0
P6	0	0.0	0.0

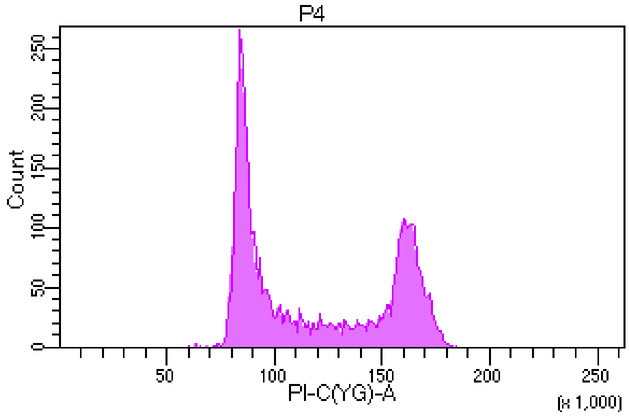

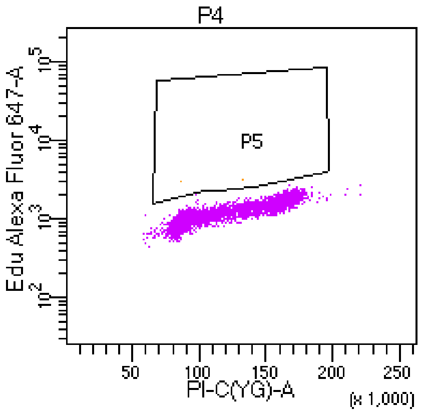

BD FACSDiva 8.0.1

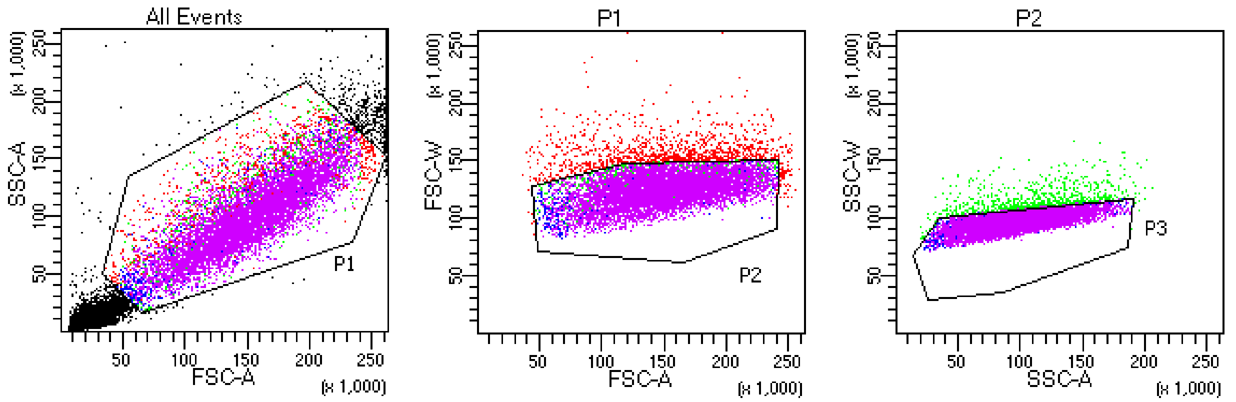

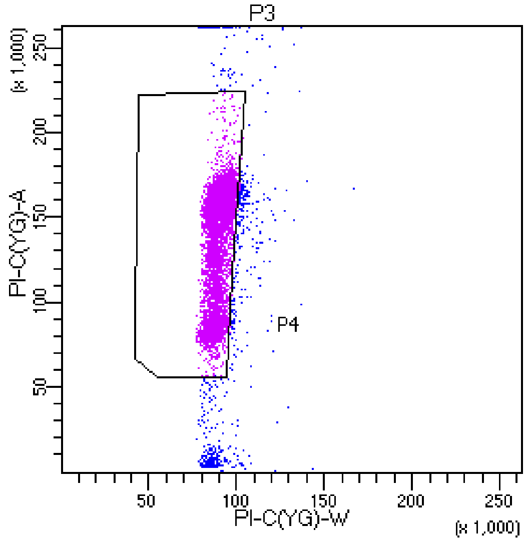

Tube: Tube_036

Population	#Events	%Parent	%Total
All Events	21,564	####	100.0
P1	9,723	45.1	45.1
P2	8,556	88.0	39.7
P3	7,795	91.1	36.1
P4	7,211	92.5	33.4
P5	0	0.0	0.0
P6	0	0.0	0.0

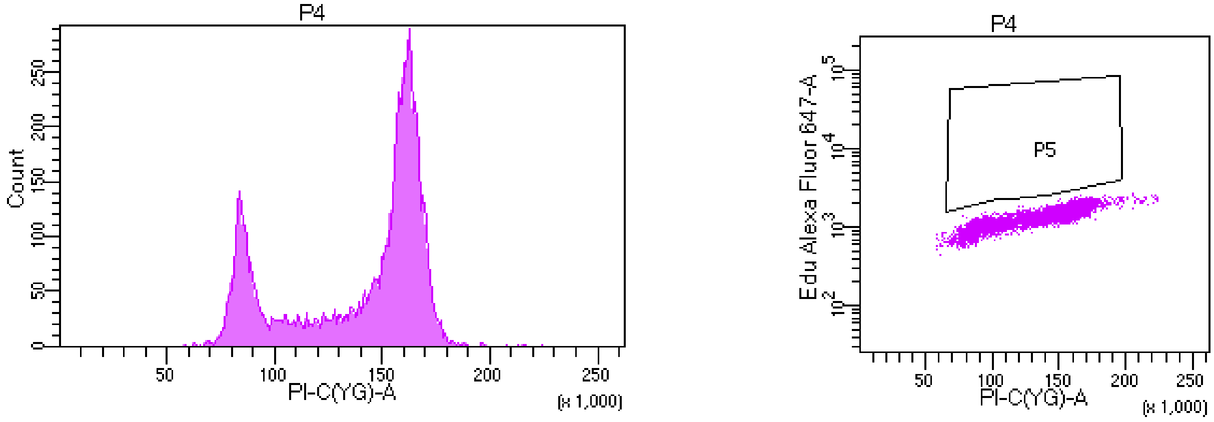

BD FACSDiva 8.0.1

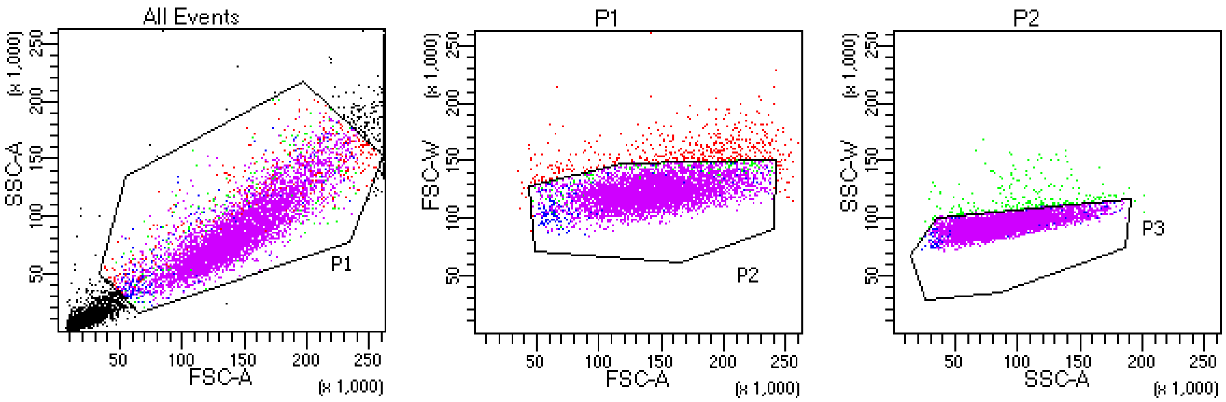

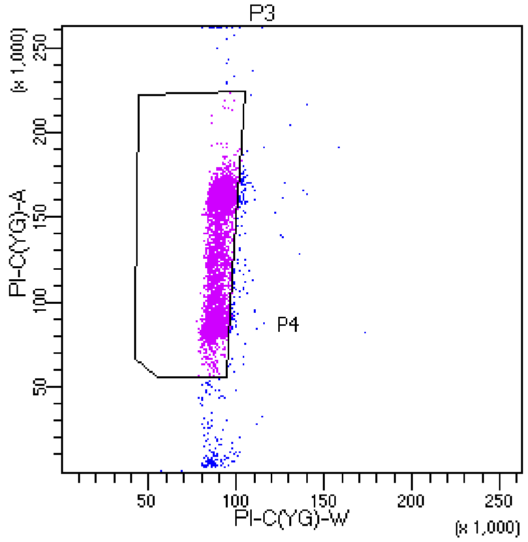

Tube: Tube_037

Population	#Events	%Parent	%Total
All Events	9,356	####	100.0
P1	6,030	64.5	64.5
P2	5,528	91.7	59.1
P3	5,297	95.8	56.6
P4	5,010	94.6	53.5
P5	0	0.0	0.0
P6	0	0.0	0.0

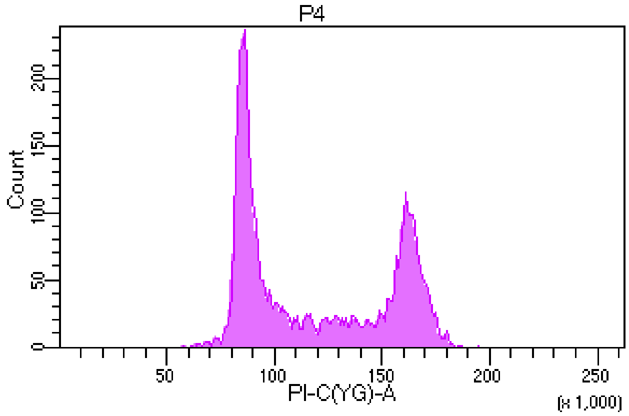

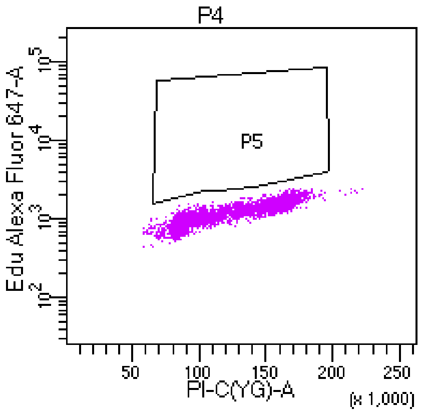

BD FACSDiva 8.0.1

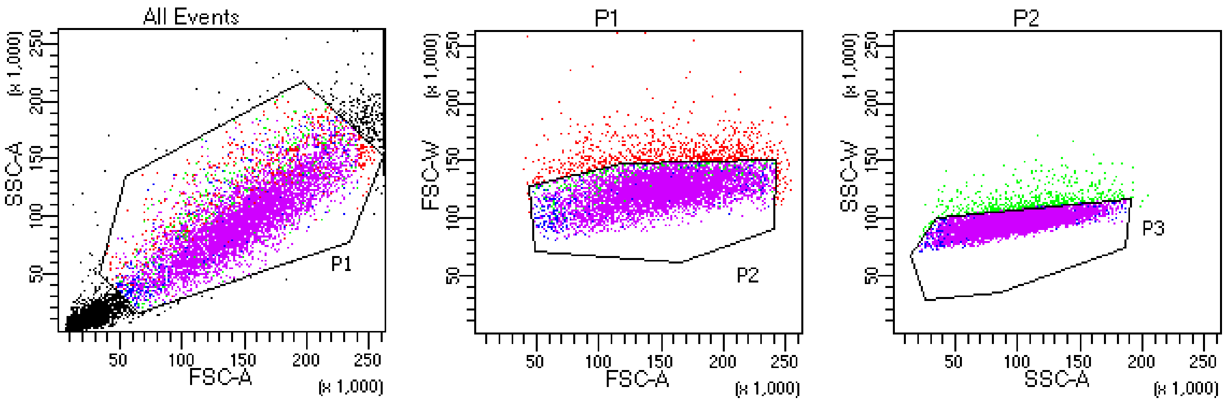

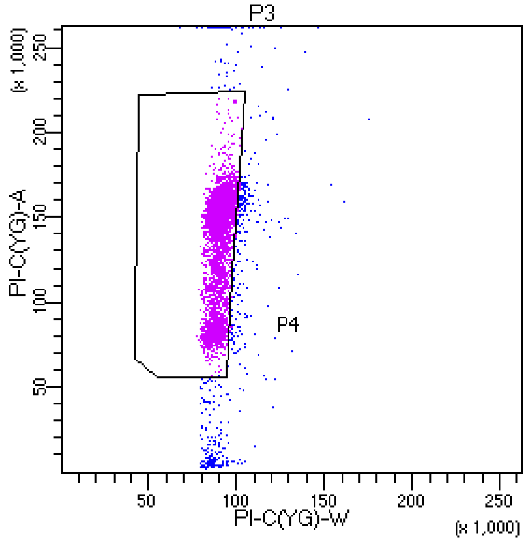

Tube: Tube_038

Population	#Events	%Parent	%Total
All Events	13,201	####	100.0
P1	6,897	52.2	52.2
P2	6,083	88.2	46.1
P3	5,523	90.8	41.8
P4	5,013	90.8	38.0
P5	0	0.0	0.0
P6	0	0.0	0.0

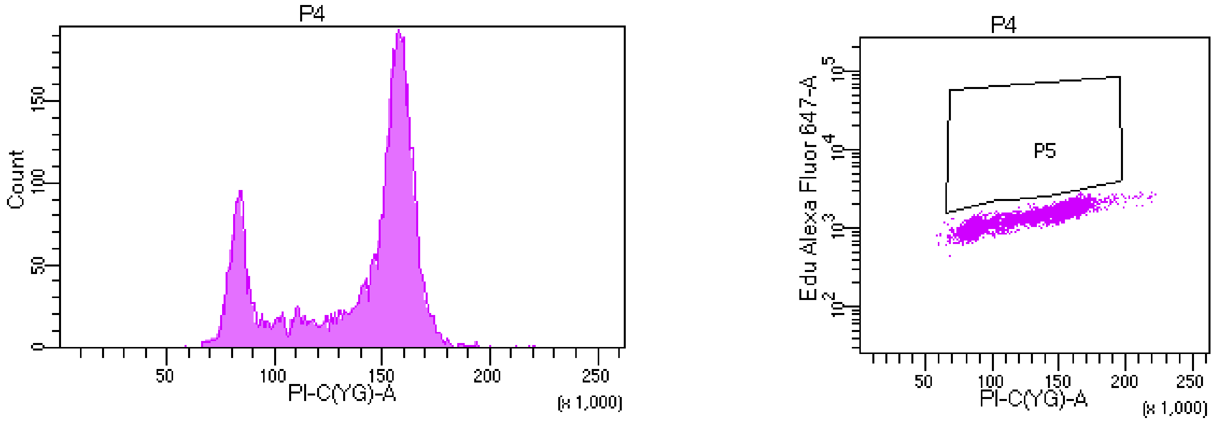

BD FACSDiva 8.0.1

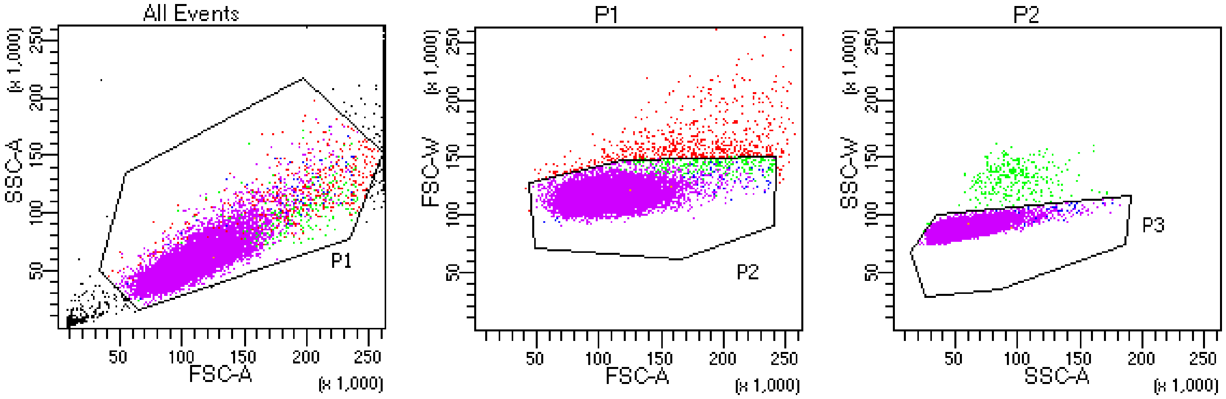

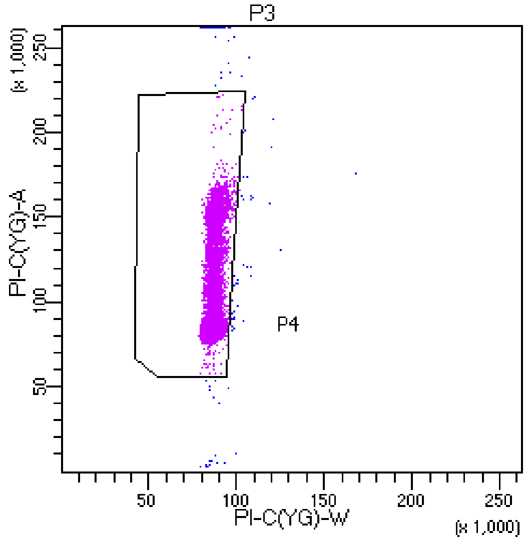

Tube: Tube_039

Population	#Events	%Parent	%Total
All Events	11,784	####	100.0
P1	11,076	94.0	94.0
P2	10,415	94.0	88.4
P3	10,108	97.1	85.8
P4	10,000	98.9	84.9
P5	2	0.0	0.0
P6	0	0.0	0.0

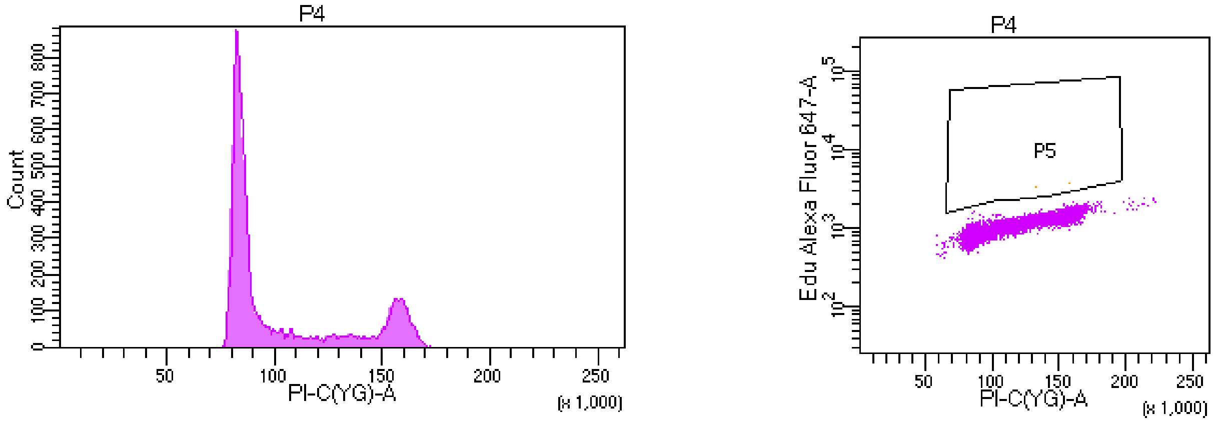

BD FACSDiva 8.0.1

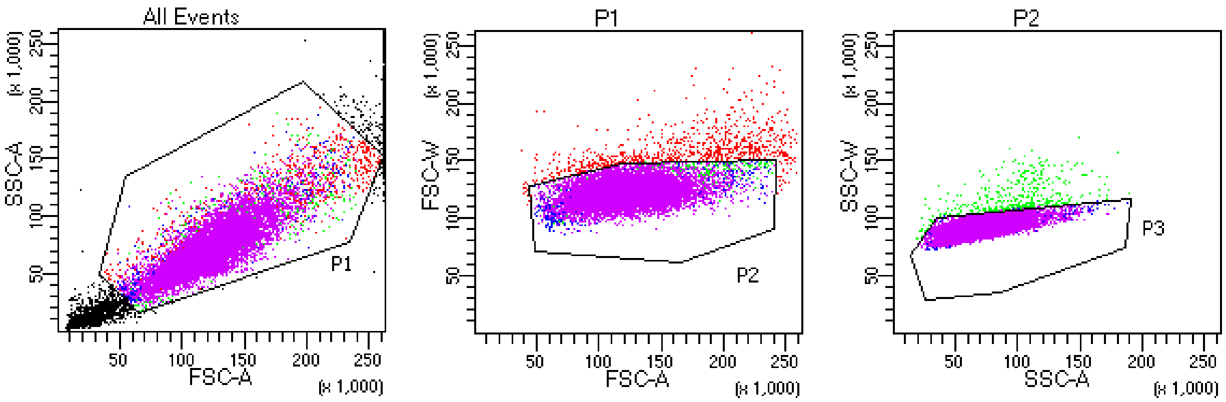

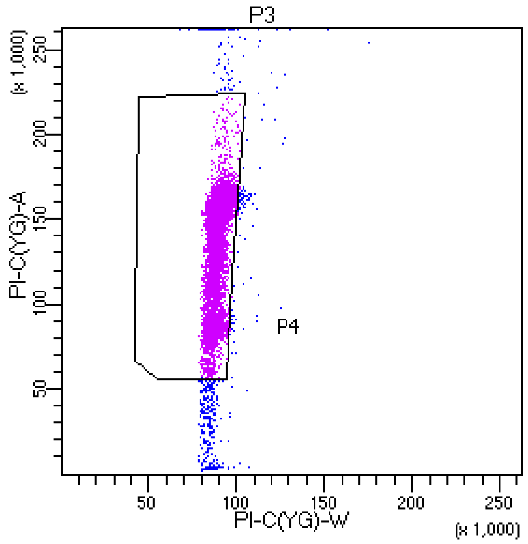

Tube: Tube_040

Population	#Events	%Parent	%Total
All Events	14,326	####	100.0
P1	11,792	82.3	82.3
P2	10,953	92.9	76.5
P3	10,547	96.3	73.6
P4	10,008	94.9	69.9
P5	0	0.0	0.0
P6	0	0.0	0.0

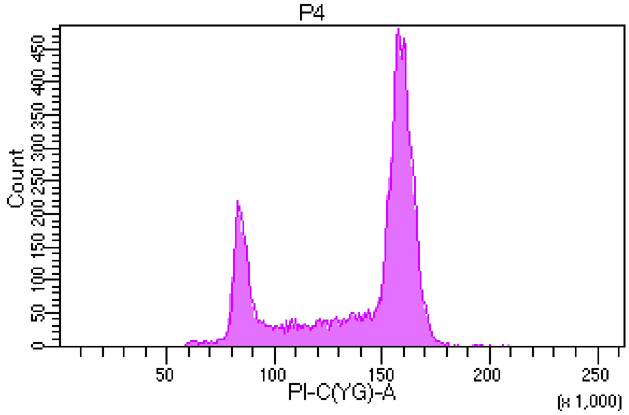

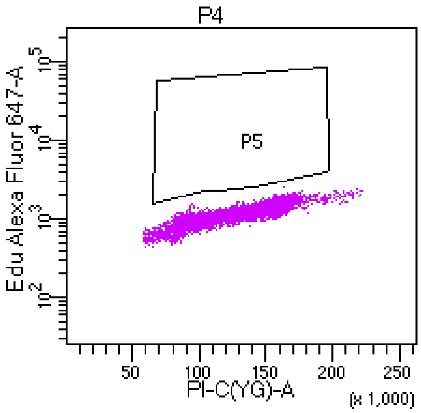

BD FACSDiva 8.0.1

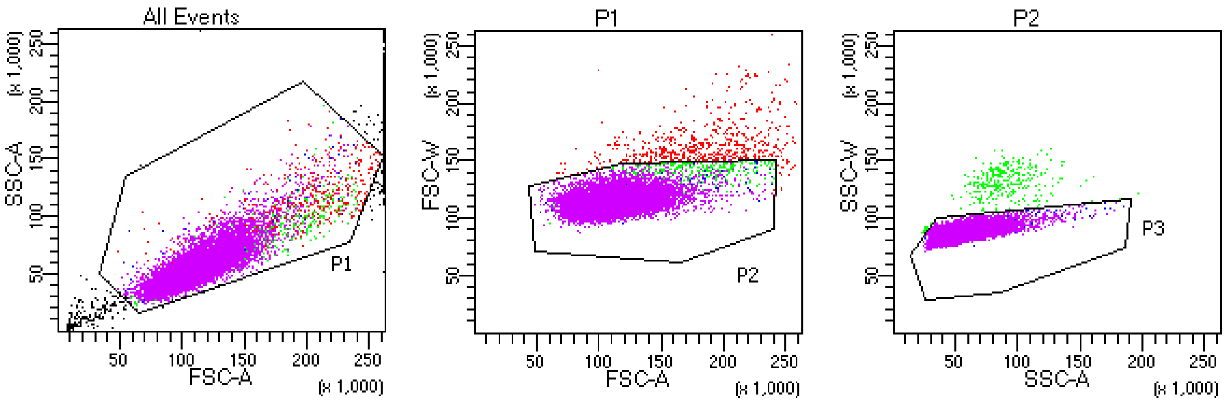

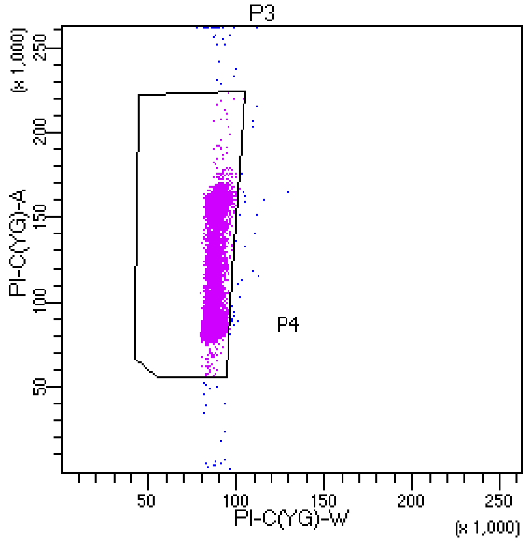

Tube: Tube_041

Population	#Events	%Parent	%Total
All Events	11,645	####	100.0
P1	11,048	94.9	94.9
P2	10,429	94.4	89.6
P3	10,082	96.7	86.6
P4	10,000	99.2	85.9
P5	0	0.0	0.0
P6	0	0.0	0.0

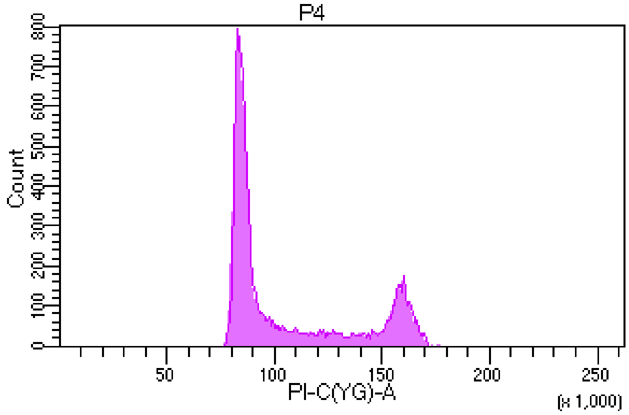

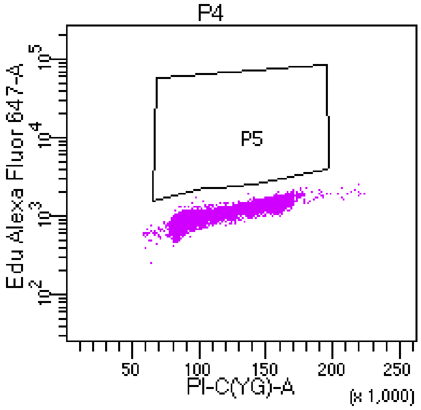

BD FACSDiva 8.0.1

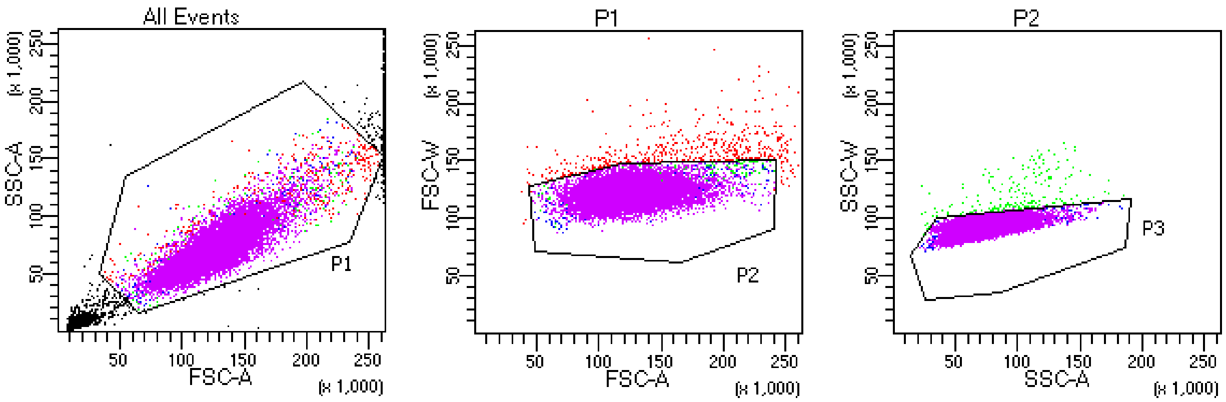

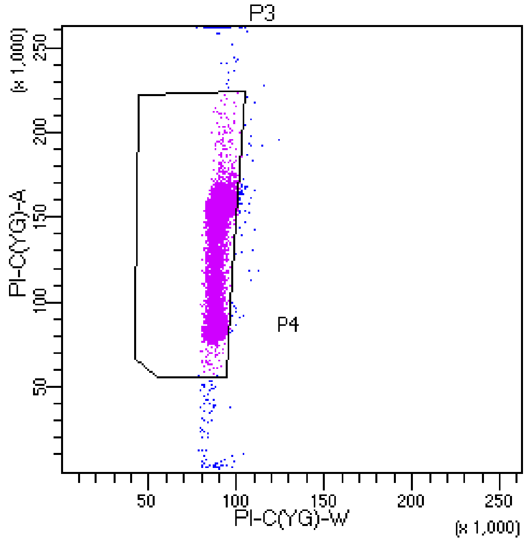

Tube: Tube_042

Population	#Events	%Parent	%Total
All Events	12,185	####	100.0
P1	10,960	89.9	89.9
P2	10,453	95.4	85.8
P3	10,244	98.0	84.1
P4	10,017	97.8	82.2
P5	0	0.0	0.0
P6	0	0.0	0.0

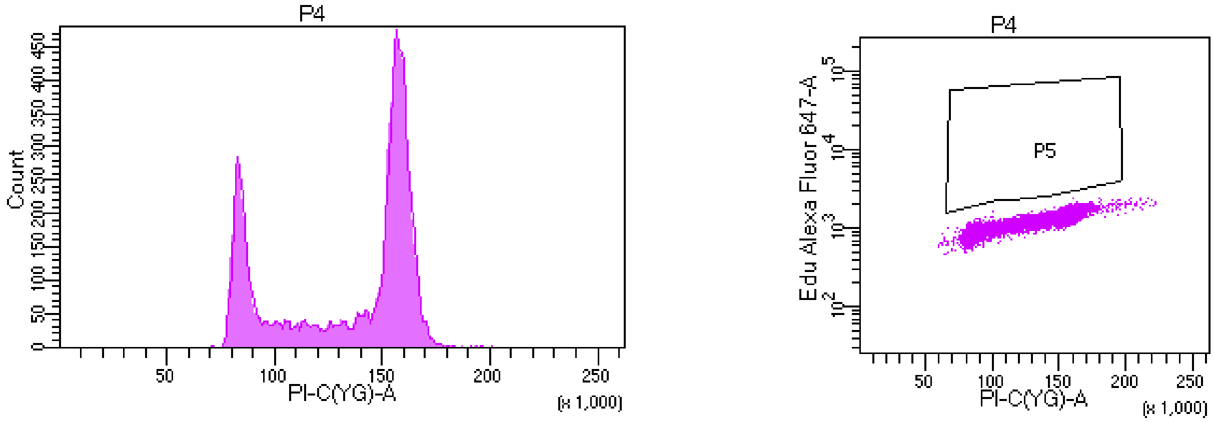

Supplement: Supplementary file 5 — Supplementary Data 2 [file 41467_2022_33428_MOESM5_ESM.pdf]
