# Supplementary material for: XPF activates break-induced telomere synthesis
Source: Nat Commun. 2022 Oct 2;13:5781. doi: 10.1038/s41467-022-33428-0 (PMC9527253; doi:10.1038/s41467-022-33428-0)
Supplement: Supplementary file 6 — Reporting Summary [file 41467_2022_33428_MOESM6_ESM.pdf]

nature portfolio

Corresponding author(s): Hsueh-Ping Catherine Chu

Last updated by author(s): Aug 28, 2022

Reporting Summary

Nature Portfolio wishes to improve the reproducibility of the work that we publish. This form provides structure for consistency and transparency in reporting. For further information on Nature Portfolio policies, see our Editorial Policies and the Editorial Policy Checklist.

Statistics

For all statistical analyses, confirm that the following items are present in the figure legend, table legend, main text, or Methods section.

n/a	Confirmed	
☐	☒	The exact sample size (n) for each experimental group/condition, given as a discrete number and unit of measurement
☐	☒	A statement on whether measurements were taken from distinct samples or whether the same sample was measured repeatedly
☐	☒	The statistical test(s) used AND whether they are one- or two-sided<br>*Only common tests should be described solely by name; describe more complex techniques in the Methods section.*
☐	☒	A description of all covariates tested
☐	☒	A description of any assumptions or corrections, such as tests of normality and adjustment for multiple comparisons
☐	☒	A full description of the statistical parameters including central tendency (e.g. means) or other basic estimates (e.g. regression coefficient) AND variation (e.g. standard deviation) or associated estimates of uncertainty (e.g. confidence intervals)
☐	☒	For null hypothesis testing, the test statistic (e.g. F, t, r) with confidence intervals, effect sizes, degrees of freedom and P value noted<br>*Give P values as exact values whenever suitable.*
☒	☐	For Bayesian analysis, information on the choice of priors and Markov chain Monte Carlo settings
☒	☐	For hierarchical and complex designs, identification of the appropriate level for tests and full reporting of outcomes
☐	☒	Estimates of effect sizes (e.g. Cohen's d, Pearson's r), indicating how they were calculated

Our web collection on statistics for biologists contains articles on many of the points above.

Software and code

Policy information about availability of computer code

Data collection	Softwares used are listed in Supplementary Data 1.
Data analysis	Data analysis is described in Methods section.

For manuscripts utilizing custom algorithms or software that are central to the research but not yet described in published literature, software must be made available to editors and reviewers. We strongly encourage code deposition in a community repository (e.g. GitHub). See the Nature Portfolio guidelines for submitting code & software for further information.

Data

Policy information about availability of data

All manuscripts must include a data availability statement. This statement should provide the following information, where applicable:

- Accession codes, unique identifiers, or web links for publicly available datasets
- A description of any restrictions on data availability
- For clinical datasets or third party data, please ensure that the statement adheres to our policy

The mass spectrometry proteomics data have been deposited to the ProteomeXchange Consortium via the PRIDE partner repository with the dataset identifier PXD028882.
Reviewer account details: Username: reviewer_pxd028882@ebi.ac.uk
Password: 6FVAXXjx

Field-specific reporting

Please select the one below that is the best fit for your research. If you are not sure, read the appropriate sections before making your selection.

- [x] Life sciences
- [ ] Behavioural & social sciences
- [ ] Ecological, evolutionary & environmental sciences

For a reference copy of the document with all sections, see nature.com/documents/nr-reporting-summary-flat.pdf

Life sciences study design

All studies must disclose on these points even when the disclosure is negative.

Sample size	For immunostaining experiments, sample sizes (n) represent the number of cells counted or the number of telomere foci counted per experimental group. The sample size determined based on previous experience and other published data using similar approaches of experiments (Pan et al., 2019 , Sci Rep; Silva et al., 2019, Nat Comm). The methods of statistics are described in the method section or each figure legend.
Data exclusions	No data were excluded from the analyses.
Replication	The exact number of biological replicates is indicated in each figure legend.
Randomization	Cells were plated evenly on the plate, and the images were taken randomly from the slide.
Blinding	The blinding was not required because the foci counting, qPCR, TRF assay and all other assays were analyzed automatically by computer.

Reporting for specific materials, systems and methods

We require information from authors about some types of materials, experimental systems and methods used in many studies. Here, indicate whether each material, system or method listed is relevant to your study. If you are not sure if a list item applies to your research, read the appropriate section before selecting a response.

Materials & experimental systems

n/a	Involved in the study	
☐	☒	Antibodies
☐	☒	Eukaryotic cell lines
☒	☐	Palaeontology and archaeology
☒	☐	Animals and other organisms
☒	☐	Human research participants
☒	☐	Clinical data
☒	☐	Dual use research of concern

Methods

n/a	Involved in the study	
☒	☐	ChIP-seq
☐	☒	Flow cytometry
☒	☐	MRI-based neuroimaging

Antibodies

Antibodies used

Mouse monoclonal anti-DNA-RNA Hybrid, clone S9.6 (Merck; Cat# MABE1095)
Rabbit monoclonal anti-GAPDH (14C10) (Cell Signaling; Cat# 2118)
Rabbit polyclonal anti-mCherry (GeneTex; Cat# GTX128508)
Rabbit polyclonal anti-RNase H1 [N2C3] (GeneTex; Cat# GTX117624)
Mouse monoclonal anti-α1c Tubulin (MH-87) (Santa Cruz; Cat# sc-134239)
Mouse monoclonal anti-FANCM Antibody, clone CV5.1 (Merck; Cat# MABC545)
Mouse monoclonal anti-phospho-Histone H2A.X (Ser139) (Merck; Cat# 05-636)
Rabbit monoclonal anti-phospho-Histone H2A.X (Ser139) (Cell Signaling; Cat# 9718T)
Mouse monoclonal anti-POLD3 (Abnova; Cat# H00010714-M01)
Mouse monoclonal anti-ERCC1 (Santa Cruz; Cat# sc-17809)
Mouse polyclonal anti-PML (Santa Cruz; Cat# sc-966)
Rabbit polyclonal anti-TRF2 (Novus; Cat# NB110-57130)
Mouse monoclonal anti-XPF (Thermo Fisher Scientific; Cat# MA5-12060)
Mouse monoclonal anti-BLM (Santa Cruz; Cat# sc-365753)
Rabbit polyclonal anti-BLM (Bethyl Laboratories; Cat# A300-110A)
Rabbit polyclonal anti-CSB (Bethyl Laboratories; Cat# A301-345A)
Rabbit polyclonal anti-SLX4 (Bethyl Laboratories; Cat# A302-269A)
Rabbit polyclonal anti-XPF (Bethyl Laboratories; Cat# A301-315A)
Mouse monoclonal anti-Rad51 (Abcam; Cat# ab213)
Mouse monoclonal anti-BRCA1 (Abcam; Cat# ab16780)

Mouse monoclonal anti-RPA70 (Santa Cruz; Cat# sc-28304)
Alexa Fluor 488 donkey anti-rabbit IgG (H+L) (Thermo Fisher Scientific; Cat# A21206)
Alexa Fluor 555 goat anti-mouse IgG (H+L) (Thermo Fisher Scientific; Cat# A21424)
Alexa Fluor 488 goat anti-mouse IgG (H+L) (Thermo Fisher Scientific; Cat# A11029)
Alexa Fluor 555 goat anti-rabbit IgG (H+L) (Thermo Fisher Scientific; Cat# A21429)
Alexa Fluor 647 goat anti-Rabbit IgG (H+L) (Thermo Fisher Scientific; Cat# A21245)
Goat anti-rabbit IgG (H+L), horseradish peroxidase conjugate (Thermo Fisher Scientific; Cat# G21234)
Goat anti-mouse IgG (H+L), horseradish peroxidase conjugate (Thermo Fisher Scientific; Cat# G21040)

Validation

Validation for each antibody is provided as indicated below.
DNA-RNA Hybrid S9.6: A Abakir et al. Nat Genet (2020); DOI: 10.1038/s41588-019-0549-x
phospho-Histone H2A.X (mouse) and GAPDH: GPF Nader et al. Cell (2021); DOI: 10.1016/j.cell.2021.08.035
RNase H1, FANCM and POLD3: B Silva et al. Nat Commun (2019); DOI: 10.1038/s41467-019-10179-z
Tubulin: KJ Chojnacka et al. Mol Biol Cell (2022); DOI: 10.1091/mbc.E21-03-0143
phospho-Histone H2A.X (rabbit): R Singh et al. EMBO Mol Med (2019); DOI: 10.15252/emmm.201809960
TRF2, PML, BLM (rabbit), and mCherry: X Pan et al. Sci Rep (2019); DOI: 10.1038/s41598-019-55537-5
XPF (mouse): SL Chalasani et al. DNA Repair (Amst) (2018); DOI: 10.1016/j.dnarep.2018.05.002
ERCC1 and XPF (rabbit): H Zhang et al. Nucleic Acids Res (2019); DOI: 10.1093/nar/gkz769
BLM (mouse): C Xue et al. Nat Commun (2022); DOI: 10.1038/s41467-022-29937-7
CSB: Q Zhu et al. Cell Cycle (2020); DOI: 10.1080/15384101.2019.1695996
SLX4: DA Matos et al. Mol Cell (2020); DOI: 10.1016/j.molcel.2019.10.010
RAD51: TH Huang et al. Mol Cell (2020); DOI: 10.1016/j.molcel.2020.02.019
BRCA1: NL Batenburg et al. Nucleic Acids Res (2019); DOI: 10.1093/nar/gkz784
RPA70: J Hao et al. EMBO J (2015); DOI: 10.15252/embj.201488016

Eukaryotic cell lines

Policy information about cell lines

Cell line source(s)	U2OS, WI38-VA, HeLa, HT-1080, SK-LMS-1
Authentication	No authentication was performed.
Mycoplasma contamination	All cell lines were tested negative for mycoplasma contamination.
Commonly misidentified lines (See ICLAC register)	No commonly misidentified lines were used in this study.

Flow Cytometry

Plots

Confirm that:

- [x] The axis labels state the marker and fluorochrome used (e.g. CD4-FITC).
- [x] The axis scales are clearly visible. Include numbers along axes only for bottom left plot of group (a 'group' is an analysis of identical markers).
- [x] All plots are contour plots with outliers or pseudocolor plots.
- [x] A numerical value for number of cells or percentage (with statistics) is provided.

Methodology

Sample preparation	Cells were fixed in EtOH and stained with PI for cell cycle analysis
Instrument	BD LSRFortessa
Software	Data were analyzed using ModFitLT V3.2 and BD FACSDiva softwares.
Cell population abundance	Cell populations with G1, S and G2 were shown in the Supplementary Fig. 7b, 7e.
Gating strategy	Cells without PI staining served as a negative control. Gating was based on SSC vs FCS density plot to remove debris. No antibodies are used in the flow cytometry.

- [x] Tick this box to confirm that a figure exemplifying the gating strategy is provided in the Supplementary Information.
